# The metabokine β-aminoisobutyric acid mediates exercise performance and skeletal muscle adaptation through a PGC1α-BAIBA-PPARδ axis

**DOI:** 10.1038/s41467-026-76307-8

**Published:** 2026-08-18

**Authors:** Helene N. Daou, Nicole T. Watt, Shaimaa A. Gad, Amanda DV MacCannell, Aysha Ali, Chew Cheng, Raquel L. Fernando, T. Simon Futers, Sumeet A. Khetarpal, Harrison Gallagher, Luis D. Chinait, Anselmo S. Moriscot, Pim Knuiman, Koen Manusama, Marco Mensink, Irvin Teh, Jurgen E. Schneider, Richard Cubbon, Guy S. Taylor, Daniel J. West, David J. Beech, T. Scott Bowen, Lee D. Roberts

**Affiliations:** 1 Leeds Institute of Cardiovascular and Metabolic Medicine, School of Medicine, University of Leeds, Leeds, UK; 2 Faculty of Biological Sciences, University of Leeds, Leeds, UK; 3 Faculty of Medicine, Mansoura University, Mansoura, Egypt; 4 Dana-Farber Cancer Institute and Harvard Medical School, Boston, MA USA; 5 Institute of Biomedical Sciences, University of Sao Paulo, Sao Paulo, Brazil; 6 Division of Human Nutrition, Wageningen University and Research, Wageningen, Netherlands; 7 Human Nutrition & Exercise Research Centre, Population Health Sciences Institute, Newcastle University, Newcastle upon Tyne, UK

**Keywords:** Homeostasis, Metabolic diseases, Energy metabolism

## Abstract

Exercise orchestrates an interorgan communication network, in which skeletal muscle releases signaling molecules known as myokines that contribute to exercise training-induced adaptations. We identified the muscle-derived metabolite beta-aminoisobutyric acid (BAIBA) as a regulator of adipose and hepatic metabolic responses to exercise. Here, we demonstrate that BAIBA regulates muscle metabolism, morphology and function via peroxisome proliferator-activated receptor delta (PPARδ) to determine exercise performance in mice. BAIBA mitigates muscle dysfunction in a mouse model of diabetes. Physiologically, BAIBA exists as D- and L- enantiomers. We identify L-BAIBA as the primary mediator of muscular effects. Knockdown of L-BAIBA’s biosynthetic enzyme, 4-aminobutyrate aminotransferase, in mouse hindlimb muscle impairs exercise-induced adaptations and performance gains. L-BAIBA regulates human myotube fibertype and differentiation markers through Mas-related G-protein coupled receptor D. In humans, plasma L-BAIBA correlates with aerobic fitness and increases with endurance exercise training. BAIBA acts through the PGC1α-BAIBA-PPARδ axis to facilitate muscle adaptation and exercise performance.

## Abstract (web summary)

Here, the authors identify the metabolite L-β-aminoisobutyric acid (L-BAIBA), released by muscle, as a central regulator of how muscles remodel, strengthen, and improve their endurance in response to exercise training.

## Introduction

Exercise is a systemic physiological process requiring co-ordination across multiple tissues and organs. Exercise training involves multiple bouts of activity that challenge whole-body physiology, driving adaptations across cells, tissues, and organs^1^. Exercise is also an effective intervention for the prevention or treatment of a range of diseases and pathological risk factors, including obesity, type 2 diabetes (T2D), and cardiovascular disease, and may facilitate improvements in age-related reduction in quality of life^2,3^. The World Health Organisation (WHO) estimates that 31% of adults are below recommended physical activity levels^4^, and that physical inactivity is the 4th leading cause of mortality globally. Physical inactivity increases the risk of pathologies, including cardiovascular disease, cancer, and T2D. Reciprocally, diseases such as T2D are strongly associated with immobility and are a risk factor for mobility disability, frailty and decreased independence^5^. How exercise exerts its systemic adaptive effects remains poorly understood. Elucidating the fundamental molecular mechanisms and intra- and inter-organ signals through which exercise mediates systemic adaptations, driving beneficial effects, holds potential for the identification of both therapeutic targets and new strategies to treat a range of diseases, including T2D.

The repeated contraction, coordination and transfer of force by skeletal muscle is central to physical activity. Skeletal muscle acts as both a source and target of the systemic signals which contribute to the adaptive remodelling and beneficial effects of exercise^6^. The transcriptional coactivator peroxisome proliferator-activated receptor-gamma coactivator-1α (PGC-1α) controls the expression of metabolic genes within skeletal muscle and is a key regulator of the skeletal muscle adaptive response to exercise^7,8^. Mice with muscle-specific PGC-1α expression exhibit enhanced endurance exercise performance^9^. Exercise training enhances expression of PGC-1α in skeletal muscle, which stimulates mitochondrial biogenesis, fatty acid β- oxidation, glucose transport, as well as an induction of muscular fiber-type remodelling from glycolytic fast-twitch type IIX muscle fibers to intermediate type IIA and oxidative type I slow-twitch muscle fibers^10–12^. These adaptations in muscle physiology contribute to improved aerobic and endurance exercise performance^13–16^.

The production and secretion of exercise-responsive myokines, muscle-derived endocrine signals, contributes to interorgan coordination and the systemic adaptation to exercise^17,18^. We demonstrated that exercise training-induced PGC-1α expression in skeletal muscle drives the biosynthesis and secretion of the non-protein β-amino acid, β-aminoisobutyric acid (BAIBA)^19^. BAIBA functions as an exercise and PGC-1α regulated myokine-like metabokine, which induces hepatic β-oxidation and subcutaneous adipose tissue browning through PPARα, with subsequent protective effects against markers of cardiometabolic disease^19^. BAIBA is also a bone-protective factor that prevents osteocyte cell death^20,21^ and reduces insulin resistance and inflammation^22^. However, the contribution of BAIBA to exercise-mediated skeletal muscle adaptation and exercise performance is not understood.

Here, we demonstrate that the L-enantiomer of BAIBA has autocrine and paracrine signalling effects in skeletal muscle which are required for exercise-mediated adaptive responses, including fiber-type remodelling, increased mitochondrial number and function, muscle hypertrophy and myotube differentiation. We suggest that exogenous BAIBA treatment acts as an exercise mimetic, improving exercise performance with therapeutic potential to treat skeletal muscle dysfunction in a diet-induced model of T2D.

## Results

### Enantiomeric D/L-BAIBA enhances voluntary exercise performance and skeletal muscle function in vivo

**Fig. 1:**
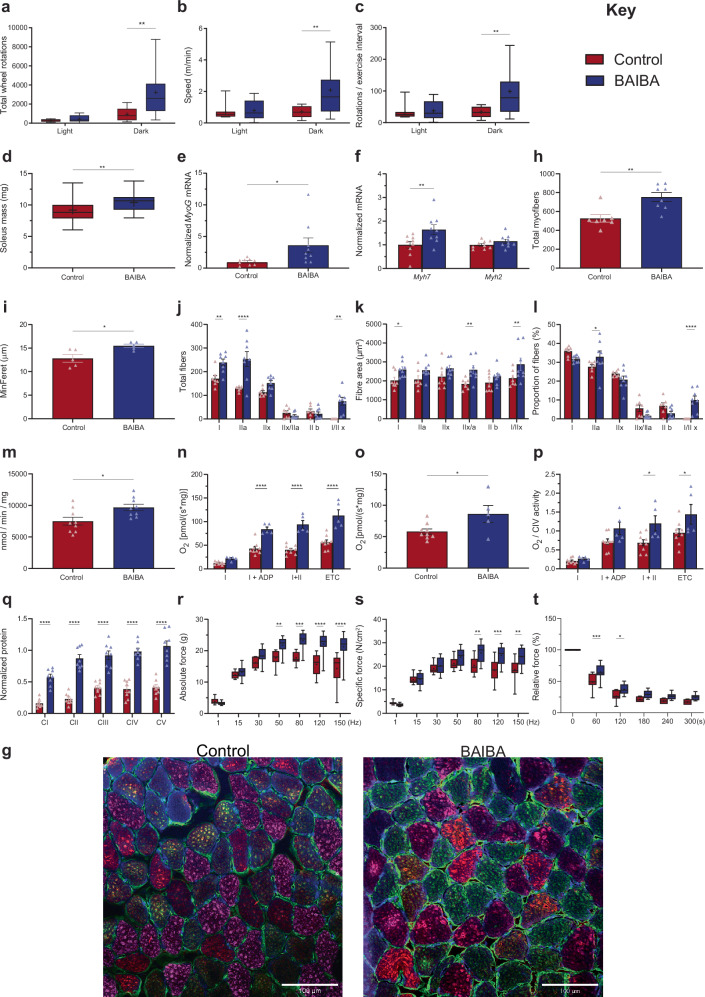
BAIBA induces an exercise training-like effect on exercise performance and skeletal muscle function in vivo. **a** Total running wheel rotations (24 hr diurnal light and dark phases) in mice receiving BAIBA compared to untreated controls (Control *n* = 10, BAIBA *n* = 11; Dark, *p* = 0.0041; two-way ANOVA). **b** Average running speed (m/min) (24 hr diurnal light and dark phases) of mice receiving BAIBA compared to untreated controls (Control *n* = 10, BAIBA *n* = 11; *p* = 0.01; two-way ANOVA). **c** Running wheel rotation per exercise interval (24 hr diurnal light and dark phases) of mice receiving BAIBA compared to untreated controls (Control *n* = 10, BAIBA *n *= 11; *p* = 0.01; two-way ANOVA). **d** Soleus muscle mass of mice receiving BAIBA compared to untreated controls (Control *n* = 32, BAIBA *n* = 29; *p* = 0.004; two-tailed t-test). **e** Myogenin (*Myog*) mRNA expression in the soleus muscle from mice receiving BAIBA compared to untreated controls (Control *n* = 9; BAIBA *n* = 9; *p* = 0.04; two-tailed t-test) (**f**) Myosin heavy chain 7 (*Myh7*) and *Myh2* mRNA expression in the soleus from mice receiving BAIBA compared to untreated controls (Control *n* = 9; BAIBA *n* = 9; *Myh7 p* = 0.02; two-tailed t-test). **g** Cross sectional confocal images of soleus from mice receiving BAIBA (100 mg/Kg/day) compared to untreated controls; myosin heavy chain I (MyHCI; purple), MyHCIIa (green), MyHCIIx (red), nuclei (Hoescht; blue) and basal lamina (laminin; green). Scale bar = 100 μm. **h** Total myofibers in soleus from mice receiving BAIBA compared to untreated controls (Control *n* = 7 BAIBA *n* = 8; *p* = 0.003; two-tailed t-test). **i** MinFeret (μm) diameter for soleus fibers from mice receiving BAIBA compared to untreated controls (Control *n* = 5 BAIBA *n* = 5); *p* = 0.02; two-tailed t-test). **j** Total myofibers by fibertype (I, IIa, IIx, IIb and hybrid IIx/a and I/IIx) of soleus from mice receiving BAIBA compared to untreated controls (Control *n* = 7 BAIBA *n* = 8; I *p* = 0.004, IIa *p* = 0.00000005, I/IIx *p* = 0.001; Two-way ANOVA). **k** Myofiber cross sectional area by fibertype (I, IIa, IIx, IIb and hybrid IIx/a and I/IIx) of soleus from mice receiving BAIBA compared to untreated controls (Control *n* = 7 BAIBA *n* = 8; I *p* = 0.04; IIx/a *p* = 0.008; I/IIx *p* = 0.01; Two-way ANOVA). **l** Proportion of myofibers by fibertype (%) (I, IIa, IIx, IIb and hybrid IIx/a and I/IIb) of soleus from mice receiving BAIBA (100 mg/Kg/day) compared to untreated controls (Control *n* = 7 BAIBA *n* = 8; IIa *p* = 0.019, I/IIx *p* = 0.00002: Two-way ANOVA). **m** Citrate synthase activity (nmol/min/mg) in the soleus of mice receiving BAIBA compared to untreated controls (Control *n* = 9 BAIBA *n* = 9; *p* = 0.016; two-tailed t-test). **n** High-resolution respirometry analysis of soleus from mice receiving BAIBA compared to untreated controls assessed for complex I (I), complex I-supported oxidative phosphorylation (I + ADP), complex I & II-supported oxidative phosphorylation (I + II) and maximal uncoupled substrate oxidation (ETC), corrected for tissue mass (control = 9, BAIBA = 5; I + ADP *p* = 0.000006, I + II *p* = 0.00000001, ETC *p* = 0.000000004; two-way ANOVA). **o** High-resolution respirometry assay for complex IV (CIV) activity in soleus from mice receiving BAIBA compared to untreated controls (control = 9, BAIBA = 5; *p* = 0.029; two-tailed t-test). **p** High-resolution respirometry analysis of soleus from mice receiving BAIBA compared to untreated controls assessed for I, I + ADP, I + II and ETC, corrected for CIV activity (control = 9, BAIBA = 5; I + II *p* = 0.015, ETC *p* = 0.023; two-way ANOVA). **q** Quantitation of immunoblot for mitochondrial respiratory complex proteins I – V from the soleus of mice receiving BAIBA compared to untreated controls (*n* = 8; CI *p* = 0.000002, CII *p* = 0.000000000004, CIII *p* = 0.000000006, CIV *p* = 0.00000000005, CV *p* = 0.000000000002; Two-way ANOVA). **r** Absolute ex vivo contractile force under increasing stimuli (Hz) of the soleus from mice receiving BAIBA compared to untreated controls (control = 10, BAIBA = 12; 50 Hz *p* = 0.0071; 80 Hz *p* = 0.0001; 120 Hz *p* = 0.000004, 150 Hz *p* = 0.0000007; two-way ANOVA). **s** Specific ex vivo contractile force (N/cm^2^) under increasing stimuli (Hz) of the soleus from mice receiving BAIBA compared to untreated controls (control = 10, BAIBA = 12; 80 Hz *p* = 0.0034; 120 Hz *p* = 0.0002, 150 Hz *p* = 0.004; two-way ANOVA). **t** Relative specific force over 300 s in the soleus of mice receiving BAIBA compared to untreated controls (control = 7, BAIBA = 10; 60 s *p* = 0.0003; 120 s *p* = 0.02; Two-way ANOVA). Data in bar charts are mean ± SEM with data points shown. Box and whisker plots show 25th to 75th percentile (box) min to max (whiskers), mean (+) and median (−). Control = red, BAIBA-treated = blue. * *p* ≤ 0.05, ** *p* < 0.01, *** *p* < 0.001, **** *p* < 0.0001. Source data are provided as a Source Data file.

Six week old male C57BL6/J mice were treated with 100 mg/kg/day BAIBA in drinking water for 6 weeks using our protocol^19^ (Supplementary Fig. 1a). Plasma BAIBA concentrations increased within the physiological exercise-induced low micromolar range (Supplementary Fig. 1b)^19^. BAIBA treatment reduced weight gain and fat mass (Supplementary Fig. 1c and 1d). Indirect calorimetry indicated enhanced oxygen consumption and energy expenditure in BAIBA-treated mice (Supplementary Fig. 1e and 1f). Basal activity and food intake were unaffected (Supplementary Fig. 1g–i). We used free wheel running analysis, preceded by a 3-day habituation period, to examine voluntary exercise performance in these mice. BAIBA treated mice exhibited significantly greater total wheel rotations (Fig. 1a), greater running speed (Fig. 1b) and greater number of rotations per exercise interval (Fig. 1c). However, BAIBA treatment does not affect the total number of exercise bouts the animals perform (Supplementary Fig. 1j) or the total average duration of all exercise bouts (Supplementary Fig. 1k). BAIBA enhances voluntary exercise performance in mice without affecting behavioural measures indicative of volition, such as the number or duration of exercise bouts.

Next, we determined whether BAIBA induced adaptations in skeletal muscle morphological, metabolic and functional phenotype in the mice. BAIBA enhanced soleus (Fig. 1d), tibialis anterior (TA) and gastrocnemius (Supplementary Fig. 1l) muscle mass. Exploring potential mediators of muscle mass, BAIBA-treatment did not affect the expression of atrophy related genes including E3 ubiquitin-protein ligase *Trim63* and Atrogin 1 (*Fbxo32*) (Supplementary Fig. 1m). However, BAIBA increased expression of the myogenic regulator myogenin (*Myog*) in soleus (Fig. 1e). Muscle fiber type composition influences aerobic exercise capacity^23^. Endurance exercise performance and fatigue resistance are associated with a higher proportion of type I oxidative slow-twitch fibers relative to type II glycolytic fast twitch fibers in locomotor muscles^23^. Therefore, we examined the predominant oxidative soleus muscle expression of myosin heavy chain 2 (*Myh2*) and *Myh7*, encoding the major myosin heavy chain isoforms expressed in oxidative type IIa fibers and oxidative type I fibers, respectively (Fig. 1f). BAIBA enhanced the expression of *Myh7*. We used immunofluorescence to determine the effect of BAIBA on soleus muscle fiber-type composition (Fig. 1g–l). BAIBA increased the total number of fibers (Fig. 1h), and their diameter (Fig. 1i), with significantly increased numbers of fatigue resistant type I, type IIa and hybrid type I/IIx fibers (Fig. 1j). BAIBA specifically increased the area of type I and hybrid type I/IIa and I/IIx fibers (Fig. 1k). Proportionally, BAIBA increased the type IIa and type I/IIx fibers with a non-significant reduction in type IIb and hybrid type IIx/IIa fibers (Fig. 1l). We then applied our immunofluorescence approach to determine the effect of BAIBA on the mixed fiber composition of the gastrocnemius muscle (Supplementary Fig. 1n). BAIBA did not significantly increase the total myofiber number in gastrocnemius (Supplementary Fig. 1o). However, BAIBA induced fiber remodelling, significantly increasing the number of fatigue resistant type IIa fibers in gastrocnemius (Supplementary Fig. 1p). BAIBA also significantly increased myofiber diameter (Supplementary Fig. 1q) in gastrocnemius with a specific increase in the diameter of type I/IIx hybrid fibers (Supplementary Fig. 1r).

PGC1α drives skeletal muscle mitochondrial biogenesis^24^. Muscle mitochondrial content is a key contributor to systemic exercise capacity^25^. Citrate synthase activity, a marker of mitochondrial density and TCA cycle flux, was higher in the soleus (Fig. 1m), EDL and gastrocnemius (Supplementary Fig. 1s) of BAIBA-treated mice compared with controls. High-resolution respirometry determined whether the greater mitochondrial content of muscle from BAIBA-treated mice facilitates increased mitochondrial respiratory capacity. Soleus mitochondrial electron transport chain (ETC) coupled complex I-mediated oxidative phosphorylation (stimulated with pyruvate, malate, glutamate and ADP) was enhanced in BAIBA-treated mice (Fig. 1n). Muscle complex I and II-mediated succinate-stimulated respiration was higher in soleus muscle from BAIBA-treated mice (Fig. 1n). The ETC was chemically uncoupled using CCCP to determine maximal respiration, which was significantly increased in soleus from BAIBA-treated mice (Fig. 1n). A secondary assessment of mitochondrial content, complex IV respiratory activity^26^, supported enhanced BAIBA-induced mitochondrial content in the soleus (Fig. 1o). Correction of high-resolution respirometry data for mitochondrial content (complex IV activity) indicated that BAIBA-stimulated enhanced respiratory capacity remained even after normalization for differences in mitochondrial content (Fig. 1p). Using immunoblotting we identify that BAIBA treatment increased the protein levels of all mitochondrial electron transport respiratory complexes (complex I–V) in soleus (Fig. 1q; Supplementary Fig. 1t) as well as EDL and gastrocnemius muscles (Supplementary Fig. 1t). BAIBA enhances both mitochondrial content and intrinsic respiratory capacity in skeletal muscle.

We assessed whether the increase in muscle mass, mitochondrial number, mitochondrial respiratory capacity and number of type I, IIa and I/IIx hybrid muscle fibers in BAIBA-treated mice resulted in improved muscle contractile function. Isolated soleus was attached to a force transducer in an organ bath, stimulated with a supramaximal current and contractile properties were assessed in vitro using established force-frequency and fatigue protocols^27^. Soleus force-frequency was greater in BAIBA-treated mice for both absolute and normalized contractile force (Fig. 1r, s). BAIBA-treatment also increased soleus fatigue resistance, independent of blood flow or neural input (Fig. 1t). As BAIBA increased both mitochondrial respiratory complex expression and citrate synthase activity in EDL, we investigated whether the contractile effects on soleus translated to EDL given its greater proportion of type II fibers. EDL was examined with the contractile fatigue protocols. Consistently BAIBA also increased EDL fatigue resistance (Supplementary Fig. 1u).

Next, we examined whether the BAIBA-induced effects exhibit sexual dimorphism. As for male mice, six week old female C57BL6/J mice were treated with 100 mg/kg/day BAIBA in drinking water for 6 weeks. BAIBA treated female mice exhibited significantly greater total free wheel running rotations (Supplementary Fig. 2a), soleus and TA muscle mass (Supplementary Fig. 2b), and soleus *Myog, Myh7* and *Myh2* expression (Supplementary Fig. 2c). Examination of the mitochondrial phenotype using high-resolution respirometry in soleus from BAIBA-treated female mice indicated increased mitochondrial capacity (Supplementary Fig. 2d) and content (complex IV respiratory activity) (Supplementary Fig. 2e). Soleus force-frequency was also greater in BAIBA-treated female mice for both absolute and normalized contractile force (Supplementary Fig. 2f, g). BAIBA-treatment also increased soleus fatigue resistance in female mice (Supplementary Fig. 2h). The effects of BAIBA on female mice phenocopy the effects in male mice and do not display sexual dimorphism.

BAIBA-induced improvements in intrinsic muscle function may contribute to improved voluntary exercise performance in mice across both sexes. These findings suggest that BAIBA regulates muscle fatigue resistance, consistent with effects on exercise tolerance, through muscle mitochondrial content and respiratory capacity.

### BAIBA enhances the effects of exercise training to improve muscle function and exercise performance in mice

**Fig. 2:**
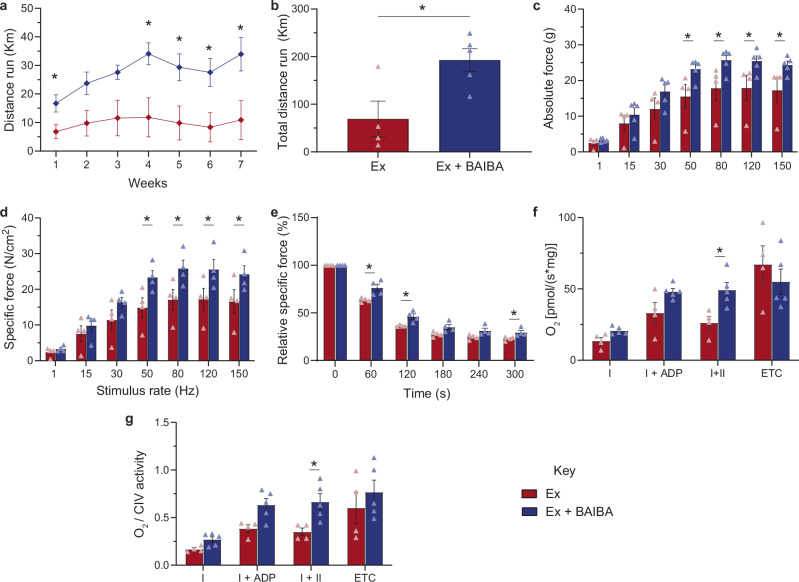
BAIBA enhances the effects of exercise training. **a** Weekly distance run by mice receiving BAIBA compared to untreated controls (Exercise [Ex] *n* = 4, Ex + BAIBA *n* = 5; week 4 *p* = 0.016; week 5 *p* = 0.046; week 6 *p* = 0.05; week 7 *p* = 0.01; Two-way ANOVA). **b** Total distance run (Km) of mice receiving BAIBA compared to untreated controls over 6 weeks (Ex *n* = 4, Ex + BAIBA *n* = 5; *p* = 0.02; two-tailed t-test). **c** Soleus absolute ex vivo contractile force under increasing stimuli (Hz) from mice with access to running wheels and receiving BAIBA compared to untreated controls (Ex *n* = 4, Ex + BAIBA *n* = 5; 50 Hz *p* = 0.02; 80 Hz *p* = 0.019; 120 Hz *p* = 0.02; 150 Hz *p* = 0.03; Two-way ANOVA). **d** Soleus specific ex vivo contractile force under increasing stimuli (Hz) from mice with access to running wheels and receiving BAIBA compared to untreated controls (Ex *n* = 4, Ex + BAIBA *n* = 5; 50 Hz *p* = 0.013; 80 Hz *p* = 0.012; 120 Hz *p* = 0.015; 150 Hz *p* = 0.026; Two-way ANOVA). **e** Soleus relative specific force over 300 s in the soleus of mice with access to running wheels and receiving BAIBA compared to untreated controls (Ex *n* = 4, Ex + BAIBA *n* = 4; 60 s *p* = 0.038; 120 s *p* = 0.038; 300 s *p* = 0.043; Two-way ANOVA). **f** High-resolution respirometry analysis of soleus from mice with access to running wheels receiving BAIBA compared to untreated controls assessed for complex I (I), complex I-supported oxidative phosphorylation (I + ADP), complex I & II-supported oxidative phosphorylation (I + II) and maximal uncoupled substrate oxidation (ETC), corrected for tissue mass (Ex *n* = 4, Ex + BAIBA *n* = 5; I + II *p* = 0.017; Two-way ANOVA. **f** High-resolution respirometry analysis of soleus from mice with access to running wheels receiving BAIBA compared to untreated controls assessed for I, I + ADP, I + II and ETC, corrected for CIV activity (Ex *n* = 4, Ex + BAIBA *n* = 5; I + II *p* = 0.016; Two-way ANOVA). Data in bar charts are mean ± SEM with data points shown. Exercise = red. Exercise + BAIBA = blue. * *p* ≤ 0.05. Source data are provided as a Source Data file.

To determine whether BAIBA conveys additive effects in the adaptation to exercise, a distinct group of C57BL6/J mice underwent 6 weeks of free wheel running either with or without 100 mg/kg/day BAIBA treatment. BAIBA-treatment increased plasma BAIBA concentration within the physiological range (Supplementary Fig. 3). BAIBA increased the distance run by the mice in addition to the exercise conditioning (Fig. 2a, b). Isolated soleus from the exercised and BAIBA-treated mice was analysed for contractile properties via force-frequency protocols^27^. Soleus absolute and specific forces were greater in BAIBA-treated and exercised mice than mice undergoing exercise alone (Fig. 2c, d). The soleus fatigue resistance in BAIBA-treated and exercised mice was also greater than in the exercise only group (Fig. 2e). Examining the soleus mitochondrial phenotype using high-resolution respirometry indicated that BAIBA enhanced mitochondrial complex I and II-mediated oxidative phosphorylation coupled respiration (Fig. 2f) and this effect was maintained following correction for mitochondrial number (Fig. 2g). BAIBA enhances the effect of exercise training on muscle mitochondrial respiration and contractile function.

### BAIBA induces a molecular signature of muscle adaptation and differentiation in vivo

**Fig. 3:**
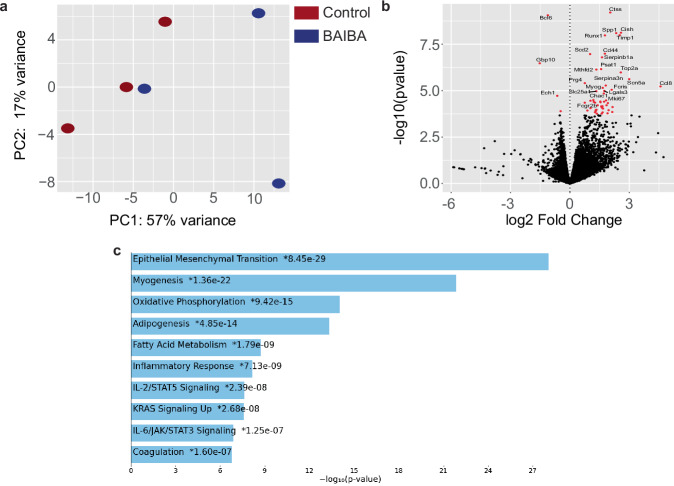
BAIBA induces a molecular signature of muscle adaptation and differentiation in vivo. **a** Principal component analysis plot showing separation of RNAseq data obtained from the soleus of control (red) and BAIBA-treated (blue) mice (*n* = 3). **b** Volcano plot of differentially expressed genes identified from RNAseq data from the soleus of control (red) and BAIBA-treated (blue) mice (*n* = 3; fold-change cut-off = 1.2; adjusted *p*-value cut-off = 0.05; Wald test with Benjamini-Hochberg). **c** Gene Set Enrichment Analysis of the RNAseq data obtained from the soleus of control and BAIBA-treated mice (*n* = 3) (Fisher exact test adjusted for false discovery rate). Source data are deposited at (https://www.ebi.ac.uk/arrayexpress/experiments/E-MTAB-14747).

We explored underlying mechanisms by performing RNAseq in soleus muscle samples from 6-week BAIBA-treated mice compared with controls (https://www.ebi.ac.uk/arrayexpress/experiments/E-MTAB-14747). We found separation between the control and BAIBA-treated groups using Principal Component Analysis (Fig. 3a). Our analysis identified 54 unique and significantly differentially expressed genes (DEGs) between BAIBA-treated and control mice, with 50 DEGs up- and four DEGs down-regulated (Fold-change cut-off = 1.2, adjusted *p*  <  0.05; Fig. 3b). Enrichment analysis of the RNAseq data (using ClusterProfileR v4.14.4) indicated Gene Ontology terms differentially regulated by BAIBA including tissue remodelling (adjusted *p* value = 4.2e^–5^; *Ctss*/*Timp1*/*Spp1*/*Ccr2*/*Csf1r*), response to muscle inactivity involved in regulation of muscle adaptation (adjusted *p* value = 0.001; *Scn5a*/*Myog*), muscle atrophy (adjusted *p* value = 0.002; *Myog*/*Gatm*), negative regulation of proteolysis (adjusted *p* value = 0.003; *Timp1*/*Cd44*/*Serpinb1a*/*Serpina3n*/*Chac1*), regulation of myoblast fusion (adjusted *p* value = 0.004; *Ccl8*, *Myog*) and muscle cell differentiation (adjusted *p*-value = 0.006; *Ccl8*/*Myog*/*Csf1r*/*Csrp2*/*Cfd*). Performing Gene Set Enrichment Analysis (GSEA)^28^ of the significantly differentially expressed genes searching against the MSigDB Molecular Signatures Database^29^ identifies that BAIBA induces transcriptional processes consistent with our observations and indicative of skeletal muscle structural and metabolic remodelling including mesenchymal remodelling (adjusted *p* value 8.45e^−29^), myogenesis (adjusted *p* value 8.45e^−29^), oxidative phosphorylation (adjusted *p* value 9.42e^−15^) and fatty acid metabolism (adjusted *p* value 1.79e^−9^) (Fig. 3c). Using ShinyGO gene ontology^30^ to search against the KEGG database to better understand the regulatory processes governing the observed changes in metabolism identified similar changes in mitochondrial metabolism (TCA cycle), fatty acid metabolism and ECM remodelling, but interestingly highlighted the regulation of PPAR signalling (Supplementary Fig. 4). These data suggest BAIBA initiates a molecular programme of muscle differentiation and metabolic remodelling overlapping with aspects of exercise training.

### BAIBA enhances human primary skeletal myocyte proliferation and differentiation

**Fig. 4:**
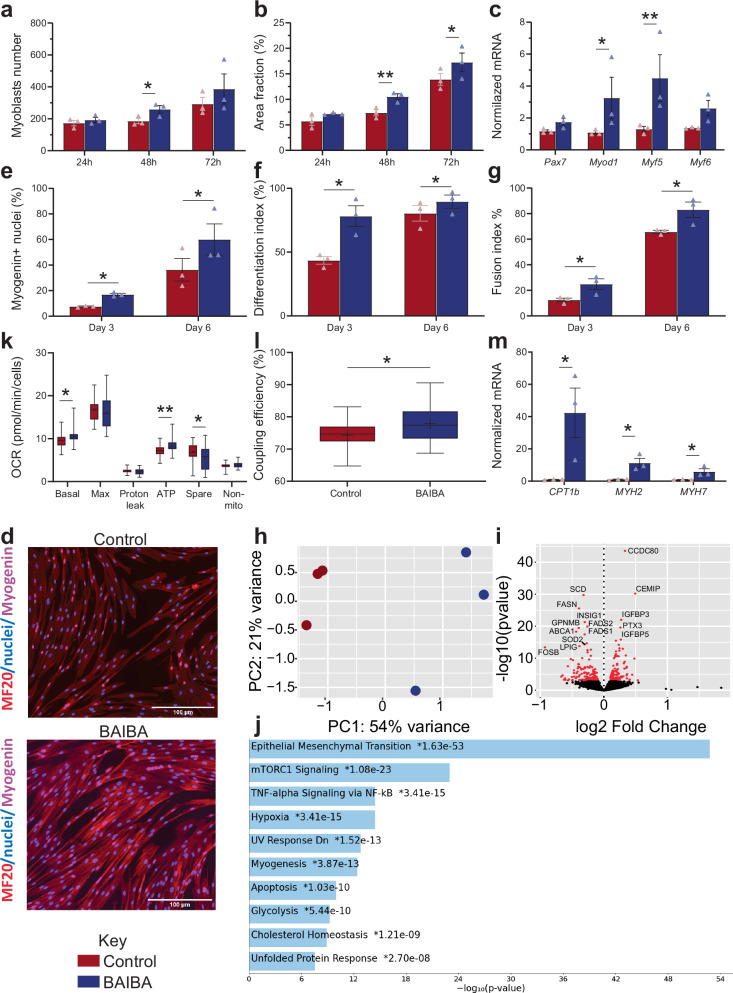
BAIBA induces differentiation and exercise-like adaptation in human primary skeletal myocytes. **a** Myoblast number when treated with BAIBA (10 μM) at 24 h, 48 h and 72 h post seeding (*n* = 3; 48 h control vs BAIBA *p* = 0.047; paired two-tailed Student’s t-test) (**b**) Confluence (area fraction %) at 24 h, 48 h and 72 h post-seeding of human skeletal muscle cells (HSkMCs) treated with BAIBA (10 μM) (*n* = 3; 48 h control vs BAIBA *p* = 0.003, 72 h control vs BAIBA *p* = 0.04; paired two-tailed Student’s t-test). **c** Expression of paired box protein (*PAX-7*), myoblast determination protein 1 (*MYOD1*), myogenic factor 5 (*MYF5*), *MYF6* in HSkMCs treated with BAIBA (10 μM) for 72 h post-seeding (*n* = 3; *MYOD1* Control vs BAIBA *p* = 0.049, *MYF5* Control vs BAIBA *p* = 0.006; two-tailed Student’s t-test). **d** Representative confocal images of human myotubes treated with BAIBA (10 μM) and stained for myosin heavy chain 2 (MF20; red), nuclei (blue) and myogenin (magenta) (Scale bar = 100μm). **e** Myogenin positive nuclei (%) in BAIBA (10 μM) treated myoblasts at day 3 and day 6 of differentiation to myotubes (*n* = 3; day 3 *p* = 0.01, day 6 *p* = 0.03; paired two-tailed Student’s t-test). **f** The differentiation index in BAIBA (10 μM) treated myoblasts at day 3 and day 6 of differentiation to myotubes (*n* = 3; day 3 *p* = 0.013, day 6 *p* = 0.03; paired two-tailed Student’s t-test). **g** The fusion index in BAIBA (10 μM) treated myoblasts at day 3 and day 6 of differentiation to myotubes (*n* = 3; day 3 *p* = 0.049, day 6 *p* = 0.047; paired two-tailed Student’s t-test). **h** Principal component analysis showing separation of RNAseq data obtained from control (red) and BAIBA (10 μM; blue) treated myotubes (*n* = 3). **i** Volcano plot of differentially expressed genes identified from RNAseq data obtained from control and BAIBA (10 μM) treated myotubes (*n* = 3; fold-change cut-off = 1.2; adjusted *p* value cut-off = 0.05; Wald test with Benjamini-Hochberg). **j** Gene Set Enrichment Analysis of the RNAseq data obtained from control and BAIBA (10 μM) treated myotubes (Fisher exact test adjusted for false discovery rate). **k** Oxygen consumption rate of human myotubes treated with BAIBA (10 μM) showing basal, maximal (max), proton leak (leak), ATP production (ATP), spare respiratory capacity (spare) and non-mitochondrial respiration (non-mito) (control *n* = 25, BAIBA *n* = 24; Basal *p* = 0.039, ATP production *p* = 0.008, Spare respiratory capacity *p* = 0.03; Two-way ANOVA). **l** Respiratory coupling efficiency of human myotubes treated with BAIBA (10 μM) (*n* = 25, BAIBA *n* = 24, *p* = 0.016; two-tailed Student’s t-test). **m** Expression of myosin heavy chain 7 (*MYH7*), carnitine palmitoyl transferase 1b (*CPT1b*) and *MYH2* in myoblasts treated with BAIBA (10 μM) and differentiated to myotubes (*n* = 3; *CPT1b p* = 0.05; *MYH2 p* = 0.023; *MYH7 p* = 0.049; two-tailed Student’s t-test). Data in bar charts are mean ± SEM with data points shown. Control = red, BAIBA = blue. Box and whisker plots show 25th to 75th percentile (box) min to max (whiskers), mean (+) and median (−). * *p* ≤ 0.05, ** *p* < 0.01, *** *p* < 0.001, **** *p* < 0.0001. *n* = number of biological replicates as independent culture plates. Source data are provided as a Source Data file.

Given BAIBA initiates a myogenic transcriptional programme in vivo, we investigated whether BAIBA regulates myogenesis in a translational myocyte model. Primary human skeletal muscle cells (HSkMCs) were seeded and immediately treated with a physiological 10 μM BAIBA concentration^19^. Proliferation was assessed in the cells 24 h, 48 h and 72 h post-seeding. BAIBA treatment enhanced myoblast proliferation in vitro, assessed by both cell number and area (Fig. 4a, b). BAIBA also increased expression of key regulatory genes for skeletal muscle differentiation 72 h post-seeding including myoblast determination protein 1 (*MYOD1*), and myogenic factor 5 (*MYF5*) (Fig. 4c). Next, we treated myoblasts with 10 μM BAIBA during a six day in vitro differentiation period. For a direct assessment of differentiation and myoblast fusion we used immunohistochemistry to stain the BAIBA treated and differentiated myotubes for muscle myosin (MF20), nuclei (Hoechst) and the marker of differentiation, myogenin (Fig. 4d). BAIBA significantly increased the percentage of myogenin positive cells (Fig. 4e), the differentiation index (Fig. 4f), and fusion index (Fig. 4g) at both day 3 and day 6 of differentiation. Therefore, BAIBA directly increases the differentiation of human myotubes in vitro.

We then more broadly investigated the molecular phenotype induced by BAIBA in human skeletal myocytes by performing RNAseq in 10 μM BAIBA-treated HSKMCs compared with controls (Fig. 4h) (https://www.ebi.ac.uk/arrayexpress/experiments/E-MTAB-14746). Our analysis identified 267 unique and significantly DEGs between BAIBA-treated and control myocytes, with 115 DEGs up- and 152 DEGs down-regulated (adjusted *p*  <  0.05; Fig. 4i). Gene ontology analysis of the RNAseq data indicated terms differentially regulated by BAIBA included muscle organ development (adjusted *p* value = 1.1e^−7^; *COL3A1*/*EDNRA*/*RYR1*/*FOS*/*EGR1*/*TAGLN*/*TPM1*/*LOX*/*MYLK*/*ELN*/*SPEG*/*ARID5B*/*NEB*/*SMTN*/*LAMA5*/*COL11A1*/*FZD2*/*NUPR1*/*RCAN1*/*NIBAN2*/*WNT5A*/*MYMX*/*ENG*/*PLEC*), muscle cell differentiation (adjusted *p* value = 1.26e^−6^; *IGFBP5*/*MMP14*/*EDNRA*/*RYR1*/*LMNA*/*EREG*/*LAMA1*/*TPM1*/*LOX*/*COMP*/*SPEG*/*SORT1*/*FZD7*/*CDH2*/*NEB*/*LAMC1*/*NPNT*/*DMPK*/*G6PD*/*RCAN1*/*NIBAN2*/*MYMX*/*ENG*/*PLEC*), response to mechanical stimulus (adjusted *p* value = 4.271.1e^−5^; *FOSB*/*TXNIP*/*MMP14*/*FOS*/*MDK*/*COL1A1*/*CHI3L1*/*THBS1*/*CDH2*/*BNIP3*/*COL11A1*/*JUN*/*ENG*/*PLEC*/*MMP2*), muscle organ morphogenesis (adjusted *p* value = 0.0001; *COL3A1*/*EDNRA*/*TPM1*/*MYLK*/*ARID5B*/*COL11A1*/*FZD2*/*WNT5A*/*ENG*), muscle cell proliferation (adjusted *p* value = 0.0004; *IGFBP3*/*IGFBP5*/*EREG*/*FOS*/*HMOX1*/*THBS1*/*TPM1*/*ELN*/*APOE*/*JUN*/*IRAK1*/*ADAMTS1*/C*DKN1A*/*MMP2*), muscle cell migration (adjusted *p* value = 0.0004; *IGFBP3*/*IGFBP5*/*SERPINE1*/*MDK*/*LRP1/NRP1*/*TPM1*/*ADAMTS1*/*PLAU*), response to muscle stretch (adjusted *p* value = 0.03; *FOS*/*CDH2*/*JUN*) and PPAR signalling (adjusted *p* value = 0.02; *FADS2*/*SCD*/*FABP3*). Performing Gene Set Enrichment Analysis (GSEA)^28^ of the significantly differentially expressed genes in BAIBA treated human skeletal myotubes, again using the MSigDB Molecular Signatures Database^29^, identifies that BAIBA induces transcriptional processes consistent with our observations in vivo and indicative of skeletal muscle structural remodelling including mesenchymal remodelling (adjusted *p* value 1.63e^−53^), and myogenesis (adjusted *p* value 3.87e^−13^) (Fig. 4j).

### BAIBA induces exercise-like metabolic adaptation in human primary skeletal myocytes

Endurance exercise training increases muscle mitochondrial function^31,32^ and remodels fibers towards a more oxidative phenotype^33^. Therefore, we examined the mitochondrial respiration of differentiated myotubes treated with BAIBA (10 μM) using a Seahorse bioanalyzer. BAIBA increased basal oxygen consumption and ATP production, and decreased spare respiratory capacity in human myotubes (Fig. 4k). BAIBA also increased myotube coupling efficiency (Fig. 4l). BAIBA increased the expression of markers of terminal differentiation towards oxidative muscle fibers in the human myotubes including *MYH7*, carnitine palmitoyl transferase 1b (*CPT1b*) and *MYH2* (Fig. 4m). BAIBA increases oxidative metabolism and mitochondrial function in human myotubes in vitro.

### The effect of BAIBA on markers of muscle regeneration in a mouse skeletal muscle injury model

BAIBA initiated a myogenic programme in skeletal muscle in vivo and induced proliferation and differentiation in human myoblasts. These may be hallmarks of muscle regeneration^34^. Therefore, we investigated the effect of BAIBA on muscle regeneration in a mouse injury model. C57BL6 mice were either treated with 100 mg/kg/day BAIBA in drinking water for 1 week, or remained untreated. The mice were injected contra-laterally with cardiotoxin (50 µL, 10 µM CTX) into TA muscle of the left leg leaving the right leg uninjured. BAIBA-treated mice then continued with the 100 mg/kg/day BAIBA treatment regimen for a further 2 weeks^19^. Although TA mass was increased to a greater extent in injured limbs compared with uninjured limbs of BAIBA-treated mice (Supplementary Fig. 5a), the percentage of centralized nuclei in the TA of the injured limb did not differ between BAIBA-treated and control mice (Supplementary Fig. 5b).

### BAIBA is therapeutic in a diet-induced obesity and T2D model of skeletal muscle dysfunction

**Fig. 5:**
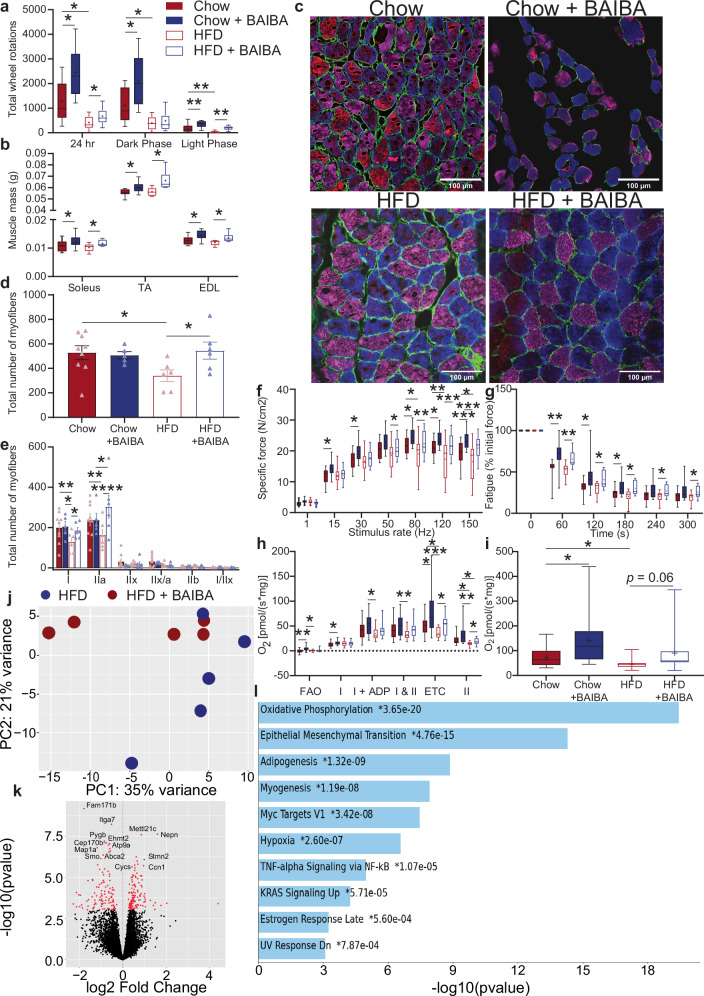
BAIBA is therapeutic in an in vivo model of diabetes and skeletal muscle dysfunction. **a** Total running wheel rotations (diurnal 24 hr light and dark phases) in chow or high fat diet (HFD)-fed receiving BAIBA compared to untreated controls (Chow *n* = 8, Chow + BAIBA *n* = 10, HFD *n* = 14, HFD + BAIBA *n* = 12; 24 hr Chow vs Chow + BAIBA *p* = 0.017, Chow vs HFD *p* = 0.021, HFD vs HFD + BAIBA *p* = 0.046; Dark phase Chow vs Chow + BAIBA *p* = 0.028, Chow vs HFD *p* = 0.024; Light phase Chow vs Chow + BAIBA *p* = 0.005, Chow vs HFD *p* = 0.006, HFD vs HFD + BAIBA *p* = 0.001; One-way ANOVA) (**b**). Soleus, tibialis anterior (TA) and extensor digitorum longus (EDL) mass in chow or HFD-fed mice receiving BAIBA compared to untreated controls (chow soleus *n* = 17, TA *n* = 7, EDL *n* = 9; chow + BAIBA soleus *n* = 17, TA *n* = 12, EDL *n* = 10, HFD soleus *n* = 10, TA *n* = 6, EDL *n* = 8; HFD + BAIBA soleus *n* = 10, TA *n* = 6, EDL *n* = 8; Soleus chow vs chow + BAIBA *p* = 0.029, HFD vs HFD + BAIBA 0.024; TA chow vs chow + BAIBA *p* = 0.018, HFD vs HFD + BAIBA *p* = 0.039; EDL chow vs chow + BAIBA *p* = 0.02, HFD vs HFD + BAIBA *p* = 0.023; One-way ANOVA). *n* = individual mouse. **c** Composite cross sectional confocal soleus images of chow or HFD-fed mice receiving BAIBA compared to untreated controls; myosin heavy chain I (MyHCI; purple), MyHCIIa (blue), MyHCIIx (red), nuclei (Hoescht; blue), basal lamina (laminin; green). Scale bar = 100 μm. **d** Total soleus myofibers of chow or HFD-fed mice receiving BAIBA (100 mg/Kg/day) compared to untreated controls (chow *n* = 9, chow + BAIBA *n* = 5, HFD *n* = 6, HFD + BAIBA *n* = 6; chow vs HFD *p* = 0.02, HFD vs HFD + BAIBA *p* = 0.02; One-way ANOVA). **e** Total soleus myofibers by fibertype (I, IIa, IIx, IIb and hybrid IIx/a and I/IIb) of chow or HFD-fed mice receiving BAIBA compared to untreated controls (chow *n* = 9, chow + BAIBA *n* = 5, HFD *n* = 6, HFD + BAIBA *n* = 6; Type I chow vs HFD *p* = 0.006, chow + BAIBA vs HFD *p* = 0.009, HFD vs HFD + BAIBA *p* = 0.04; Type IIa chow vs HFD *p* = 0.003, chow + BAIBA vs HFD + BAIBA *p* = 0.02, HFD vs HFD + BAIBA *p* = 0.000001; Two-way ANOVA). **f** Specific soleus contractile force (N/cm^2^) under increasing stimuli (Hz) from chow or HFD-fed mice receiving BAIBA compared to untreated controls (chow *n* = 14, chow + BAIBA *n* = 17, HFD *n* = 17, HFD + BAIBA *n* = 15; 15 Hz chow vs chow + BAIBA *p* = 0.0495; 30 Hz chow vs chow + BAIBA *p* = 0.034, 50 Hz HFD vs HFD + BAIBA *p* = 0.045; 80 Hz chow vs chow + BAIBA *p* = 0.037, chow vs HFD *p* = 0.026, HFD vs HFD + BAIBA *p* = 0.007; 120 Hz chow vs chow + BAIBA *p* = 0.032, chow vs HFD *p* = 0.003, HFD vs HFD + BAIBA *p* = 0.0005; 150 Hz chow vs chow + BAIBA *p* = 0.0009, chow vs HFD *p* = 0.026, HFD vs HFD + BAIBA *p* = 0.0003; Two-way ANOVA).). *n* = individual mouse. **g** Relative soleus specific force over 300 s in chow or HFD-fed mice receiving BAIBA compared to untreated controls (chow *n* = 14, chow + BAIBA *n* = 15, HFD *n* = 11, HFD + BAIBA *n* = 11; 60 s chow vs chow + BAIBA *p* = 0.003, HFD vs HFD + BAIBA *p* = 0.0005; 120 s chow vs chow + BAIBA *p* = 0.022, HFD vs HFD + BAIBA *p* = 0.019; 180 s chow vs chow + BAIBA *p* = 0.04, HFD vs HFD + BAIBA *p* = 0.016; 240 s HFD vs HFD + BAIBA *p* = 0.043; 300 s HFD vs HFD + BAIBA *p* = 0.05; Two-way ANOVA). *n* = individual mouse. **h** Soleus high-resolution respirometry analysis from chow and HFD-fed mice receiving BAIBA compared to untreated controls assessed for fatty acid oxidation (FAO), complex I (I), maximal complex I-supported oxidative phosphorylation (I + ADP), complex I & II-supported oxidative phosphorylation (I + II), maximal uncoupled substrate oxidation (ETC) and complex II (II) (chow = 16, chow + BAIBA = 16, HFD = 15, HFD + BAIBA = 15; FAO chow vs chow + BAIBA *p* = 0.005, chow + BAIBA vs HFD *p* = 0.045; I chow vs chow + BAIBA *p* = 0.043; I + ADP chow + BAIBA vs HFD *p* = 0.05; I + II chow + BAIBA vs HFD *p* = 0.006; ETC chow vs chow + BAIBA *p* = 0.016, chow vs HFD *p* = 0.011, chow + BAIBA vs HFD *p* = 0.0003, HFD vs HFD + BAIBA *p* = 0.012; II chow vs HFD *p* = 0.014, chow + BAIBA vs HFD *p* = 0.004, chow + BAIBA vs HFD + BAIBA *p* = 0.02, HFD vs HFD + BAIBA *p* = 0.05; Two-way ANOVA).). *n* = individual mouse. **i** High-resolution respirometry complex IV activity assay in soleus from chow and HFD-fed mice receiving BAIBA compared to untreated controls (chow = 16, chow + BAIBA = 16, HFD = 15, HFD + BAIBA = 15; chow vs chow + BAIBA *p* = 0.037, chow vs HFD *p* = 0.024, HFD vs HFD + BAIBA *p* = 0.06; One-way ANOVA). *n* = individual mouse. **j** Principal component analysis plot of RNAseq data from the soleus of HFD-fed mice with and without BAIBA treatment (HFD *n* = 10, HFD + BAIBA *n* = 10). **k** Volcano plot of differentially expressed genes from RNAseq data from the soleus of HFD-fed (red) and HFD-fed and BAIBA-treated (blue) mice (*n* = 5; fold-change cut-off = 1.2; adjusted *p* value cut-off = 0.05; Wald test with Benjamini-Hochberg). **l** Gene Set Enrichment Analysis of the RNAseq data from the soleus of HFD-fed mice with and without BAIBA treatment (*n* = 5) (Fisher exact test adjusted for false discovery rate). Data in bar charts are mean ± SEM with data points shown. Box and whisker plots show 25th to 75th percentile (box) min to max (whiskers), mean (+) and median (−). Chow = red fill, Chow + BAIBA = blue fill, HFD = red outline, HFD + BAIBA = blue outline. * *p* ≤ 0.05, ** *p* < 0.01, *** *p* < 0.001, **** *p* < 0.0001. Source data are provided as a Source Data file.

Muscle dysfunction associated with T2D is characterized by decreased mitochondrial quality, function and content^35^ and pathological remodelling of muscle structure^36–38^. Together these pathological phenotypes contribute to exercise intolerance, and reduced muscle mass and strength^39–41^. We investigated whether BAIBA counters obesity and T2D-induced skeletal muscle dysfunction and exercise intolerance. C57BL6/J mice were either fed a chow or 60% high fat diet (HFD) for 8 weeks. Mice were then subcategorized with half the chow and HFD-fed mice given 100 mg/kg/day BAIBA for a further 14 weeks. The remaining mice continued on either the chow or HFD regimen alone. This study design modelled a therapeutic intervention (Supplementary Fig. 5c). BAIBA treatment resulted in increased plasma BAIBA concentration within the physiological range (Supplementary Fig. 5d). BAIBA treatment significantly reduced weight gain (Supplementary Fig. 5e), visceral adiposity (visceral white adipose tissue fat fraction by Magnetic Resonance Imaging) (Supplementary Fig. 5f and 5g), and improved glucose tolerance (Supplementary Fig. 5h and 5i). To investigate the effect of BAIBA on voluntary exercise performance, we examined the wheel running response of these mice following a 3-day acclimation period, using CLAMS. In the chow fed condition the prolonged BAIBA-treated mice exhibited enhanced wheel running capacity (Fig. 5a). As expected the HFD-induced model of obesity demonstrated an impaired exercise response with reduced 24 hr, dark and light phase total wheel running rotations (Fig. 5a). BAIBA treatment of the HFD-fed mice showed therapeutic potential by improving the full 24 hr running capacity of the mice and completely rescued the impaired exercise response in the Light phase (Fig. 5a). BAIBA improves voluntary exercise performance and is therapeutic for obesity-induced exercise intolerance in our mouse model.

Next, we characterised the effect of BAIBA treatment on muscle morphology in the HFD-induced obese mouse model. BAIBA treatment increased the soleus, tibialis anterior and extensor digitorum longus (Fig. 5b) muscle mass of normal chow and HFD-fed mice. We used immunofluorescence to determine the effect of BAIBA on HFD-mediated changes in soleus muscle fiber-type composition (Fig. 5c). HFD feeding reduced total soleus myofiber number, which was inhibited by BAIBA-treatment (Fig. 5d). HFD-feeding remodelled soleus myofiber distribution, reducing the number of type I and type IIa myofibers (Fig. 5e). BAIBA-treatment prevented HFD-induced pathological remodelling of the soleus (Fig. 5e).

For functional assessment, the soleus muscle was removed from BAIBA-treated HFD-fed mice and relevant controls and underwent in vitro force-frequency and fatigue protocols^27^. BAIBA treatment rescues HFD-induced muscle contractile dysfunction (Fig. 5f). BAIBA treatment also increased the resistance to muscle fatigue in the HFD-induced model of T2D (Fig. 5g). Therefore, BAIBA protects muscle from HFD-induced muscle weakness.

We used high-resolution respirometry to assess mitochondrial function in permeabilised soleus muscle fibers (6-8 fiber bundle, in duplicate) from chow and HFD-fed BAIBA-treated and control mice^42,43^. BAIBA treatment enhanced soleus mitochondrial respiration in chow fed mice (Fig. 5h). HFD-induced obesity led to impaired soleus mitochondrial respiration, an effect that was rescued with concomitant BAIBA treatment (Fig. 5h). Using respirometry to assess mitochondrial complex IV activity as a proxy for mitochondrial number determined that BAIBA rescued depleted mitochondrial number in soleus of HFD-induced obese mice (Fig. 5i). These data suggest that BAIBA protects muscle from HFD-induced mitochondrial dysfunction.

To better characterise the molecular mechanisms underlying BAIBA-induced improvements in muscle contractile and mitochondrial function in obesity we performed RNAseq analysis of soleus from BAIBA-treated HFD-fed mice compared with HFD-fed controls (https://www.ebi.ac.uk/arrayexpress/experiments/E-MTAB-14747). Between HFD-fed controls and HFD-fed BAIBA-treated mice, there were 251 DEGs (adjusted *p*-value < 0.05), 135 DEGs upregulated and 116 DEGs downregulated (Fig. 5j, k). As in the mice treated for 6 weeks with BAIBA, gene enrichment analysis identified that BAIBA increases the expression of genes for muscle cell differentiation (*Smo*/*Ramp2*/*Lama2*/*Hes1*/*Uchl1*/*Ednrb*/*Fgf10*/*Myof*/*Zbed6*/*Neu2*/*Ankrd2*/*Hacd1*, *p* < 0.02). BAIBA treatment also had a significant effect on the expression of genes for mitochondrial oxidative phosphorylation (*Cycs*/*Uqcrb*/*Ndufa9*/*Ndufv2*/*Dnajc15*/*Chchd10*/*Ndufc2*/*Atp5pb*/*Sdhb*/*Ndufb3*/*Ndufs3*, *p* < 0.0001), oxygen transport (haptoglobin-hemoglobin complex; *Hba-a1*/*Hba-a2*/*Hbb-bt*, *p* < 0.01), response to muscle stretch (*Fos*/*Ankrd2*/*Pik3ca*, *p* < 0.02), and regulation of protein catabolism (*Map1a*/*Abca2*/*Atp13a2*/*Egln2*/*Asb11*/*Psme1*/*Timp2*/*Nkd2*/*Sdcbp*/*Sh3d19*/*Mapk8*, *p* < 0.014). Again, we performed GSEA^28^ of the significant DEGs searching against the MSigDB Molecular Signatures Database^29^. This analysis identified that BAIBA induces transcriptional processes in the HFD-fed mice consistent with the earlier observations made in our 6 week treatment model. BAIBA induced transcriptional remodelling indicative of skeletal muscle structural and metabolic remodelling including oxidative phosphorylation (adjusted *p* value 3.65e^–20^), mesenchymal remodelling (adjusted *p* value 4.76e^–15^), and myogenesis (adjusted *p* value 1.19e^−8^), (Fig. 5l). Together, these data demonstrate BAIBA rescues impaired muscle mitochondrial function to improve contractile fatigue resistance in a model of obesity and T2D-induced muscle dysfunction.

### Peroxisome proliferator-activated receptor δ mediates BAIBA-induced skeletal muscle adaptation

**Fig. 6:**
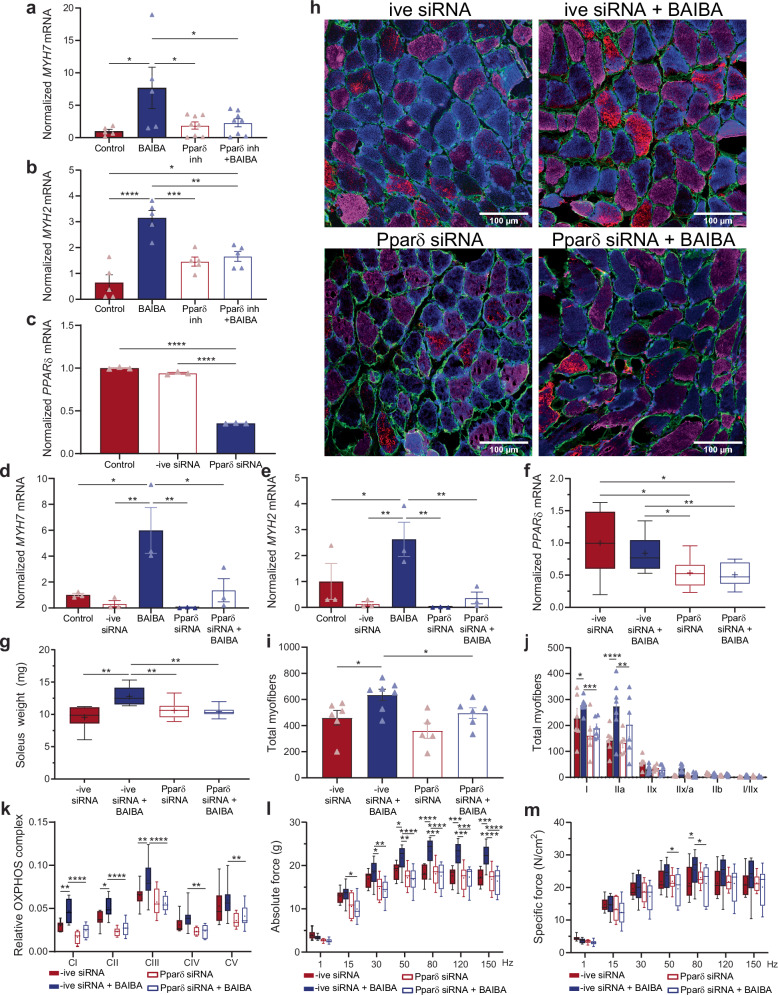
BAIBA mediates skeletal muscle remodelling through Peroxisome Proliferator-Activated Receptor δ. **a** *MYH7* expression in Human Skeletal Muscle Cells (HSkMCs) differentiated to myotubes treated with vehicle control, BAIBA (10 μM), PPARδ inhibitor (PPARδ inh) GSK 3787 (1 μM) or both BAIBA (10 μM) and the PPARδ inh (1 μM) (Control, BAIBA *n* = 5; PPARδ inh, PPARδ inh + BAIBA *n* = 8; control vs BAIBA *p* = 0.02; BAIBA vs PPARδ inh *p* = 0.027; BAIBA vs PPARδ inh + BAIBA *p* = 0.04; One-way ANOVA). *n* = number of biological replicates as independent culture plates. **b** *MYH2* expression in HSkMCs differentiated to myotubes treated with vehicle control, BAIBA (10 μM), PPARδ inh GSK 3787 (1 μM) or both BAIBA (10 μM) and the PPARδ inh (1 μM). (*n* = 5; control vs BAIBA *p* = 0.00001; BAIBA vs PPARδ inh *p* = 0.0008; BAIBA vs PPARδ inh + BAIBA *p* = 0.0026; One-way ANOVA). *n* = number of biological replicates as independent culture plates. **c** *PPARδ* expression in myotubes treated with negative control scrambled siRNA (-ive siRNA) and siRNA against PPARδ (*n* = 3; control vs PPARδ siRNA *p* = 0.00000000001, -ive siRNA vs PPARδ siRNA *p* = 0.0000000001; One-way ANOVA). *n* = number of biological replicates as independent culture plates. **d** *MYH7* expression in myotubes treated with -ive siRNA, BAIBA (10 μM) and PPARδ siRNA and BAIBA and PPARδ siRNA (*n* = 3; control vs BAIBA *p* = 0.019, -ive siRNA vs BAIBA *p* = 0.008, BAIBA vs PPARδ siRNA *p* = 0.0058, BAIBA vs PPARδ siRNA + BAIBA *p* = 0.029; One-way ANOVA). *n* = number of biological replicates as independent culture plates. **e** *MYH2* expression in myotubes treated with -ive siRNA, BAIBA (10 μM) and PPARδ siRNA and BAIBA and PPARδ siRNA (*n* = 3; control vs BAIBA *p* = 0.027, -ive siRNA vs BAIBA *p* = 0.0026, BAIBA vs PPARδ siRNA *p* = 0.0019, BAIBA vs PPARδ siRNA + BAIBA *p* = 0.005; One-way ANOVA). *n* = number of biological replicates as independent culture plates. **f** Soleus *Pparδ* expression in mice with or without BAIBA treatment and receiving either -ive siRNA or *Pparδ* siRNA into their gastrocnemius (-ive siRNA, -ive siRNA + BAIBA *n* = 8; *Pparδ* siRNA, *Pparδ* siRNA + BAIBA *n* = 10; -ive siRNA vs *Ppadδ* siRNA *p* = 0.037, -ive siRNA vs *Pparδ* siRNA + BAIBA *p* = 0.026, -ive siRNA + BAIBA vs *Pparδ* siRNA *p* = 0.02, -ive siRNA + BAIBA vs *Pparδ* siRNA + BAIBA *p* = 0.005; One-way ANOVA). **g** Soleus mass of mice with or without BAIBA treatment and receiving -ive siRNA or *Pparδ* siRNA into their gastrocnemius (-ive siRNA, -ive siRNA + BAIBA *n* = 8; *Pparδ* siRNA, *Pparδ* siRNA + BAIBA *n* = 10; -ive siRNA vs -ive siRNA + BAIBA *p* = 0.0014, -ive siRNA + BAIBA vs *Pparδ* siRNA *p* = 0.0053, -ive siRNA + BAIBA vs *Pparδ* siRNA + BAIBA *p* = 0.002; One-way ANOVA). *n* = individual mouse. **h** Soleus cross sectional confocal images from mice with or without BAIBA treatment and receiving either -ive siRNA or *Pparδ* siRNA into their gastrocnemius. Myosin heavy chain I (MyHCI; purple), MyHCIIa (blue), MyHCIIb (red), MyHCIIx (black/unstained) and the basal lamina (laminin; green). Scale bar = 100 μm. **i** Total soleus myofibers from mice with or without BAIBA treatment and receiving either -ive siRNA or *Pparδ* siRNA into their gastrocnemius (-ive siRNA *n* = 6, *Pparδ* siRNA *n* = 5, -ive siRNA + BAIBA *n* = 7, *Pparδ* siRNA + BAIBA *n* = 6; -ive siRNA vs –ive siRNA + BAIBA *p* = 0.016; –ive siRNA + BAIBA vs *Pparδ* siRNA + BAIBA *p* = 0.049; One-way ANOVA). *n* = individual mouse. **j** Total soleus myofibers by fibertype (I, IIa, IIx, IIb and hybrid IIx/a and I/IIb) from mice with or without BAIBA treatment and receiving either -ive siRNA or *Pparδ* siRNA into their gastrocnemius (-ive siRNA *n* = 6, *Pparδ* siRNA *n* = 5, -ive siRNA + BAIBA *n* = 7, *Pparδ* siRNA + BAIBA *n* = 6; Type I -ive siRNA vs –ive siRNA + BAIBA *p* = 0.046, –ive siRNA + BAIBA vs *Pparδ* siRNA + BAIBA *p* = 0.0006; type IIa -ive siRNA vs –ive siRNA + BAIBA *p* = 0.0000007, –ive siRNA + BAIBA vs *Pparδ* siRNA + BAIBA *p* = 0.006; Two-way ANOVA). **k** Soleus respiratory complex I-V protein expression from mice with or without BAIBA treatment and receiving either -ive siRNA or *Pparδ* siRNA directly into their hind limbs (-ive siRNA *n* = 7, *Pparδ* siRNA *n* = 10, -ive siRNA + BAIBA *n* = 8, *Pparδ* siRNA + BAIBA *n* = 10; Complex I -ive siRNA vs –ive siRNA + BAIBA *p* = 0.008, –ive siRNA + BAIBA vs *Pparδ* siRNA + BAIBA *p* < 0.0001; Complex II -ive siRNA vs –ive siRNA + BAIBA *p* = 0.025, –ive siRNA + BAIBA vs *Pparδ* siRNA + BAIBA *p* = 0.00007; Complex III -ive siRNA vs –ive siRNA + BAIBA *p* = 0.005, –ive siRNA + BAIBA vs *Pparδ* siRNA + BAIBA *p* = 0.000004; Complex IV –ive siRNA + BAIBA vs *Pparδ* siRNA + BAIBA *p* = 0.008; Complex V –ive siRNA + BAIBA vs *Pparδ* siRNA + BAIBA *p* = 0.001; Two-way ANOVA). **l** Absolute soleus ex vivo contractile force from mice with or without BAIBA treatment and receiving either -ive siRNA or *Pparδ* siRNA into their gastrocnemius (-ive siRNA *n* = 11, -ive siRNA + BAIBA *n* = 10, *Pparδ* siRNA *n* = 7, *Pparδ* siRNA + BAIBA *n* = 9; 15 Hz -ive siRNA + BAIBA vs *Pparδ* siRNA + BAIBA *p* = 0.014; 30 Hz -ive siRNA + BAIBA vs *Pparδ* siRNA + BAIBA *p* = 0.0012, -ive siRNA + BAIBA vs *Pparδ* siRNA *p* = 0.015; 50 Hz -ive siRNA vs -ive siRNA + BAIBA *p* = 0.035, -ive siRNA + BAIBA vs *Pparδ* siRNA *p* = 0.0012, ive siRNA + BAIBA vs *Pparδ* siRNA + BAIBA *p* = 0.00009; 80 Hz -ive siRNA vs -ive siRNA + BAIBA *p* = 0.00002, -ive siRNA + BAIBA vs *Pparδ* siRNA *p* = 0.0002, ive siRNA + BAIBA vs *Pparδ* siRNA + BAIBA *p* = 0.000005; 120 Hz ive siRNA vs -ive siRNA + BAIBA *p* = 0.0004, -ive siRNA + BAIBA vs *Pparδ* siRNA *p* = 0.0003, ive siRNA + BAIBA vs *Pparδ* siRNA + BAIBA *p* = 0.0003; 150 Hz -ive siRNA vs -ive siRNA + BAIBA *p* = 0.0001, -ive siRNA + BAIBA vs *Pparδ* siRNA *p* = 0.00008, ive siRNA + BAIBA vs *Pparδ* siRNA + BAIBA *p* = 0.000008; Two-way ANOVA). (**m**) Soleus specific ex vivo contractile force in mice with or without BAIBA treatment and receiving either -ive siRNA or *Pparδ* siRNA directly into their gastrocnemius (-ive siRNA *n* = 11, -ive siRNA + BAIBA *n* = 10, *Pparδ* siRNA *n* = 7, *Pparδ* siRNA + BAIBA *n* = 9; 50 Hz -ive siRNA + BAIBA vs *Pparδ* siRNA + BAIBA *p* = 0.02; 80 Hz -ive siRNA vs -ive siRNA + BAIBA *p* = 0.017, -ive siRNA + BAIBA vs *Pparδ* siRNA + BAIBA *p* = 0.022; Two-way ANOVA). *n* = individual mouse. Data in bar charts are mean ± SEM with data points shown. Box and whisker plots show 25th to 75th percentile (box) min to max (whiskers), mean (+) and median (−). -ive siRNA = red fill, ive siRNA + BAIBA = blue fill, *Pparδ* siRNA = red outline, *Pparδ* siRNA + BAIBA = blue outline. * *p* ≤ 0.05, ** *p* < 0.01, *** *p* < 0.001, **** *p* < 0.0001. Source data are provided as a Source Data file.

We explored the mechanism through which BAIBA mediates exercise-like adaptation in skeletal muscle. We previously discovered that BAIBA induces adipose tissue browning through peroxisome proliferator-activated receptor α (PPARα) signaling^19^. Another member of this receptor family, PPARδ, regulates skeletal muscle development, oxidative capacity, fiber type and running endurance^44,45^. Our RNAseq analysis in mice and HSkMCs treated with BAIBA indicated activation of PPAR signaling. We hypothesized that PPARδ may mediate the effects of BAIBA on skeletal muscle. Therefore, we co-treated HSkMCs with BAIBA (10 μM) and the pharmacological PPARδ antagonist GW3787 (1 μM). GW3787 is an irreversible highly selective antagonist of PPARδ^46^. Inhibition of PPARδ abrogated BAIBA-induced expression of type I and type II oxidative myofiber markers *MYH7* and *MYH2* in HSkMCs (Fig. 6a, b). We then used siRNA to knockdown PPARδ expression in HSkMCs (Fig. 6c). Co-treatment of HSkMCs with BAIBA (10 μM) and siRNA against PPARδ indicated that knockdown of PPARδ expression inhibited the BAIBA-induced expression of *MYH7* and *MYH2* (Fig. 6d, e).

Next, we determined whether *Pparδ* was required for BAIBA-mediated morphological and functional adaptation of skeletal muscle in vivo. C57BL6/J mice received intramuscular injections (2 per week) to the gastrocnemius of both hind limbs of either siRNA against *Pparδ* or negative control (scrambled) siRNA and either received 100 mg/kg/day BAIBA in their drinking water or remained untreated over a period of 6 weeks. Plasma BAIBA was increased in the BAIBA-treated groups (Supplementary Fig. 6a). siRNA against *Pparδ* significantly reduced soleus *Pparδ* expression (Fig. 6f). Knockdown of *Pparδ* blunted BAIBA-mediated increases in soleus muscle mass (Fig. 6g). We used immunofluorescence to determine the effect of *Pparδ* knockdown on BAIBA-mediated changes in soleus muscle fiber-type composition (Fig. 6h). *Pparδ* knockdown abrogated BAIBA-induced increases in soleus total myofiber number (Fig. 6i) and BAIBA-mediated increases in fatigue resistant type I and IIa fibers (Fig. 6j). We then determined whether BAIBA requires *Pparδ* to enhance mitochondrial protein content in soleus muscle. Immunoblotting for the mitochondrial electron transport chain respiratory complexes (I-V) determined that knockdown of *Pparδ* impaired BAIBA-induced increases in soleus mitochondrial respiratory complexes (Fig. 6k, Supplementary Fig. 6b). Assessment of isolated soleus contractile function in vitro using force-transducer force-frequency protocols identified that knockdown of *Pparδ* inhibited BAIBA-stimulated increases in soleus muscle absolute (Fig. 6L) and specific force (Fig. 6m). Therefore, PPARδ is required to transduce the adaptive effects of BAIBA on skeletal muscle morphology and function.

### L-BAIBA not D-BAIBA enhances voluntary exercise performance and skeletal muscle function in vivo

**Fig. 7:**
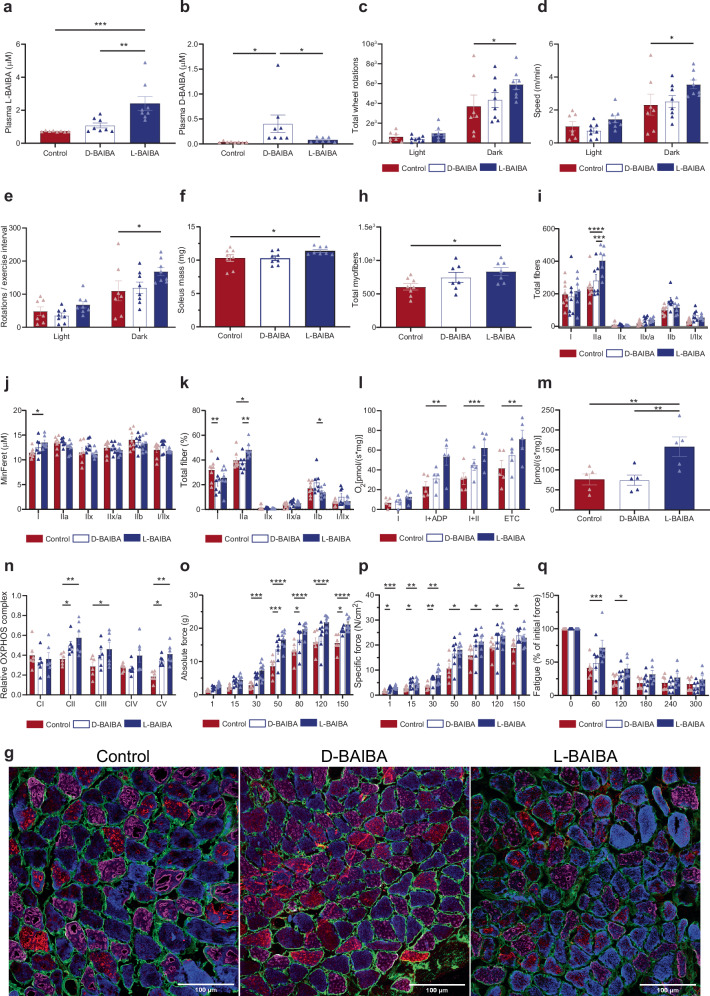
L-BAIBA enhances voluntary exercise performance and skeletal muscle function in vivo. **a** L-BAIBA plasma concentration in mice given either D-BAIBA or L-BAIBA for 6 weeks compared to control mice (*n* = 8; Control vs L-BAIBA *p* = 0.0001, D-BAIBA vs L-BAIBA *p* = 0.0014; One-way ANOVA). **b** D-BAIBA plasma concentration in mice given either D-BAIBA or L-BAIBA for 6 weeks compared to control mice (*n* = 8; Control vs D-BAIBA *p* = 0.019, D-BAIBA vs L-BAIBA *p* = 0.036; One-way ANOVA). (**c**) Total running wheel rotations in the (24 hr light and dark diurnal phase) in mice which have received either D-BAIBA or L-BAIBA for 6 weeks compared to untreated controls (Control *n* = 7, D-BAIBA *n* = 8, L-BAIBA *n* = 8; Dark phase Control vs L-BAIBA, *p* = 0.012; two-way ANOVA). **d** Average running speed (m/min) (24 hr light and dark diurnal phase) of mice which have received either D-BAIBA or L-BAIBA for 6 weeks compared to untreated controls (Control *n* = 7, D-BAIBA *n* = 8, L-BAIBA *n* = 8; Dark phase Control vs L-BAIBA, *p* = 0.016; two-way ANOVA). **e** Running wheel rotation per exercise interval (24 hr light and dark diurnal phase) of mice which have received either D-BAIBA or L-BAIBA for 6 weeks compared to untreated controls (Control *n* = 7, D-BAIBA *n* = 8, L-BAIBA *n* = 8; Dark phase Control vs L-BAIBA, *p* = 0.016; two-way ANOVA). **f** Soleus mass of mice which have received either D-BAIBA or L-BAIBA for 6 weeks compared to untreated controls (Control *n* = 8, D-BAIBA *n* = 8, L-BAIBA *n* = 8; Control vs L-BAIBA, *p* = 0.05; One-way ANOVA). **g** Soleus composite cross sectional confocal images from mice receiving either D-BAIBA or L-BAIBA for 6 weeks compared to untreated controls. Myosin heavy chain I (MyHCI; purple), MyHCIIa (blue), MyHCIIb (red), MyHCIIx (black/unstained), basal lamina (laminin; green). Scale bar = 100 μm. **h** Total soleus myofibers from mice which have received either D-BAIBA or L-BAIBA for 6 weeks compared to untreated controls (Control *n* = 8, D-BAIBA *n* = 7, L-BAIBA *n* = 7; Control vs L-BAIBA, *p* = 0.035; One-way ANOVA). **i** Soleus myofibers by fibertype from mice which have received either D-BAIBA or L-BAIBA for 6 weeks compared to untreated controls (Control *n* = 8, D-BAIBA *n* = 7, L-BAIBA *n* = 7; type IIa fiber Control vs L-BAIBA, *p* = 0.0000009, D-BAIBA vs L-BAIBA *p* = 0.0006, Two-way ANOVA). **j** Soleus myofiber diameter (MinFeret, µm) by fibertype from mice which have received either D-BAIBA or L-BAIBA for 6 weeks compared to untreated controls (Control *n* = 8, D-BAIBA *n* = 7, L-BAIBA *n* = 7; type I fiber Control vs L-BAIBA, *p* = 0.013; Two-way ANOVA). **k** Proportion of soleus myofibers by fibertype (%) (I, IIa, IIx, IIb and hybrid IIx/a and I/IIb) from which have received either D-BAIBA or L-BAIBA for 6 weeks compared to untreated controls (Control *n* = 8, D-BAIBA *n* = 7, L-BAIBA *n* = 7; type I fibers, Control vs D-BAIBA, *p* = 0.0067; type IIa fibers, Control vs L-BAIBA *p* = 0.017, D-BAIBA vs L-BAIBA, *p* = 0.002; Two-way ANOVA). *n* = individual mouse. **l** Soleus high-resolution respirometry analysis from mice which have received either D-BAIBA or L-BAIBA for 6 weeks compared to untreated controls assessed for complex I (I), complex I-supported oxidative phosphorylation (I + ADP), complex I & II-supported oxidative phosphorylation (I + II) and maximal uncoupled substrate oxidation (ETC), corrected for tissue mass (*n* = 5; I + ADP Control vs L-BAIBA, *p* = 0.0011; I + II Control vs L-BAIBA, *p* = 0.0007; max ETC Control vs L-BAIBA, *p* = 0.0013 Two-way ANOVA). **m** Soleus high-resolution respirometry assay for complex IV activity from mice which have received either D-BAIBA or L-BAIBA for 6 weeks compared to untreated controls (*n* = 5; Control vs L-BAIBA *p* = 0.007; D-BAIBA vs L-BAIBA *p* = 0.0062; One-way ANOVA). **n** Soleus protein respiratory complex I-V expression from mice which have received either D-BAIBA or L-BAIBA for 6 weeks compared to untreated controls (*n* = 6; CII, Control vs D-BAIBA *p* = 0.046, Control vs L-BAIBA *p* = 0.002; CIII, Control vs L-BAIBA *p* = 0.01; CV, Control vs D-BAIBA *p* = 0.02, Control vs L-BAIBA *p* = 0.0012; Two-way ANOVA). **o** Soleus absolute ex vivo contractile force from mice which have received either D-BAIBA or L-BAIBA for 6 weeks compared to untreated controls (Control = 6, D-BAIBA = 7, L-BAIBA = 8; 30 Hz Control vs L-BAIBA *p* = 0.0008; 50 Hz Control vs D-BAIBA *p* = 0.0003, Control vs L-BAIBA *p* = 0.000000007; 80 Hz Control vs D-BAIBA *p* = 0.01, Control vs L-BAIBA *p* = 0.000001; 120 Hz Control vs L-BAIBA *p* = 0.000009; 150 Hz Control vs D-BAIBA *p* = 0.026, Control vs L-BAIBA *p* = 0.00004; Two-Way ANOVA). **p** Soleus specific ex vivo contractile force from mice which have received either D-BAIBA or L-BAIBA for 6 weeks compared to untreated controls (Control = 6, D-BAIBA = 7, L-BAIBA = 8; 1 Hz Control vs D-BAIBA *p* = 0.03, Control vs L-BAIBA *p* = 0.0001; 15 Hz Control vs D-BAIBA *p* = 0.03, Control vs L-BAIBA *p* = 0.008; 30 Hz Control vs D-BAIBA *p* = 0.0036, Control vs L-BAIBA *p* = 0.0044; 50 Hz Control vs L-BAIBA *p* = 0.011; 80 Hz Control vs L-BAIBA *p* = 0.02; 120 Hz Control vs L-BAIBA *p* = 0.04; 150 Hz Control vs D-BAIBA *p* = 0.02, Control vs L-BAIBA *p* = 0.049; Two-way ANOVA). **q** Soleus relative specific force over 300 s from mice which have received either D-BAIBA or L-BAIBA for 6 weeks compared to untreated controls (Control = 6, D-BAIBA = 7, L-BAIBA = 6; 60 s Control vs L-BAIBA *p* = 0.0002; 120 s Control vs L-BAIBA *p* = 0.03; Two-way ANOVA). Data in bar charts are mean ± SEM with data points shown. Box and whisker plots show 25th to 75th percentile (box) min to max (whiskers), mean (+) and median (−). Control = red fill, D-BAIBA = blue outline, L-BAIBA = blue fill. * *p* ≤ 0.05, ** *p* < 0.01, *** *p* < 0.001, **** *p* < 0.0001. Source data are provided as a Source Data file.

Physiologically BAIBA is present as two distinct D- and L- enantiomers with L-BAIBA previously identified as secreted from contracting skeletal muscle^20^. Therefore, we examined the effects of D-BAIBA and L-BAIBA enantiomers independently. C57BL6/J mice were either treated with 100 mg/kg/day L-BAIBA or D-BAIBA in drinking water for 6 weeks. Plasma L-BAIBA (Fig. 7a) and D-BAIBA (Fig. 7b) concentrations were increased in their respective treatment groups. At 6 weeks of treatment, mice were placed in a Combined Laboratory Animal Monitoring System to determine physiological phenotypes. L-BAIBA increased oxygen consumption and energy expenditure compared with D-BAIBA and control mice (Supplementary Fig. 7a and 7b). Food intake and basal activity were not affected by D-BAIBA or L-BAIBA treatment (Supplementary Fig. 7c and 7d). As previously, we used free wheel running analysis, preceded by a 3-day habituation period, to examine voluntary exercise performance in these mice. L-BAIBA treated mice had greater total wheel rotations (Fig. 7c), running speed (Fig. 7d) and number of rotations per exercise interval (Fig. 7e). However, L-BAIBA and D-BAIBA treatment does not affect the total number of exercise bouts (Supplementary Fig. 7e) or the total duration of all exercise bouts (Supplementary Fig. 7f). L-BAIBA and not D-BAIBA enhances voluntary exercise performance in mice.

We examined the effects of the distinct BAIBA enantiomers on skeletal muscle phenotype. L-BAIBA, but not D-BAIBA enhanced soleus muscle mass (Fig. 7f). Using immunofluorescence we determined the effect of L-BAIBA and D-BAIBA on soleus muscle fiber-type composition (Fig. 7g–k). L-BAIBA but not D-BAIBA significantly increased the total number of fibers in soleus (Fig. 7h). This effect was primarily driven by a significant increase in type IIa fibers in the soleus of L-BAIBA treated mice (Fig. 7i). L-BAIBA specifically increased the diameter of type I fibers (Fig. 7j). Proportionally, D-BAIBA reduced type I fiber content in the soleus with L-BAIBA inducing fiber remodelling towards a higher percentage of type IIa fibers (Fig. 7k).

Next, we investigated the effect of the D-BAIBA and L-BAIBA enantiomers on skeletal muscle mitochondrial phenotype. Using high-resolution respirometry, we determined that L-BAIBA, and not D-BAIBA, increased soleus mitochondrial electron transport chain (ETC) coupled complex I-mediated oxidative phosphorylation (Fig. 7l). Soleus complex I and II succinate-stimulated respiration was higher in L-BAIBA-treated, but not significantly different in D-BAIBA-treated, mice (Fig. 7l). Maximal uncoupled ETC respiration did not differ from control in D-BAIBA treated mice, but was increased in the soleus of L-BAIBA-treated mice (Fig. 7l). We then used complex IV respiratory activity^26^ to suggest that only L-BAIBA increased soleus mitochondrial content (Fig. 7m). We employed immunoblotting to identify that L-BAIBA treatment increased the protein levels of mitochondrial electron transport respiratory complexes (complex II, III, V) in soleus (Fig. 7n; and Supplementary Fig. 7g) as well as EDL (complexes II, IV and V) (Supplementary Fig. 7h and 7i). Interestingly, D-BAIBA also exhibited the capacity to increase expression of complexes II and V in soleus and II, III and V in EDL. L-BAIBA increases mitochondrial content and respiratory capacity in skeletal muscle.

We then examined whether D- and L-BAIBA-treated mice exhibited improved soleus muscle contractile function using our force-frequency and fatigue protocols^27^. L-BAIBA induced greater soleus force-frequency in mice for both absolute and normalized contractile force (Fig. 7o, p). Although to a lesser extent than L-BAIBA, D-BAIBA also increased absolute and normalized contractile force (Fig. 7o, p). However, only L-BAIBA-treatment, and not D-BAIBA, increased soleus fatigue resistance, consistent with the effects of L-BAIBA on mitochondrial function and number (Fig. 7q).

### L-BAIBA enhances human primary skeletal myocyte differentiation

We observed that the L-BAIBA, rather than the D-BAIBA, enantiomer was primarily responsible for improved muscle phenotype and function in our mouse models. Therefore, we examined the effect of L-BAIBA on markers of HSkMC differentiation in vitro. HSkMCs were seeded and immediately treated with 10μM L-BAIBA. L-BAIBA increased expression of key regulatory genes for skeletal muscle differentiation 72 h post-seeding including paired box 7 (*PAX7*), *MYF5* and *MYF6* (Supplementary Fig. 8a). Then, we treated myoblasts with 10μM L-BAIBA during a six day in vitro differentiation period. For a direct assessment of differentiation and myoblast fusion we used immunohistochemistry to stain the L-BAIBA-treated and differentiated myotubes for muscle myosin (MF20), nuclei (Hoechst) and the marker of differentiation, myogenin (Supplementary Fig. 8b). L-BAIBA increased the differentiation index (Supplementary Fig. 8c), and fusion index (Supplementary Fig. 8d) of the HSkMCs. We investigated the molecular phenotype induced by L-BAIBA in HSkMCs with RNAseq in 10μM L-BAIBA-treated HSKMCs compared with controls (https://www.ebi.ac.uk/arrayexpress/experiments/E-MTAB-14746). Our analysis identified 460 unique and significantly DEGs between L-BAIBA-treated and control myocytes, with 247 DEGs up- and 213 DEGs down-regulated (Supplementary Fig. 8e). Using GSEA^28^ of the significant DEGs searching against the MSigDB Molecular Signatures Database^29^ (Supplementary Fig. 8f), we found that the top 2 most enriched terms with L-BAIBA treatment, mesenchymal remodelling (*p* = 1.68e^-54^) and myogenesis (*p* = 4.89e^-28^) were consistent with our in vivo results for BAIBA induced transcriptional processes in muscle of both our HFD-fed mice and our 6 week BAIBA treatment model and our previous study of human primary myotubes.

### Circulating L-BAIBA concentrations correlate to aerobic fitness and increase in response to acute aerobic exercise in humans

**Fig. 8:**
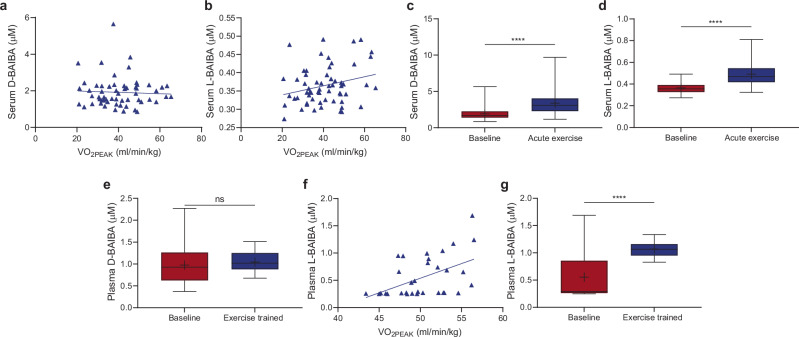
Circulating L-BAIBA concentrations correlate to aerobic fitness and increase in response to acute aerobic exercise and exercise training in humans. **a** Serum D-BAIBA concentration does not correlate with VO_2PEAK_ in humans (*n* = 60; two-tailed Pearson correlation *r* = 0.05, *p* = 0.69). **b** Serum L-BAIBA concentration correlates with VO_2PEAK_ in humans (*n* = 60; two-tailed Pearson correlation *r* = 0.25, *p* = 0.046). (**c**) Serum D-BAIBA concentration is increased in humans following an acute exercise treadmill intervention_._ (*n* = 60 individual participants, *p* = 0.000000000000001, paired two-tailed t-test; Baseline = red; Acute exercise = blue). **d** Serum L-BAIBA concentration is increased in humans following an acute exercise treadmill intervention_._ (*n* = 60 individual participants, *p* = 0.000000000000001, paired two-tailed t-test Baseline = red; Acute exercise = blue). **e** Plasma D-BAIBA concentration in participants at baseline and following a 10-week aerobic exercise training program (*n* = 33 individual participants, *p* = 0.37, paired two-tailed t-test; Baseline = red; Exercise trained = blue). **f** Plasma L-BAIBA concentration correlates with VO_2PEAK_ in an independent human cohort (*n* = 33; two-tailed Pearson correlation *r* = 0.49, *p* = 0.004). **g** Plasma L-BAIBA concentration in participants at baseline and following a 10-week aerobic exercise training program (*n* = 33 individual participants, *p* = 0.00000002, paired two-tailed t-test; Baseline = red; Exercise trained = blue). Box and whisker plots show 25th to 75th percentile (box) min to max (whiskers), mean (+) and median (−). ns = not significant, ** *p* < 0.01, **** *p* < 0.0001. Source data are provided as a Source Data file.

We investigated the relationship between circulating levels of L-BAIBA and D-BAIBA and both acute aerobic endurance exercise and fitness in humans. Maximal aerobic capacity was assessed in sixty human volunteers (Supplementary Table 1) using a peak pulmonary oxygen uptake (VO_2PEAK_) cardiopulmonary exercise test on a treadmill. At a secondary study visit resting fasted serum was taken from the volunteers prior to undergoing an acute endurance exercise treadmill intervention for 45 minutes at 60% VO_2PEAK_. Immediately at exercise completion a second serum sample was taken to assess changes in response to acute aerobic exercise. L-BAIBA and D-BAIBA serum concentrations both at baseline and immediately after aerobic exercise were measured by LC-MS analysis in the volunteers. Serum L-BAIBA and D-BAIBA did not significantly correlate with age in our study population (Supplementary Fig. 9a and 9b). Baseline resting D-BAIBA serum concentration did not correlate with VO_2PEAK_ (Fig. 8a). However, baseline resting serum L-BAIBA was significantly positively correlated with VO_2PEAK_ (*r* = 0.26, *p-*value = 0.046) (Fig. 8b). These data suggest circulating serum L-BAIBA concentration associates with aerobic fitness in humans.

Next, we compared the levels of D-BAIBA and L-BAIBA in the serum at baseline and immediately after the acute exercise treadmill intervention for 45 minutes at 60% VO_2PEAK_. Both D-BAIBA and L-BAIBA were increased in the serum in response to acute aerobic exercise (Fig. 8c, d). Exercise-induced increases in serum D- and L-BAIBA occurred in both male and female participants (Supplementary Fig. 9c,f). Acute aerobic exercise induces increases in circulating L-BAIBA and D-BAIBA levels in humans.

### Serum L-BAIBA increases in response to endurance exercise training in humans

We then assessed the relationship between plasma D-BAIBA and L-BAIBA and endurance exercise training in 33 human participants (Supplementary Table 2). We analyzed D-BAIBA and L-BAIBA concentrations in the plasma from volunteers taken pre and post a 10-week aerobic exercise cycle ergometer-based training program^47^. Exercise training increased volunteer VO_2PEAK_^47^. LC-MS analysis demonstrated that circulating levels of D-BAIBA in blood plasma of volunteers did not increase in response to exercise training (Fig. 8e). Again, L-BAIBA correlated to VO_2PEAK_ at baseline in this independent group of volunteers (*r* = 0.49; *p* = 0.004) (Fig. 8f). L-BAIBA plasma concentration was increased by endurance exercise training (Fig. 8g). Endurance exercise training increased circulating levels of L-BAIBA in humans.

### Skeletal muscle 4-aminobutyrate aminotransferase (ABAT)-mediated L-BAIBA synthesis is required for exercise training-induced enhancement of exercise performance

**Fig. 9:**
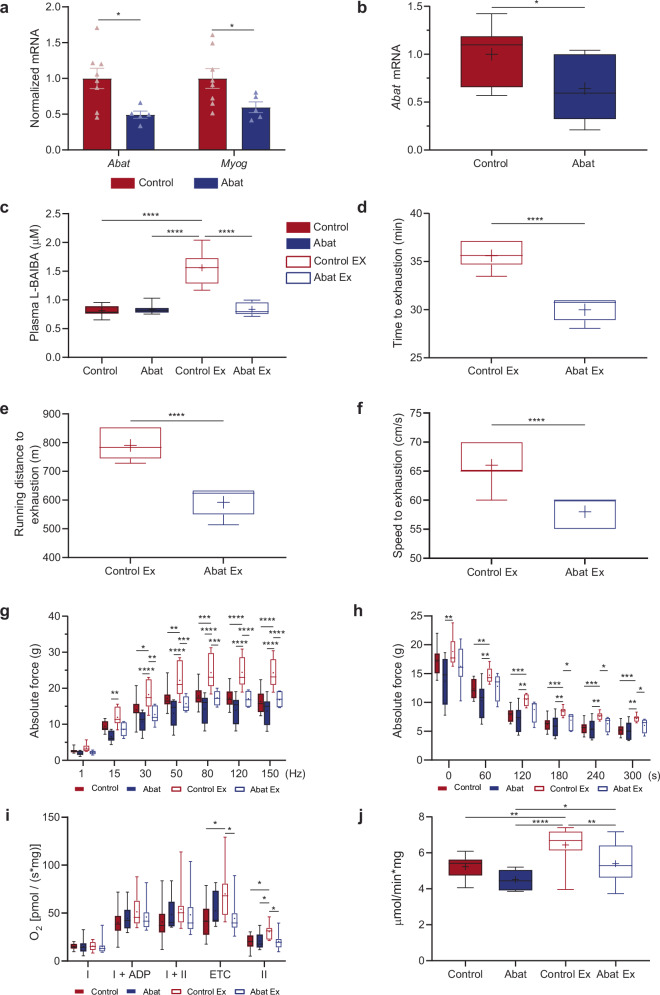
Muscle *Abat*-mediated BAIBA synthesis contributes to exercise training-induced muscle adaptation. **a** Soleus *Abat* and *Myog* expression of mice receiving either scrambled negative control GapmeR (Control) or GapmeR against *Abat* (*Abat*) into the gastrocnemius (Control *n* = 8, *Abat* Knockdown *n* = 5; *Abat* Control vs *Abat* Knockdown *p* = 0.02; *Myog* Control vs *Abat* Knockdown *p* = 0.05; two-tailed t-test). **b** Gastrocnemius *Abat* expression of mice receiving either control GapmeR (Control) or *Abat* GapmeR (*Abat*) into the gastrocnemius (*n* = 10; Control vs *Abat p* = 0.017; two-tailed t-test). **c** Plasma L-BAIBA concentration in mice receiving either control GapmeR (Control) or *Abat* GapmeR (*Abat*) into the gastrocnemius with or without treadmill exercise (Ex) training (*n* = 10; Control GapmeR vs Control GapmeR Ex *p* = 0.000000000001, *Abat* GapmeR vs Control GapmeR Ex *p* = 0.000000000002, Control GapmeR Ex vs Abat GapmeR Ex *p* = 0.000000000003; One-way ANOVA). **d** Maximal running capacity time to exhaustion from mice receiving control GapmeR (Control) or GapmeR against *Abat* (*Abat*) into the gastrocnemius with treadmill exercise (Ex) training (*n* = 10; *p* = 0.00000002; two-tailed t-test). **e** Maximal running capacity running distance to exhaustion of mice receiving either control GapmeR (Control) or *Abat* GapmeR (*Abat*) into the gastrocnemius with treadmill exercise (Ex) training (*n* = 10; *p* = 0.0000001; two-tailed t-test). **f** Maximal running capacity speed to exhaustion of mice receiving either control GapmeR (Control) or *Abat* GapmeR (*Abat*) into the gastrocnemius with treadmill exercise (Ex) training (*n* = 10 individual mice / group; *p* = 0.000008; two-tailed t-test). **g** Soleus absolute ex vivo contractile force from mice receiving either control GapmeR (Control) or *Abat* GapmeR (*Abat*) into the gastrocnemius with or without treadmill exercise (Ex) training (control *n* = 9, Abat *n* = 9, control Ex *n* = 7, Abat Ex *n* = 7; 15 Hz Abat vs control Ex *p* = 0.004; 30 Hz control vs control Ex *p* = 0.03, Abat vs control Ex *p* = 0.00001, control Ex vs Abat Ex *p* = 0.005; 50 Hz control vs Abat *p* = 0.025, control vs control Ex *p* = 0.007, Abat vs control Ex *p* = 0.00000008, control Ex vs Abat Ex *p* = 0.0006; 80 Hz control vs control EX *p* = 0.0002, Abat vs control EX *p* = 0.000000002, control Ex vs Abat Ex *p* = 0.0001; 120 Hz control vs control EX *p* = 0.00004, Abat vs control EX *p* = 0.0000000006, control Ex vs Abat Ex *p* < 0.00006; 150 Hz control vs control EX *p* = 0.000003, Abat vs control EX *p* = 0.0000000003, control Ex vs Abat Ex *p* = 0.00004; Two-way ANOVA). *n* = individual mouse. **h** Soleus absolute contractile force over 300 s of mice receiving either control GapmeR (Control) or *Abat* GapmeR (*Abat*) into the gastrocnemius with or without treadmill exercise (Ex) training (control *n* = 9, Abat *n* = 9, control Ex *n* = 7, Abat Ex *n* = 7; 0 s Abat vs control Ex *p* = 0.008; 60 s control vs control Ex *p* = 0.0068, Abat vs control EX *p* = 0.0017; 120 s control vs control Ex *p* = 0.0003, Abat vs control Ex *p* = 0.0012, control Ex vs Abat Ex *p* = 0.05; 180 s control vs control Ex *p* = 0.0007, Abat vs control Ex *p* = 0.0033, control Ex vs Abat Ex *p* = 0.03; 240 s control vs control Ex *p* = 0.0007, Abat vs control Ex *p* = 0.004, control Ex vs Abat Ex *p* = 0.04; 300 s control vs control Ex *p* = 0.0005, Abat vs control Ex *p* = 0.004, control Ex vs Abat Ex *p* = 0.04; Two-way ANOVA). **i** Soleus h**i**gh**-**resolution respirometry from mice receiving either control GapmeR (Control) or *Abat* GapmeR (*Abat*) into the soleus with or without treadmill exercise (Ex) training (*n* = 10; electron transport chain (ETC) control vs control Ex *p* = 0.013, control Ex vs Abat Ex *p* = 0.02; complex II (II) control vs control Ex *p* = 0.011, Abat vs control Ex *p* = 0.02, control Ex vs Abat Ex *p* = 0.015; One-way ANOVA). **j** Soleus citrate synthase activity (μmol/min/mg) of mice receiving either control GapmeR (Control) or *Abat* GapmeR (*Abat*) into the soleus with or without treadmill exercise (Ex) training (*n* = 10; control vs control Ex *p* = 0.002, Abat vs control Ex *p* < 0.0001, Abat vs Abat Ex *p* = 0.02, control Ex vs Abat Ex *p* = 0.008; One-way ANOVA). Data in bar charts are mean ± SEM with data points shown. Box and whisker plots show 25th to 75th percentile (box) min to max (whiskers), mean (+) and median (−). Control GapmeR (Control; solid red), GapmeR against *Abat* (*Abat*; solid blue), Control GapmeR and Exercise (Control; outline red), GapmeR against *Abat* and Exercise (*Abat*; outline blue). * *p* ≤ 0.05, ** *p* < 0.01, *** *p* < 0.001, **** *p* < 0.0001. Source data are provided as a Source Data file.

Circulating L-BAIBA associated with aerobic fitness in humans. In mice, L-BAIBA enhanced voluntary wheel running capacity and skeletal muscle mitochondrial function, number and contractile fatigue resistance. L-BAIBA is synthesized from the catabolism of valine, with the final step catalysed by the mitochondrial enzyme, 4-aminobutyrate aminotransferase (ABAT)^48,49^. We investigated the importance of skeletal muscle L-BAIBA synthesis for exercise performance in response to training and muscle adaptation to exercise. Age-matched C57BL6/J mice were divided into an unexercised group and a group that underwent a 6 week treadmill exercise training programme 4 days a week at 25 cm/s and 10% grade during a 45-minute session per day. These groups were further divided into groups receiving either scrambled short DNA antisense oligonucleotide (Gapmers) or short DNA antisense oligonucleotide against *Abat*, intramuscularly into the gastrocnemius muscles of both hind limbs twice a week to knockdown *Abat* expression (Supplementary Fig. 10a). Hindlimb skeletal muscle *Abat* expression was significantly reduced in soleus and gastrocnemius (Fig. 9a, b). BAIBA treatment increased myogenin expression in mouse soleus (Fig. 1e) and human primary myotubes (Fig. 4e). Consistently, *myog* expression was decreased in the soleus with *Abat* knockdown (Fig. 9a). *Abat* expression was unaffected in other tissues including the liver (Supplementary Fig. 10b). LC-MS analysis of plasma from the mice indicated *Abat* knockdown impaired the exercise-induced increase in circulating L-BAIBA concentration (Fig. 9c) without affecting D-BAIBA concentration (Supplementary Fig. 10c). *Abat* knockdown and control exercise-trained mice then underwent a maximal treadmill running test. *Abat* knockdown reduced the aerobic exercise performance of the mice compared to the exercise-trained control mice. Exercised *Abat* knockdown mice had reduced time to exhaustion (Fig. 9d), reduced total running distance to exhaustion (Fig. 9e) and reduced speed at exhaustion (Fig. 9f) compared to controls. Together, these data indicate that *Abat*-mediated BAIBA generation in muscle contributes to the adaptive improvement in exercise performance in response to training.

We then focussed on the role of ABAT-mediated L-BAIBA generation in adaptation of the muscle metabolic and functional phenotype in response to exercise training. Contractile function of isolated soleus from the exercised and *Abat* knockdown mice and relevant controls was analysed with our force-frequency protocols^27^. Soleus absolute force frequency was reduced in muscle with reduced *Abat* expression (Fig. 9g). As expected, exercise training enhanced muscle contractile force (Fig. 9g). However, exercise-induced improvements in muscle contractile function were ablated in muscle with decreased *Abat* expression (Fig. 9g). Soleus fatigue resistance was also enhanced by exercise training (Fig. 9h). Exercise training-induced improvements in muscle fatigue resistance were reduced by *Abat* knockdown (Fig. 9h). High-resolution respirometry analysis of soleus mitochondrial function indicated exercise-training enhanced maximal uncoupled respiration (CCCP) and complex II-mediated (ROT) respiration (Fig. 9i). Muscle *Abat* knockdown impaired exercise training-induced increases in maximal uncoupled respiration and complex II-mediated respiration (Fig. 9i). Analysis of mitochondrial content of soleus muscle (citrate synthase activity assay) in the *Abat* knockdown mice with and without exercise training compared to controls indicated exercise training increased the mitochondrial content of the muscle (Fig. 9j). Reduced muscle *Abat* expression impaired exercise training-induced increases in muscle mitochondrial content (Fig. 9j). These data demonstrate that muscle *Abat*-mediated L-BAIBA synthesis contributes to the metabolic and functional adaptation of muscle to exercise.

### L-BAIBA-induced expression of skeletal muscle differentiation and fiber-type markers requires Mas-related G protein-coupled receptor D in human primary myocytes

**Fig. 10:**
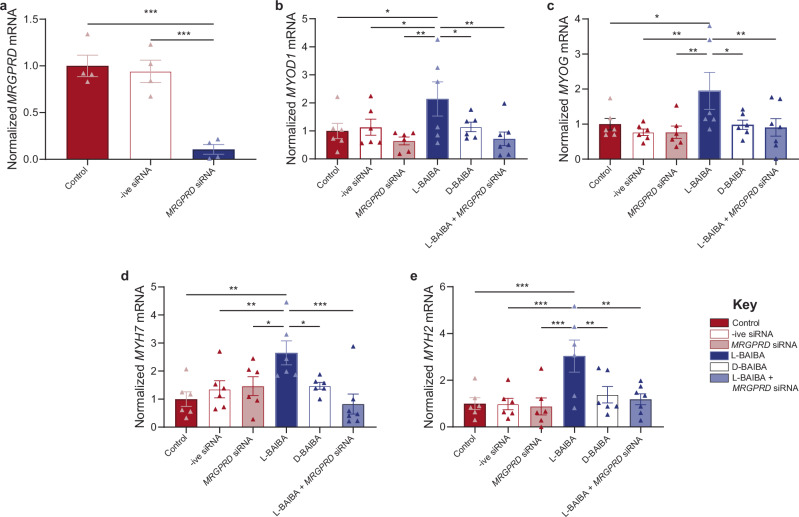
MRGPRD mediates L-BAIBA-induced myocyte differentiation and fiber type gene expression in human primary myocytes. **a** *Mas-related g-protein coupled receptor d* (*MRGPRD*) expression in human myotubes treated with a scrambled control siRNA (-ive siRNA) or an siRNA against MRGPRD (MRGPRD siRNA) (*n*  =  4; Con *P*  =  0.0001, -ive siRNA P  =  0.0002; One-way ANOVA). **b** *Myogenic Differentiation 1* (MYOD1) expression in myotubes treated with -ive siRNA, MRGPRD siRNA, L-BAIBA (10 μM), D-BAIBA (10 μM), and L-BAIBA (10 μM) and MRGPRD siRNA (L-BAIBA + MRGPRD siRNA) (control vs L-BAIBA *p* = 0.019, -ive siRNA vs L-BAIBA *p* = 0.037, L-BAIBA vs MRGPRD siRNA *p* = 0.0028, L-BAIBA vs D-BAIBA *p* = 0.039, L-BAIBA vs L-BAIBA + MRGPRD siRNA *p* = 0.003; One-way ANOVA). **c** *Myogenin* (*MYOG*) expression in myotubes treated with -ive siRNA, MRGPRD siRNA, L-BAIBA, D-BAIBA, and L-BAIBA + MRGPRD siRNA (control vs L-BAIBA *p* = 0.019, -ive siRNA vs L-BAIBA *p* = 0.0043, L-BAIBA vs MRGPRD siRNA *p* = 0.0043, L-BAIBA vs D-BAIBA *p* = 0.017, L-BAIBA vs L-BAIBA + MRGPRD siRNA *p* = 0.0082; One-way ANOVA). **d** *Myosin Heavy Chain 7* (*MYH7*) expression in myotubes treated with -ive siRNA, MRGPRD siRNA, L-BAIBA, D-BAIBA, and L-BAIBA + MRGPRD siRNA (control vs L-BAIBA *p* = 0.001, -ive siRNA vs L-BAIBA *p* = 0.008, L-BAIBA vs MRGPRD siRNA *p* = 0.014, L-BAIBA vs D-BAIBA *p* = 0.015, L-BAIBA vs L-BAIBA + MRGPRD siRNA *p* = 0.0002; One-way ANOVA). **e** *Myosin Heavy Chain 2* (*MYH2*) expression in myotubes treated with -ive siRNA, MRGPRD siRNA, L-BAIBA, D-BAIBA, and L-BAIBA + MRGPRD siRNA (control vs L-BAIBA *p* = 0.0008, -ive siRNA vs L-BAIBA *p* = 0.0007, L-BAIBA vs MRGPRD siRNA *p* = 0.0004, L-BAIBA vs D-BAIBA *p* = 0.005, L-BAIBA vs L-BAIBA + MRGPRD siRNA *p* = 0.0014; One-way ANOVA). **b**–**e** control, -ive siRNA, MRGPRD siRNA, L-BAIBA, D-BAIBA *n* = 6; L-BAIBA + MRGPRD siRNA *n* = 7. Data in bar charts are mean ± SEM with data points shown. Control = solid red; -ive siRNA = red outline; MRGPRD siRNA = light red fill; L-BAIBA = blue fill; D-BAIBA = blue outline; L-BAIBA + MRGPRD siRNA = light blue fill. * *p* ≤ 0.05, ** *p* < 0.01, *** *p* < 0.001. *n* = number of biological replicates as independent culture plates. Source data are provided as a Source Data file.

We further explored the mechanisms for BAIBA-induced regulation of skeletal muscle gene expression. We previously determined that BAIBA increased expression of markers of terminal differentiation towards oxidative muscle fibers, *MYH7* and *MYH2*, and regulatory genes for skeletal muscle differentiation *MYOD1*, and *MYOG* in human primary myocytes. In osteocytes, BAIBA activity requires the cell surface G-protein coupled receptor, Mas-related G protein-coupled receptor D (MRGPRD)^20^. To determine whether MRGPRD is required to transmit BAIBA signalling in human myocytes we used siRNA-mediated silencing to knockdown *MRGPRD* expression in human primary myocytes by 89% (Fig. 10a) with and without L-BAIBA (10 μM) treatment during a six day in vitro differentiation period. Knockdown of MRGPRD impaired L-BAIBA induced expression of *MYOD1* (Fig. 10b), *MYOG* (Fig. 10c), *MYH7* (Fig. 10d) and *MYH2* (Fig. 10e). Cells were also treated with D-BAIBA (10 μM), and consistent with our in vivo study, D-BAIBA had no effect on myocyte gene expression (Fig. 10b–e). L-BAIBA-induced expression of markers of myocyte differentiation and oxidative fibers requires the cell surface G-protein coupled receptor MRGPRD in human primary myocytes.

## Discussion

Exercise training drives molecular signals to coordinate systemic physiological adaptations, which translate into health benefits across pathologies and disease risk factors^1–3^. Recent endeavours have focussed on determining the signaling processes underlying exercise adaptation as the basis for new therapeutic strategies. During exercise, skeletal muscle sits at the intersection of the mechanical processes and systemic signals that mediate adaptation^6^. PGC1α is a key transcriptional regulator of morphological and metabolic adaptation of skeletal muscle to exercise training^7,10,50^. It is now recognised that PGC1α signaling in skeletal muscle during exercise can drive the release of inter- and intra-organ signals that contribute to the molecular and physiological changes to organs and the whole body system in response to chronic exercise training^17–19,51^. There remains a key deficit in our understanding of the signaling pathways through which both PGC1α and exercise mediate the adaptive response of skeletal muscle.

Here, we propose a new paradigm in which a bioactive metabolite, BAIBA, secreted from skeletal muscle in response to exercise not only mimics the effects of exercise to increase mitochondrial metabolism, alter morphology and improve contractile function, but also contributes to both the optimal adaptation of muscle to exercise and improved systemic exercise performance in response to training. We previously established that endurance exercise and muscle PGC1α expression drives the biosynthesis and secretion of BAIBA from skeletal muscle, enrichment of BAIBA in the circulation then leads to subcutaneous adipose tissue browning^19^. The current study identified that in mice, BAIBA supplementation in drinking water increases skeletal muscle mitochondrial number and functional capacity, improved muscle contractile function and drives phenotypic remodelling similar to aerobic exercise training. We find that BAIBA supplementation in mice, when combined with exercise training through wheel running, leads to an increased adaptive response in skeletal muscle. The discovery that BAIBA not only mimics the adaptive response of muscle to exercise but also contributes to translating the benefits of exercise to improved muscle and systemic function may have important clinical implications. In our study, BAIBA treatment exhibited therapeutic potential by improving the free wheel running capacity and skeletal muscle molecular, metabolic and functional phenotype in a mouse model of obesity and T2D-induced skeletal muscle dysfunction. The L-enantiomer of BAIBA is safe, doseable and orally available in rodent models and in humans^52,53^. It may be that BAIBA can offer a novel therapy for other diseases with a skeletal muscle atrophy or dysfunction component such as chronic heart failure, cancer cachexia, or sarcopenia. Conducting intervention studies using BAIBA to target muscle function, exercise capacity and quality of life in these clinical populations will be important for future translation.

Using RNAseq, we found that BAIBA initiates a transcriptional programme related to muscle tissue development, myocyte differentiation. muscle remodelling and muscle response to activity and stretch in both murine skeletal muscle and human primary skeletal myocytes. Although suggestive of muscle regenerative pathways, we did not observe direct evidence for increased muscle regeneration following muscle injury and BAIBA treatment in vivo. However, these molecular responses translated to an initiation of a PPAR signalling transcriptional pattern and enhanced myocyte proliferation, differentiation and myonuclear fusion in response to BAIBA. The importance of PPARδ in mediating BAIBA’s effects in skeletal muscle was confirmed through knockdown of the nuclear receptor in both human skeletal myocytes in vitro and locomotor muscles in mice in vivo. Moreover, by reducing the capacity of locomotor muscle to generate L-BAIBA in mice using knockdown of *Abat*, we not only determine that it is the L-enantiomer of BAIBA that contributes to the physiological adaptive response (mitochondrial number and function, contractile capacity, fiber type remodelling) of muscle to exercise training, but also the systemic improvement in exercise performance, assessed using a maximal treadmill exercise test following treadmill exercise-training. In humans, the circulating concentration of L-BAIBA correlates significantly with aerobic exercise capacity, and is increased in response to both acute endurance exercise and endurance exercise training. Exploring BAIBA-mediated signalling mechanisms using siRNA, we found that L-BAIBA-induced expression of genes determining fiber type in HskMCs required the G-protein coupled receptor MRGPRD. Together, these data suggest the presence of an exercise-PGC1α-BAIBA-PPARδ autocrine/paracrine signalling axis as a key mechanism contributing to muscle adaptation and improved exercise performance.

Over the last several decades an endocrine role for skeletal muscle has been established with multiple myokines acting in cross tissue signalling axes to contribute to the systemic physiological regulation of exercise^17,54,55^. More recently, the view of tissue crosstalk in exercise has evolved to encompass an understanding that muscle can also secrete bioactive endocrine metabolites, known as metabokines^56–58^. This study establishes that the generation and release of a metabokine, L-BAIBA, in exercising skeletal muscle contributes to effective adaptation of muscle and subsequent improvements in exercise capacity. Our finding may provide a long sought after molecular explanation for the “cross-education effect” in skeletal muscle training. The cross-education effect is a poorly understood phenomenon in which an increase in strength or muscle function is observed in an untrained limb after training the opposite limb^59–62^. The release of L-BAIBA (and other, as yet, unidentified signals) from exercising muscle likely contribute to this phenomenon and more broadly may deliver benefits of locomotion and training to non-locomotor or inactive muscle. Our studies up to this point have focussed on aerobic exercise-training. Not all effects of BAIBA on skeletal muscle directly phenocopy endurance exercise training. In 6 week old mice treated with BAIBA or L-BAIBA for 6 weeks there is an increase in myofiber number, suggesting hyperplasia. This phenotype was recapitulated in HSkMCs treated with BAIBA and highlighted by enrichment of myogenic terms across our RNAseq datasets. Interestingly, in older, healthy mice, treated with BAIBA from 3.5 months to 7 months old (comparators for our obesity-induced skeletal muscle dysfunction model) we do not observe an increase in fiber number, with BAIBA-induced muscle remodelling characterised specifically by the more canonical exercise training-like effects of fiber, mitochondrial and functional remodelling. This likely reflects differential effects of BAIBA across the life course. Murine skeletal muscle is considered post-mitotic at 3 weeks old^63,64^. However, recent studies have suggested that a small population of satellite cells either remain non-quiescent in the skeletal muscle of young adult mice^65,66^, or can be driven out of quiescence by environmental cues^67^ or endurance exercise training^68^. Interestingly, there is also evidence that particular endurance exercise training programmes may induce hyperplasia limited to the soleus^69^. It also remains to be seen whether BAIBA may have a similar paracrine role in mediating the effects of other modes of exercise, such as through synthesis via the resistance training mediated isoform of PGC1α, PGC1α4^70^.

In summary, we find that BAIBA functions as a skeletal muscle-derived metabokine in an exercise-PGC1α-BAIBA-PPARδ axis to mediate the skeletal muscle adaptive response to exercise and improve obesity and T2D-induced muscle dysfunction, leading to enhanced exercise performance.

## Methods

### Ethical approvals

This research complies with all relevant ethical regulations. Animal studies carried out in Leeds were regulated under the Animals (Scientific Procedures) Act 1986 with procedures carried out in accordance with U.K. Home Office protocols under a U.K. Home Office Project License (PP8169223) by a U.K. Home Office Personal License Holder. Animal studies carried out at the University of Sau Paulo were approved and followed the institutional guidelines for animal care and use for research (CEUA ICB USP #2144240425). Acute human exercise studies carried out at the University of Newcastle received approval from the NHS HRA North East Tyne & Wear South Research Ethics and Newcastle University Ethics Committees (16/NE/0192, ISRCTN63739203). Human exercise training studies carried out at Wageningen University received ethical approval from the Medical Ethical Committee of Wageningen University.

### Human primary skeletal myocyte culture

Adult human skeletal myoblasts (Cell applications Inc. Cat no. 150-05a) were grown in human skeletal muscle cell growth medium (Cell applications Inc. Cat no.151-500) at 37 °C within a humidified atmosphere at 5% CO_2_. Subculture of human skeletal myoblasts occurred once 70% confluency was reached. Experiments were limited to the 5th passage. Human skeletal myoblasts were seeded at 9500 cells per cm^2^. Once confluent, myoblasts were cultured for 6–8 days in skeletal muscle differentiation media (Cell applications Inc. Cat no.151D-250) to induce myoblast differentiation to myotubes. Myoblasts were treated with either human skeletal muscle cell growth medium (Cell applications Inc.) containing BAIBA (10 μM; Sigma Aldrich, 217794) immediately after plating to assess proliferation or human skeletal muscle cell differentiation medium (Cell applications Inc.) containing BAIBA (10 µM) once confluent to assess effects of BAIBA on differentiation or human skeletal muscle cell differentiation medium (Cell applications Inc.) containing L-BAIBA (10 µM) or D-BAIBA (10 µM) (Adipogen). Media was changed every day during proliferation and every 2 days during differentiation.

The effect of BAIBA on proliferation was assessed with a non-destructive method to evaluate confluence^71^. Photographic images of HskMCs within wells were captured by photographing five areas under a standard inverted phase contrast light microscope using a digital camera with a camera lens adaptor. Images were analysed for confluence using ImageJ (1.54 f) freeware resulting in a measure of confluence known as an Area Fraction (AF). Five photographs were taken of the cells, four from the corner and one from the centre of the well. The photographs were taken consistently in the same position of the well at 24 h, 48 h and 72 h post seeding/differentiation.

Informed consent was obtained from donors. The cells were approved and complied with ethics according to:

Collection, generation, research purpose, and sale: Cell Applications, Inc. 5820 Oberlin Dr. Suite 101, San Diego, CA 92121.

Use in compliance with Human Tissue Act (UK) by Leeds Institute of Cardiovascular and Metabolic Medicine, University of Leeds, Leeds, LS2 9JT UK in 2022.

### Fusion index assay

Primary HSkMCs (Cell Applications, S150a-05a) were seeded and differentiated on collagen-coated coverslips. Myotubes were treated with either human skeletal muscle cell differentiation medium (Cell applications Inc.) containing BAIBA (10 μM; 217794, Sigma-Aldrich) or L-BAIBA (10 μM; Adipogen). Fusion was assessed at days 3 and 6 after the induction of differentiation. The cells were washed with PBS and fixed with 4% PFA/PBS for 10 minutes, followed by 7 minutes permeabilization in 0.3% TRITON, 20 minutes blocking with BSA (2%) and were incubated with primary antibodies overnight at 4 °C (monoclonal anti-myogenin antibody, clone F5D, Invitrogen catalogue no. 14-5643-82; 1:150 dilution) as a marker of differentiation, anti-myosin heavy chain antibody monoclonal MF20 Myosin heavy chain, sarcomere (MHC) (clone MF-20, Developmental Studies Hybridoma Bank catalogue no. MF20, 1;100 dilution) to visualize myotubes. The cells were then incubated with a secondary antibody (polyclonal goat anti-Mouse IgG1 Alexa Fluor 555; Thermofisher catalogue number A-21127; dilution 1:150). Slides were coverslipped in mounting medium and stained 3 min with Hoechst (20 µg/mL, Sigma) to visualize nuclei.

To establish the differentiation potential and the fusion index of the cultures, at least 1,000 nuclei from MF20-positive cells were counted from several random fields. The percentage of differentiated cells was calculated as: (nuclei within MF20-stained myocytes/total number of nuclei) × 100; or the fusion index calculated as: (MF20-stained myocytes containing ≥2 nuclei/total number of nuclei) × 100. The number of myogenin-positive nuclei was manually counted. Images were taken using a x20 objective lens using a fluorescence microscope slide scanner (Axioscan Z1, ZEISS). Images were recorded using ZEN software (Version 3.40, ZEISS).

### Seahorse bioanalyzer cellular mitochondrial respiration analysis

HSkMCs were plated at a density of 10,000 cells per well on a collagen-coated 96-well Seahorse cell culture plate and grown for 72 h in human skeletal muscle cell growth medium (Cell Applications Inc.) to assess proliferation. After 72 h, the medium was replaced with human skeletal muscle cell differentiation medium (Cell Applications Inc.) supplemented with BAIBA (10 µM; Sigma-Aldrich, cat no. 217794) to evaluate differentiation. As a negative control, the four corner wells of the plate were left cell-free and filled only with Seahorse medium (XF DMEM Medium, pH 7.4, cat no. 103575) containing 5 mM HEPES. Twelve h prior to the assay, Seahorse sensor cartridges were hydrated with Seahorse calibrant solution according to the manufacturer’s protocol and incubated in a 37 °C, CO_2_-free incubator. On the day of the assay, cells were washed and incubated with Seahorse medium. The sensor cartridge was then fitted onto the cell culture plate, which was subsequently placed in a 37 °C, CO_2_-free incubator for one hour to equilibrate. The mitochondrial stress test was performed using the Seahorse XFe96 Analyzer (Agilent) operated with Wave Controller (v. 2.6.1 Agilent). During the assay, the following inhibitors were sequentially injected according to the standard Mito Stress Test protocol: oligomycin (1 mM), FCCP (1 mM), and rotenone/antimycin A (0.5 mM). Cells were then fixed with 10% PFA and stained with Hoechst. Nuclei counts were measured using a Synergy H1 plate reader to assess fluorescence intensity and to normalize the seahorse data to cell number.

### siRNA-mediated *PPARδ* and *MRGPRD* knockdown in vitro

FlexiTube GeneSolution GS5467 siRNA against *PPARδ* (SI05383420), FlexiTube GeneSolution GS116512 siRNA against *MRGPRD* (SI00164444), AllStars negative control siRNA, and HiPerFect Transfection Reagent were purchased from Qiagen. HSkMC (Cell applications Inc. Cat no. 150-05a) transfection was performed according to the manufacturer’s instructions (75 ng siRNA, 3 μL transfection reagent per well, 60 nmol/L final siRNA concentration) on days 2 and 4 of differentiation.

### Gene expression analysis

Total RNA extraction from myocytes and skeletal muscle; cDNA conversion; and quantitative RT-PCR followed published protocols^19,42^. All data were normalized to *RPLP0* or *TBP* rRNA (mouse skeletal muscle; mouse RPLP0 primer PPM03561B-200, mouse skeletal muscle; mouse *Tbp* primer PPM03560F-200, human primary myocytes, human *RPLP0* primer PPH21138F-200, human primary myocytes, human *TBP* primer PH01091G-200, Qiagen) and quantitative measures were obtained using the ΔΔCT method. Data were analyzed using StepOne™ Software (version 2.1 Applied Biosystems). Primers are given in Supplementary Table 3.

### Animal experimentation

Six-week-old male and female C57BL6/J mice (Charles River, UK) were weight-matched and assigned to groups for treatment. Mice were treated with 100 mg/kg/day BAIBA in their drinking water for 6 weeks and fed standard chow (Special Diet Services; 801151; 3.592 kcal/g). For obesity studies six-week-old male C57BL6/J mice (Charles River, UK) were weight-matched and assigned to groups for treatment. Mice were either placed on 60% fat-diet (Bio Serv F3282; kcal 5.49 kcal/g) or standard chow (Special Diet Services; 801151; 3.592 kcal/g) for 8 weeks. After 8 weeks of the study a randomly assigned half of the 60% fat-diet group and half the standard chow fed group then began treatment with 100 mg/kg/day BAIBA for a further 14 weeks. Feeding was ad libitum.

*Abat knockdown studies:* 6 week old male C57BL6/J mice (Charles River, UK) were weight-matched and assigned to groups for treatment. Mice were either assigned to the unexercised group or to the exercised group which underwent 6 weeks of treadmill training as described below. The exercised and unexercised groups were further subdivided into groups receiving intramuscular injections into both hindlimb gastrocnemius skeletal muscles of either scrambled control Antisense LNA GapmeR (GeneGlobe nb: LG00000002, Qiagen) or Antisense LNA GapmeR against *Abat* (Abat-207_1 GeneGlobe nb: LG00838129 Sequence: CACGATAGACAAATAGA, Qiagen) at a concentration of 5 mg/Kg/injection twice a week for six weeks. GapmeRs enter cells without the need for transfection reagents and are active in vivo without the need of formulation^72–74^. Mice were fed standard chow (Special Diet Services; 801151; 3.592 kcal/g) ad libitum.

*Pparδ knockdown studies:* 6 week old male C57BL6/J mice (Charles River, UK) were weight-matched and assigned to groups for treatment. Mice were either assigned to a control group or to a group receiving 100 mg/Kg/day BAIBA in drinking water. The BAIBA-treated and control groups were further subdivided into groups receiving intramuscular injections into the hindlimb gastrocnemius skeletal muscle of either Accell non-targeting control custom siRNA **(**(Sense: 5’ U.G.G.U.U.U.A.C.A.U.G.U.C.G.A.C.U.A.A.U.U 3’; Antisense: 5’ 5’-P.U.U.A.G.U.C.G.A.C.A.U.G.U.A.A.A.C.C.A.U.U 3’) based on D-001950-01, Dharmacon™ Custom siRNA, Horizon Discovery or Accell siRNA14 against *Pparδ* ((Sense: 5’ C.U.C.C.A.A.A.U.C.U.G.A.A.A.U.G.U.A.U.U.U 3’; Antisense: 5’ 5’-P.A.U.A.C.A.U.U.U.C.A.G.A.U.U.U.G.G.A.G.U.U 3’) based on A-042751-14, Dharmacon™ Custom siRNA, Horizon Discovery) twice a week for six weeks. siRNA was delivered at 100 µg per intramuscular injection per mouse. Mice were fed standard chow (Special Diet Services; 801151; 3.592 kcal/g) ad libitum.

*D- and L-BAIBA enantiomer studies:* Six-week-old male C57BL6/J mice (Charles River, UK) were weight-matched and assigned to groups for treatment. Mice were treated with either 100 mg/kg/day D-BAIBA or L-BAIBA (Adipogen) in their drinking water for 6 weeks and fed standard chow (Special Diet Services; 801151; 3.592 kcal/g) ad libitum.

All animals were housed in conventional cages at room temperature with humidity maintained at 40–60% and a 12-h light/dark photoperiod. All studies were regulated under the Animals (Scientific Procedures) Act 1986 and complied with national and local ethical regulations for animal research. All procedures were carried out in accordance with U.K. Home Office protocols under a U.K. Home Office Project License (PP8169223) by a U.K. Home Office Personal License Holder.

*Muscle injury model:* Three-month-old male C57BL/6 J mice were obtained from the animal facility at the Institute of Biomedical Sciences, University of São Paulo, Brazil following institutional ethical approval. Mice were housed with ad libitum access to standard chow (Special Diet Services; 801151; 3.592 kcal/g) and water, maintained on a 12 h light/dark cycle at 22 ± 2 °C. Animals were randomized into two experimental groups (*n* = 7) either receiving 100 mg/kg/day BAIBA (Sigma-Aldrich) in drinking water or standard drinking water. BAIBA administration was initiated 7 days prior to injury (pre-loading phase) and continued for 14 days following cardiotoxin injury. On the day of injury, mice were anesthetized with an intraperitoneal injection of ketamine (80 mg/kg) and xylazine (10 mg/kg). Cardiotoxin (CTX; Naja mossambica) was prepared in sterile phosphate-buffered saline (PBS) and injected into the tibialis anterior (TA) muscle. A total volume of 50 µL of 10 µM CTX was administered into the mid-belly of each TA muscle using a 29-gauge insulin syringe. In all animals, the left hindlimb was subjected to cardiotoxin injury, while the right hindlimb remained uninjured. This experimental design resulted in four experimental conditions: Control (standard drinking water, right hindlimb), Injured (standard drinking water, left hindlimb), BAIBA-control (BAIBA-supplemented drinking water, right hindlimb) and BAIBA-Injured (BAIBA-supplemented drinking water, left hindlimb). Fourteen days after injury, mice were euthanized by overdose of ketamine/xylazine (ketamine 300 mg/kg and xylazine 30 mg/kg, intraperitoneally).

Reporting of animal experiments follows the ARRIVE guidelines.

### Freewheel running

Aerobic exercise training was carried out using a 6-week free wheel running protocol in male C57BL/6j mice (Charles River, UK) as previously published^19^. Mice were singly housed and given access to free wheel running (Tecniplast, Cat no.1284L0106) for 6 weeks. Controls were age-matched littermates unexercised and singly housed in matched cages.

### Treadmill training

Seven week old mice were acclimated to the treadmill apparatus (5-lane treadmill, Harvard Apparatus, Panlab) for 10 min treadmill running per day at 10 cm/s at 0% grade in the 5 days leading up to the training programme. For 6 weeks, mice ran at a speed of 25 cm/s at 10% grade during a 45-minute session per day, 4 days per week. Each session was preceded and followed by a 5-minute warm-up and cool-down at a speed of 10 cm/s respectively. After the 6 week exercise training programme a maximal running capacity test was performed, as detailed below, to identify the individual Vmax, defined as the maximum speed each mouse was capable of running voluntarily. Treadmill training was performed in groups of five mice and was voluntary. No animals refused to run.

### Maximal running test

Maximal running capacity test was performed on a motor treadmill (5-lane treadmill, Harvard Apparatus, Panlab) using a graded exercise protocol modified from previously published papers^75,76^. Briefly, mice underwent an adaptation period where the mice were placed on a stationary treadmill for 10 min per day for 5 consecutive days. On test day, mice were placed on the treadmill and exercise intensity started at 10 cm/sec and was increased for 5 cm/sec every 3 min at 0% grade until mice were able to run no longer. The time elapsed and distance run was recorded.

### Indirect calorimetry and monitored wheel running

All experiments were performed according to previously published protocols^42^. CLAMS (Columbus Instruments) was used to monitor oxygen consumption, carbon dioxide production, food intake, and voluntary wheel running using Oxymax software (version 5.37.05, Columbus Instruments). The CLAMS was calibrated before each experiment. Animals were subjected to a 3-day acclimation period in a training cage with running wheel to habituate to the environment of the metabolic cages. Animals were maintained in normal bedding at 22 °C throughout the monitoring period. Ten-minute interval measurements for each animal were obtained for oxygen and carbon dioxide with ad libitum access to food and water on a controlled 12-h light/dark cycle. Ambulatory, locomotor and wheel running activity were constantly monitored. Cages contained one mass sensor to monitor food intake. Data was collected for a 48 h period after the 3-day acclimation. Data were analyzed using CaIR (version 1.3) (https://calrapp.org/)^77^.

### Intraperitoneal glucose tolerance tests

Intraperitoneal glucose tolerance tests were performed as described^42^. Mice were fasted for 8 h with free access to water prior to baseline glucose measurements. Administration of glucose (Sigma Aldrich) was performed by intraperitoneal injection (glucose 1.5 mg/g of body weight; glucose solution 150 mg/ml). Blood was obtained from the tail vein immediately prior to glucose injection and then at 30, 60, 90, and 120 min post injection. Glucose levels were measured using a Bayer Contour Glucose Meter (Bayer Healthcare).

### Magnetic resonance imaging

Anaesthesia was induced using 5% isoflurane in 100% oxygen and then maintained using 1.5–3% isoflurane at 2 l/min oxygen flow. Animals were positioned prone on a dedicated mouse cradle. Body temperature was maintained with a custom resistive blanket placed on the back of the animal. Cardiac and respiratory signals were continuously monitored (BIOPAC Systems, Inc., Goleta, USA). Mice were imaged on a 7 T preclinical MRI scanner with a 660 mT/m shielded gradient system using either a quadrature-driven transmit/receive volume coil with inner diameter of 72 mm (Bruker BioSpin MRI GmbH, Ettlingen, Germany) or 4-element volume-array (Neos BioTec, Pamplona, Spain) operated with ParaVision software (version 6.0.1; Bruker BioSpin MRI GmbH, Ettlingen, Germany). A 2D cardiac-triggered and respiratory-gated 3-point Dixon spoiled gradient-echo sequence was used: TR = 5.65 ms, TE = 2.42/2.75/3.09 ms, matrix = 256 × 128, field-of-view = 80 × 30 mm, number of slices = 28 in sagittal orientation, slice thickness = 1 mm, number of signal averages = 8/ 1 (volume coil / volume array), total scan time ~30 min. The data were analysed in MATLAB (2022; MathWorks, Natick, USA) using the hierarchical iterative decomposition of water and fat with echo asymmetry and least squares estimation (IDEAL) method^78^. The proton density fat fraction (PDFF, the amount of lipid signal over total signal) was used to segment adipose tissue depots. Visceral adipose depots were segmented separately using Osirix Lite v11.0.2 (Bernex, Switzerland) 2D threshold region-growing algorithm tool with segmentation parameters set to a lower threshold of 80% proton density fat fraction (PDFF) i.e., a minimum of 80% of the tissue volume consisted of lipid.

### Tissue collection

Mice were killed by cervical dislocation. Blood was immediately removed via cardiac puncture and placed into EDTA-coated eppendorfs before being centrifuged at 2000 x g for 10 min. Serum was collected, frozen in liquid nitrogen, and stored at -80 °C until use. Soleus, gastrocnemius, extensor digitorum longus, and tibialis anterior muscle and inguinal and gonadal adipose tissue were removed, weighed and either used for high-resolution respirometry, contractile analysis, histology or flash-frozen in liquid nitrogen.

For the muscle injury model TA muscles were excised, trimmed, transversely sectioned, immersed in isopentane, cooled in liquid nitrogen, and stored at −80 °C.

### Immunofluorescent muscle fiber-typing

Immediately after sacrifice, the soleus muscle was embedded in OCT embedding matrix (VWR, 361603E, UK) and flash frozen in liquid nitrogen-cooled isopentane at −150 °C. Samples were stored at −80 °C until further use. Samples were sliced into 8 µm cryosections with a CM1860 Cryostat, (Leica).

Immunohistochemical staining of Myosin Heavy Chain (MHC) isoforms was performed using mouse monoclonal antibodies BA-D5 (monoclonal anti-MHC-I/ β-MHC/Myh7 specific, IgG2b, clone/catalogue no. BA-D5, 1:100 dilution), SC-71 (monoclonal anti-MHC-IIa specific, IgG1, clone/catalogue no. SC-71 1:100 dilution), BF-F3 (monoclonal anti-MHC-IIb specific, IgM, clone/catalogue no. BF-F3, 1:100 dilution), and 6H1 (monoclonal anti-MHC-IIx specific, IgM, clone/catalogue no. 6H1, 1:100 dilution) obtained from Developmental Studies Hybridoma Bank (DSHB, University of Iowa). Laminin staining was performed to label basement membranes using a rabbit polyclonal anti-laminin antibody (Sigma-Aldrich catalogue no. L9393, 1:50 dilution).

Muscle sections were washed twice (5 min each) in PBS, saturated in Blocking Buffer (FBS 4%, BSA 4%, in PBS) for 1 h, washed once in PBS, incubated with 1:100 Fab Fragment Goat Anti-Mouse IgG (Jackson ImmunoResearch Catalogue no. 115-007-003) for 20 min, then washed once in PBS. Primary antibodies were incubated overnight at 4 °C with agitation. After washing 3 times in 0.1% Tween20 in PBS, secondary antibodies were used to selectively bind to each primary antibody: polyclonal goat anti-Mouse IgG2b cross-adsorbed secondary antibody, Alexa Fluor® 647 (Invitrogen catalogue no. A-21242), polyclonal goat anti-Mouse IgG1 cross-adsorbed secondary antibody, Alexa Fluor® 350 (Invitrogen catalogue no. A21120), polyclonal goat anti-mouse IgG1 cross-adsorbed secondary antibody, Alexa Fluor® 488 (Invitrogen catalogue no. A-21121), polyclonal goat anti-mouse IgM conjugated with Alexa Fluor® 555 (Invitrogen – A-21426), polyclonal goat anti-Rabbit IgG (H + L) Highly Cross-Adsorbed Secondary Antibody, Alexa Fluor™ 488 (Invitrogen catalogue no. A-11034) all diluted at 1:400 in PBS. After three washes in 0.1% Tween20 in PBS, muscle sections were mounted using mounting media.

Images were taken using a x20 objective lens using a fluorescence microscope slide scanner (Axioscan Z1, ZEISS). Images were recorded using ZEN software (Version 3.40, ZEISS). Fiber type distribution and number were measured manually using ImageJ software.

### Histology and morphometric analysis of muscle injury model

Frozen TA muscles were sectioned at 8 µm thickness using a cryostat at −25 °C (Leica CM1850, Germany). Sections were mounted on glass slides and stained with hematoxylin and eosin (H&E). Hematoxylin was used to stain nuclei, followed by eosin counterstaining to visualize cytoplasmic and extracellular components. Images were acquired using a Zeiss Axio Imager 2 microscope with Zeiss Zen 2 Pro Software (version 2.0) (Zeiss, Germany) under brightfield illumination. Centralized nuclei were quantified using ImageJ software (National Institutes of Health, Bethesda, MD, USA). Multiple non- overlapping fields from the mid-belly region of each muscle were analyzed. For centralized nuclei data, 600 fibers were analyzed per group.

### Muscle force frequency and fatigue contraction protocols

Following cervical dislocation of the animal, the right soleus was immediately dissected and placed in a Krebs–Henseleit solution (117 mM NaCl, 4.7 mM KCl, 1.2 mM MgSO_4_, 1.2 mM KH_2_PO_4_, 24.8 mM NaHCO_3_, 2.5 mM CaCl_2_, 11.1 mM glucose). Silk sutures (4.0 - Fine Science Tools GmbH, Heidelberg, Germany) were attached to tendons at either end of the soleus and were suspended vertically in a buffer-filled organ bath between a hook and a length-controlled lever system (Aurora Scientific, Aurora, Canada). In vitro field stimulation using platinum electrodes was provided via a high-power bipolar stimulator (Aurora Scientific, Aurora, Canada) outputting supramaximal current (700 mA) operated with Dynamic Muscle Control software (version 5; Aurora Scientific, Aurora, Canada). After optimal contractile length (L_0_) was determined, the muscle was thermo-equilibrated in a Krebs–Henseleit solution for 15 minutes at 35 °C^79^.

The force-frequency relationship was then determined across stimulation frequencies of 1-150 Hz (1 s train duration; 0.25 ms pulse width, each separated by 1 minute). Following a 5 min recovery period in which muscle length was measured using digital calipers, fatigue resistance (expressed as a relative % to initial force) was further assessed across 150 repeated tetanic contractions (40 Hz, 1 s train duration, interspersed by 1 s). At the end of each experiment, Force (N) was normalized to muscle cross-sectional area (CSA; cm^2^) after dividing muscle mass (g) by the product of L_0_ (cm) and estimated muscle density (1.06 g/cm^3^) to allow specific force in N/cm^2^ to be calculated^79^.

### High-resolution respirometry

Left soleus was immediately removed, weighed, and placed into relaxing and biopsy preservation solution for high-resolution respirometry (BIOPS: 2.77 mM CaK_2_EGTA, 7.23 mM K_2_EGTA, 5.77 mM Na_2_ATP, 6.56 mM MgCl_2_·6H_2_O, 20 mM taurine, 15 mM Na_2_phosphocreatine, 20 mM imidazole, 0.5 mM Dithiothreitol (DTT), and 50 mM MES hydrate). Under a dissecting microscope (MEIJI-LABAX Co LTD, 13D46, Tokyo, Japan) individual muscle fibers from the soleus muscle were gently separated along their longitudinal axis to create a thin sheet of muscle fibers one layer thick. Separation of muscle fibers was conducted in ice-cold BIOPS on a bed of ice and was done in less than 5 minutes to ensure sample dissection consistency. Tissue was then incubated with saponin (50 μg·mL-1) to permeabilize the tissue and washed with MiR05 (MiR05: 110 mM sucrose, 60 mM K-lactobionate, 20 mM HEPES, 20 mM taurine, 10 mM KH_2_PO_4_, 3 mM MgCl_2_, 0.5 mM EGTA, 1% (w/v) fatty acid-free BSA, pH 7.1). Samples were added to the Oxygraph-2K (Oroboros Instruments, Innsbrück, Austria) which contained 2 mL MiR05, under constant stirring (750 rpm) at 37 °C.

Substrates and inhibitors were added to the chamber and steady rates of respiration recorded. Complex I was assessed with glutamate (Glu; 10 mM) and malate (Mal; 1 mM), and pyruvate (Pyr; 5 mM). ADP (2.5 mM) was titrated to provide a measure of maximal complex I-supported oxidative phosphorylation (OXPHOS). Complex I & II-supported OXPHOS was assessed with the addition of succinate (Succ; 10 mM). Carbonyl cyanide m-chlorophenyl hydrazine (CCCP; 5 μM dissolved in DMSO) yielded an uncoupled state as a measure of maximal ETC capacity. Antimycin A rates were subtracted as a correcting factor from all respiratory measurements (AA; 12.5 μM dissolved in 95 % ethanol) to account for non-mitochondrial residual respiration. Complex IV activity was assessed as a proxy for mitochondrial content. Ascorbate (Asc; 2 mM) and TMPD (N, N, N’, N’-Tetramethyl-p-phenylenediamine dihydrochloride; 0.5 mM) were added to the chambers to assess complex IV activity and Sodium Azide (AZ; 20 mM) was added to inhibit all mitochondrial respiration. Data were processed using DatLab (version 6.1, Oroboros Instruments).

Analysis of mitochondrial function in soleus muscle of HFD fed mice used a modified methodology to assess fatty acid β-oxidation as previously described^43^. OXPHOS activity was monitored using a Substrate- Uncoupler- Inhibitor-Titration (SUIT) protocol using a carnitine-conjugated long chain fatty acid palmitoyl-L-carnitine substrate (0.04 mM). All data were processed using DatLab (version 6.1, Oroboros Instruments).

### Citrate synthase assay

Citrate synthase activity was assayed according to published protocols^42^. Muscle tissue samples were homogenized in 100 mM K_2_HPO_4_/KH_2_PO_4_, 5 mM EDTA, 0.1-mM fructose-2,6-bisphosphate, 0.1% Triton X-100, and 1 mM dithiothreitol, pH 7.2. Citrate synthase activity was measured at 412 nm to detect the transfer of sulfhydryl groups to 5,5′-dithiobis(2-nitrobenzoic acid) (DTNB). Reaction buffer composition was 100 mM Tris · HCl, 0.2 mM acetyl CoA, 0.1 mM DTNB, and 1 mM oxaloacetate (omitted for control), pH 8.0. The reaction rates were linear for ≥4 min. Assays were performed in duplicate, and means were analyzed. Specific activities were expressed in international units (μmol substrate transformed to product/min) normalized to tissue weight.

### Immunoblotting

Protein lysate extracts were analysed by SDS gel electrophoresis, loading equal amounts of protein (30 μg per sample). All samples were resolved using a 4–12% Bis-Tris gradient gel (Invitrogen, Paisley UK), The proteins were transferred to 0.2 µm nitrocellulose membranes (7 × 8.5 cm) (#1704270, Bio-Rad). Total protein was visualised using Ponceau and washed off in Tris-buffered saline with 0.1% Tween 20 detergent (TBST). Membranes were blocked with skimmed milk powder for 1 h at room temperature and incubated with primary antibodies overnight at 4 °C; Total OXPHOS Rodent WB Antibody Cocktail 1:1000 (ab110413; Abcam). A cocktail of 5 mouse antibodies, one each against CI subunit NDUFB8 (ab110242; Clone 220E9DH10C12), CII-30kDa (ab14714; Clone 21A11AE7), CIII-Core protein 2 (ab14745; clone 13G12AF12BB11), CIV subunit I (ab14705, Clone 1D6E1A8) and CV alpha subunit (ab14748, Clone 15H4C4). Blots were developed with horseradish peroxidase-linked secondary antibody: m-IgG Fc BP-HRP (recombinant protein; sc-525409, Santa Cruz), 1:1000 dilution, using enhanced chemiluminescence and were detected with SuperSignal™ West Atto Ultimate Sensitivity Substrate (A38554, Thermo Fisher). Images were recorded using iBright^TM^ FL1500 imaging system (Invitrogen). Quantitative densitometry was performed using ImageJ 1.54 f software.

### Human acute endurance exercise study

Participants were aged 18–65 years, non-smokers, and free from chronic disease (except type 1 diabetes). Participants were excluded if they had diabetes-related complications, other chronic conditions, history of smoking, or BP > 140/90 mmHg at study visits. Sixty participants were enrolled in total and both male and female participants were included. Sex was established by self-report. Participant demographics are shown in Supplementary Table 1. Participants provided written informed consent prior to enrolment following approval from the NHS HRA North East Tyne & Wear South Research Ethics and Newcastle University Ethics Committees (16/NE/0192, ISRCTN63739203).

Study visit 1: Participants’ height, weight (seca 220 stadiometer/seca 889 scale; seca, Hamburg, Germany) and medical history were recorded. Participants were screened for cardiac anomalies using a modified 12-lead resting/exercising ECG. Peak oxygen uptake (VO_2PEAK_) and peak heart rate were defined using a maximal-graded walking treadmill (Valiant 2 CPET; Lode, Groningen, the Netherlands) test^80^.

Study visit 2: Participants arrived at the clinical research facility at 08:30 A.M. following an overnight fast, had abstained from exercise for 48 h. Participants were cannulated and resting (baseline) blood samples (10 ml) were drawn. Participants walked on an incline for 45 minutes at 60% VO_2PEAK_ (Valiant 2 CPET; Lode, Groningen, the Netherlands) operated with Lode Ergometry Manager software (version 9; Lode, Groningen, the Netherlands). Participants’ treadmill velocity and gradient were calculated using VO_2_, velocity, and gradient data from the preliminary VO_2PEAK_ test. Breath-by-breath respiratory parameters (Metalyzer 3B-R3, Cortex) were recorded, with gradient adjusted at 10 and 30 minutes if VO_2_ was >10% different than target VO_2_. On exercise completion blood was immediately drawn from the cannula. Blood was processed for serum which was stored at - 80 °C.

### Human aerobic exercise training study

The study received ethical approval from the Medical Ethical Committee of Wageningen University, in accordance with the Declaration of Helsinki. For the present analysis a random sub-set of the serum from thirty-three participants were used. A detailed description of subject participation, experimental design, endurance training program, whole-body physiological outcome measures can be found in previous publications^47^. Demographics of the patients used in this study can be found in Supplementary Table 2.

Healthy male volunteers gave full written informed consent to participate in a 10-week aerobic exercise training program. Sex was established by self-reporting. All subjects were physically active, performing sports on a non-competitive basis between 1 and 4 h/week. The total study duration was 10 weeks, and included 28 endurance training sessions. Training sessions involved a 10-min warmup on a cycle ergometer, followed by the endurance training session of 60-min continuous cycling. All training sessions were conducted under the supervision of a researcher using indoor, mechanically braked spinning bikes (Body Bike Smart, Body Bike International). Heart rate (HR) for each session was determined (Polar Electro), HR and rate of perceived exertion were assessed at start and every 5 min. After the endurance training sessions participants performed a 10-min cooling down period on the same cycle ergometer. Exercise intensity was determined using published approaches^81^. The exercise intensity is “vigorous”^82^. Fasting blood was collected in EDTA-coated evacuated tubes (BD Biosciences) by venepuncture at baseline and study end. Fasting blood samples were collected after an overnight fast 3–4 days before the first training session (baseline), and following the 28th training session (Exercise trained).

### LC-MS analysis of total BAIBA

LC-MS analysis of plasma and serum BAIBA was conducted following published methods^19,83^. Plasma samples were prepared for LC-MS analyses via protein precipitation with the addition of nine volumes of 74.9:24.9:0.2 vol/vol/vol acetonitrile/methanol/formic acid. The samples were centrifuged (10 min, 15,000 *g*, 4 °C) An Acquity UPLC system (Waters, USA) equipped with an Atlantis HILIC silica column (3 µm, 2.1 × 150 mm) held at 30 ^◦^C was used for the analysis of BAIBA and operated with MassLynx software (version 4.1; Waters, USA). The binary solvent system was solvent A comprising LC − MS-grade water, 0.1% formic acid, 10 mM ammonium formate and solvent B comprising 0.1% formic acid in acetonitrile.

Elution Gradient: 95% B held for 0.5 min at a flow rate of 250 μl/min, decreasing to 40% B over 10 mins at 250 μl/min with a hold of 40%B for 5 min at 250 μl/min. Then a return to 95% B for 2 min at 250 μl/min followed by an increase to 400 μl/min for 12.5 min. Flow was then returned to 250 μl/min over 1 minute and held for 0.5 min. Total runtime was 32 minutes.

The Acquity UPLC system was coupled to a Xevo TQ-XS mass spectrometer (Waters). Positive electrospray ionisation mode, a cone gas flow rate of 50 ml/h, and a desolvation temperature of 650 °C were used. BAIBA analyses were performed using multiple reaction monitoring (MRM) with a parent ion (*m/z*) in Q1 of 104.1, a fragment ion (*m/z*) in Q2 of 86 and a collision energy of 17. Samples were analysed in technical triplicate. For data related to Supplementary Fig. 1b, 23 independent samples were analysed. For data related to Supplementary Fig. 3a, 8 independent samples were analysed. For data related to Supplementary Fig. 5d, 40 independent samples were analysed. For data related to Supplementary Fig. 6a, 16 independent samples were analysed.

Data were processed and peak integration performed using Waters TargetLynx Version 4.1 (Waters, USA).

### LC-MS analysis of D-BAIBA and L-BAIBA enantiomers

LC-MS analysis of D-BAIBA and L-BAIBA was based on published methods^84^. An internal standard spiking solution of 10 μM D,L-BAIBA-d3 (CDN Isotopes) in 0.1% formic acid in LC-MS-grade methanol was prepared. Serum or plasma (10 µl) was protein-precipitated with 40 µl internal standard spiking solution, vortex mixed and centrifuged (15 min; 13,000 g; 4 °C). The supernatant fraction was transferred to LC vials. Supernatant (10 µl) was injected on to an Acquity UPLC system (Waters, USA) equipped with a chiral SPP-TeicoShell, 150 × 4.6 mm, 2.7 µm column (AZYP LLC., Arlington, TX) with a Max-RP 50 × 2.0 mm guard column (Phenomenex, Torrance, CA) and held at room temperature. The binary solvent system used for the analysis of the BAIBA enantiomers was solvent A comprising LC − MS-grade methanol and solvent B comprising 0.005% formic acid and 2.5 mM ammonium formate in LC-MS-grade water. The mobile phase was set at a flow rate of 0.6 mL/min. The column mobile phase was held at 25% solvent B for 0–10 min before increasing to 98% solvent B over 10–10.1 min. The mobile phase was then held at 98% B from 10.1–17 min before returning to 25% B 17.1-25 min. The Acquity UPLC system was coupled to a Xevo TQ-XS mass spectrometer (Waters) and operated with MassLynx software (version 4.1; Waters, USA. Analyses were performed using multiple reaction monitoring (L-BAIBA and D-BAIBA 104.1 *m/z* → 86 *m/z*, cone voltage = 12 V, collision energy = 12 eV) (Supplementary Fig. 11). Positive electrospray ionisation was used. A cone gas flow rate of 50 ml/h and a desolvation temperature of 650 °C were used. Samples were analysed in technical triplicate. For data related to Fig. 7a, b, 24 independent samples were analysed. For data related to Fig. 8a–d and Supplementary Fig. 9a–f, 60 independent samples were analysed. For data related to Fig. 8e–g, 66 independent samples were analysed. For data related to Fig. 9c and Supplementary Fig 10c, 40 independent samples were analysed. Data were processed and peak integration performed using Waters TargetLynx Version 4.1 (Waters, USA).

### RNAseq analysis

All RNA samples were quality checked on the Tapestation 4200 operated with TapeStation Analysis Software (version 3; Agilent), using the RNA tapes (Agilent), as well as the Quant-iT RNA kit (Invitrogen). 200 ng of total RNA was taken forward, and libraries prepared using the Illumina Stranded mRNA library preparation kit (Illumina) using their standard operating procedure. 12 cycles were used for the enrichment PCR. Libraries were quality checked on the Qubit BR dsDNA kit (Invitrogen) and Tapestation 4200 with D1000 tapes (Agilent). Any libraries that still had visible adapter dimer had an additional bead clean up to remove adapter dimer. Libraries were pooled at 23 ng each, and sequenced on the Illumina NextSeq2000 using the P2 100 cycle SBS kit in a single end sequencing run. Data was converted using Illumina’s BCL_Convert off instrument.

Raw FASTQ files were trimmed using TrimGalore to remove low-quality reads and overrepresented sequences. A reference mouse genome was retrieved from the Ensembl genome database (GRCm39). STAR aligner was then used to align the trimmed sequences to the reference human genome. Once aligned, raw read counts were calculated using FeatureCounts in R. DEGs were identified using DESeq2 package (v1.40). Gene ontology enrichment was performed using ClusterProfileR (v4.14.4). Gene Set Enrichment Analysis was performed using Enrichr (2021)^28^ and the MSigDB Molecular Signatures Database^29^. For all RNA-seq analyses, adjusted *p* value < 0.05 was set as a threshold for statistical significance. The R version used for the analysis was v4.3.0. The raw data of the RNAseq data was deposited in the EBI ArrayExpress repository (https://www.ebi.ac.uk/biostudies/arrayexpress) can be accessed via https://www.ebi.ac.uk/arrayexpress/experiments/E-MTAB-14746 (human skeletal muscle cell datasets) and https://www.ebi.ac.uk/arrayexpress/experiments/E-MTAB-14747 (murine soleus datasets).

### Statistical analysis

Sample sizes were calculated using power calculations. Animals/cell culture wells were randomly assigned to experimental groups. Group variance was analyzed with an F test. Statistical significance was assessed using one-way or two-way ANOVA (Tukey’s, Holm–Sidak’s, Fisher’s and Dunnett’s post hoc test), or two-tailed unpaired Student’s t test, as detailed. In each case, *n*  ≥  3 and represents independent biological repeats. The significance level was set to *p*  ≤  0.05 or adjusted *p*  ≤  0.05 (FDR; RNAseq data). Univariate analysis was conducted using GraphPad Prism (version 10) software. Differential expression analysis statistics were conducted in DESeq2 (v1.4) (Bioconductor). Indirect calorimetry data were analyzed, and *p* values were calculated using ANCOVA/Generalized Linear Model with body mass as a covariate in CaIR (version 1.3, https://calrapp.org/)^77^.

### Reporting summary

Further information on research design is available in the Nature Portfolio Reporting Summary linked to this article.

## Supplementary information

Supplementary Information

Reporting Summary

Transparent Peer Review file

## Source data

Source Data

## Data Availability

RNA-Seq data associated with this study are available from EBI ArrayExpress repository (https://www.ebi.ac.uk/biostudies/arrayexpress) can be accessed via https://www.ebi.ac.uk/arrayexpress/experiments/E-MTAB-14746 (human skeletal muscle cell datasets; Accession number E-MTAB-14746) and https://www.ebi.ac.uk/arrayexpress/experiments/E-MTAB-14747 (murine soleus datasets; Accession number E-MTAB-14747). Other data generated in this study are provided in the Supplementary Source Data file. Source data are provided with this paper.
