# Supplementary material for: The metabokine β-aminoisobutyric acid mediates exercise performance and skeletal muscle adaptation through a PGC1α-BAIBA-PPARδ axis
Source: Nat Commun. 2026 Aug 18;17:8090. doi: 10.1038/s41467-026-76307-8 (PMC13487176; doi:10.1038/s41467-026-76307-8)
Supplement: Supplementary file 1 [file 41467_2026_76307_MOESM1_ESM.pdf]

Supplementary Information

The metabokine β-aminoisobutyric acid mediates exercise performance and skeletal muscle adaptation through a PGC1α-BAIBA-PPARδ axis

Helene N Daou[1,2], Nicole T Watt[1], Shaimaa A Gad[1,3], Amanda DV MacCannell[1], Aysha Ali[1], Chew Cheng[1], Raquel L Fernando[1], Aysha Ali[1], T. Simon Futers[1], Sumeet A. Khetarpal[4], Harrison Gallagher[2], Luis D Chinait[5], Anselmo S Moriscot[5], Pim Knuiman[6], Koen Manusama[6], Marco Mensink[6], Irvin Teh[1], Jurgen E Schneider[1], Richard Cubbon[1], Guy S Taylor[7], Daniel J West[7], David J Beech[1], T Scott Bowen[2], Lee D Roberts[1*]

1. Leeds Institute of Cardiovascular and Metabolic Medicine, School of Medicine, University of Leeds, Leeds, LS2 9JT, UK.

2. Faculty of Biological Sciences, University of Leeds, LS2 9JT, UK.

3. Faculty of Medicine, Mansoura University, Egypt.

4. Dana-Farber Cancer Institute and Harvard Medical School, Boston, MA, 02215, USA

5. Institute of Biomedical Sciences, University of Sao Paulo, Brazil

6. Division of Human Nutrition, Wageningen University and Research, Wageningen, Netherlands.

7. Human Nutrition & Exercise Research Centre, Population Health Sciences Institute, Newcastle University, Newcastle upon Tyne, UK.

Supplementary Tables

**Characteristic**	
N	60
No. male/female	32/28
Age, years	37.9 ± 12
BMI, kg/m^2	25 ± 4.2
VO_{2PEAK} $ml\ kg^{-1}\ min^{-1}$	40.6 ± 11

Supplementary Table 1

Acute exercise study participant demographic data. Data are presented as mean ± SD. Source data are provided as a Source Data file.

**Characteristic**	**Baseline**	**Exercise Trained**	***P* value**
N	33	33	-
Age, years	22.2 ± 2.18	-	-
BMI, kg/m^2	22.25 ± 1.46	22.35 ± 1.53	0.123
Body weight, kg	75.61 ± 5.9	75.94 ± 6.1	0.127
VO_{2PEAK} ml kg^{-1} min^{-1}	50.31	54.26	0.000002

Supplementary Table 2

Endurance exercise training study participant demographic data. Data are presented as mean ± SD. Paired Two-tailed Student’s T-Test. Source data are provided as a Source Data file.

**Species**	**Gene**	**Commercial Vendor**	**Catalogue Number**	**Primer Reference Position**
**Human**	*PAX7*	Qiagen (GeneGlobe)	PPH06872C	1305 (NM_002584)
	MYOD1		PPH00046A	371 (NM_002478)
	MYF5		PPH01625A	170 (NM_005593)
	MYF6		PPH09037B	498 (NM_002469)
	CPT1b		PPH20905B	23 (NM_004377)
	MYH2		PPH23817B	4051 (NM_017534)
	MYH7		PPH00044E	1922 (NM_000257)
	PPARD		PPH00455A	1247 (NM_006238)
	RPLP0		PPH21138F	921 (NM_001002)
	TBP		PPH01091G	701 (NM_003194)
	MRGPRD		PPH23882A	966 (NM_198923)
**Mouse**	*Trim63*		PPM61645B	1531 (NM_001039048)
	Fbxo32		PPM38061A	1142 (NM_026346)
	Myog		PPM04482A	674 (NM_031189)
	Myh2		PPM28209A	4523 (NM_001039545)
	Myh7		PPM67019B	5319 (NM_080728)
	Abat		PPM35621B	1688 (NM_172961)
	Ppard		PPM03365F	1182 (NM_011145)
	Rplp0		PPM03561B	1086 (NM_007475)
	Tbp		PPM03560F	1515 (NM_013684)

Supplementary Table 3 PCR primer details giving species, gene, commercial vendor, vendor catalogue number and primer reference position in NCBI reference sequence.

Figures

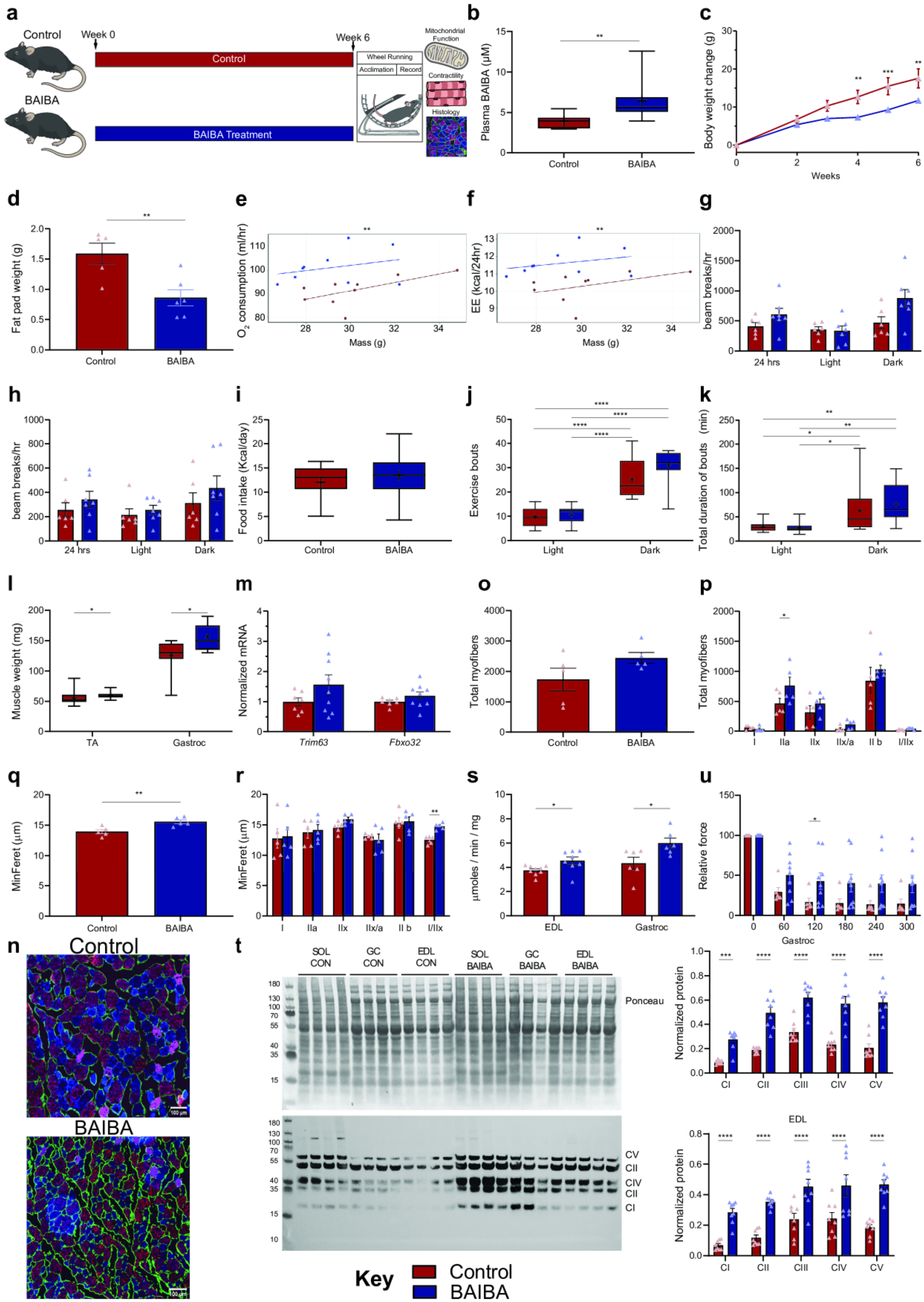

a
Control
Week 0
Week 6
Control
BAIBA
BAIBA Treatment
Wheel Running
Acclimation
Record
Mitochondrial Function
Contractility
Histology
b
Plasma BAIBA (μM)
Control
BAIBA
c
Body weight change (g)
Weeks
d
Fat pad weight (g)
Control
BAIBA
e
O2 consumption (ml/hr)
Mass (g)
f
EE (kcal/24hr)
Mass (g)
g
beam breaks/hr
24 hrs
Light
Dark
h
beam breaks/hr
24 hrs
Light
Dark
i
Food intake (Kcal/day)
Control
BAIBA
j
Exercise bouts
Light
Dark
k
Total duration of bouts (min)
Light
Dark
l
Muscle weight (mg)
TA
Gastroc
m
Normalized mRNA
Trim63
Fbxo32
o
Total myofibers
Control
BAIBA
p
Total myofibers
I
IIa
IIx
IIx/a
II b
I/IIx
q
MinFeret (μm)
Control
BAIBA
r
MinFeret (μm)
I
IIa
IIx
IIx/a
II b
I/IIx
s
μmoles / min / mg
EDL
Gastroc
u
Relative force
Gastroc
n
Control
BAIBA
t
SOL CON
GC CON
EDL CON
SOL BAIBA
GC BAIBA
EDL BAIBA
Ponceau
CV
CIII
CIV
CII
CI
Normalized protein
CI
CII
CIII
CIV
CV
EDL
Normalized protein
Key
Control
BAIBA

Supplementary Figure 1. BAIBA induces exercise training-like adaptive effects in vivo. (**a**) Experimental schematic of mice treated with 100 mg/kg/day BAIBA for 6 weeks with metabolic and wheel running phenotyping and key muscle endpoints including contractility, high-resolution respirometry and histology. This schematic contains images provided by Servier Medical Art (https://smart.servier.com), licensed under CC BY 4.0 (https://creativecommons.org/licenses/by/4.0/). (**b**) β-aminoisobutyric acid (BAIBA) plasma concentration in mice receiving BAIBA compared to control mice (Control n = 11, BAIBA n = 12; $p = 0.004$ two-tailed t-test). (**c**) Weight gain of mice receiving BAIBA compared to untreated controls (Control n = 14, BAIBA = 20; two-way ANOVA; week 4 $p = 0.005$, week 5 $p = 0.0007$, week 6 $p = 0.0017$). (**d**) Total inguinal and visceral fat pad weight of mice receiving BAIBA compared to untreated controls (Control n = 5, BAIBA n = 6; $p = 0.008$; two-tailed t-test). (**e**) Oxygen consumption against body mass of mice receiving BAIBA compared to untreated controls (Control n = 9, BAIBA n = 9; $p = 0.002$; ANCOVA). (**f**) Energy expenditure against body mass of mice receiving BAIBA compared to untreated controls (Control n = 9, BAIBA n = 9; $p = 0.0014$; ANCOVA). (**g**) Locomotor activity of mice receiving BAIBA (24 hr diurnal light and dark phases) compared to untreated controls (Control n = 6, BAIBA n = 7). (**h**) Ambulatory activity of mice receiving BAIBA (24 hr diurnal light and dark phases) compared to untreated controls (Control n = 6, BAIBA n = 7). (**i**) 24 hr food intake of mice receiving compared to untreated controls (Control n = 8, BAIBA n = 8). (**j**) Average number of exercise bouts of mice receiving BAIBA (24 hr diurnal light and dark phases) compared to untreated controls (Control n = 10, BAIBA n = 11; Control Light vs Control Dark $p < 0.0001$, Control Light vs BAIBA Dark $p < 0.0001$, BAIBA Light vs BAIBA Dark $p < 0.0001$, BAIBA Light vs Control Dark $p < 0.0001$; Two-way ANOVA). (**k**) Total duration in minutes of exercise bouts of mice receiving BAIBA (24 hr diurnal light and dark phases) compared to untreated controls (Control n = 10, BAIBA n = 11; Control Light vs Control Dark $p = 0.03$, Control Light vs BAIBA Dark $p = 0.0025$, BAIBA Light vs BAIBA Dark $p = 0.0014$, BAIBA Light vs Control Dark $p = 0.02$; Two-way ANOVA). (**l**) Tibialis anterior (TA) and gastrocnemius (Gastroc) weight of mice receiving BAIBA compared to untreated controls (TA Control n = 32, BAIBA n = 29; $p = 0.04$; Gastroc n = 9, $p = 0.02$ two-tailed t-test). (**m**) Soleus *Tripartite Motif Containing 63* (*Trim63*) and *Atrogin-1* (*Fbxo32*) expression of mice receiving BAIBA compared to untreated controls (Control n = 7, BAIBA n = 9). (**n**) Cross sectional confocal images of gastrocnemius muscle from mice receiving BAIBA compared to untreated controls. Myosin heavy chain I (MyHCI; green), MyHCIIa (blue), MyHCIIb (red), nuclei and the basal lamina (laminin; green). Scale bar = 100 μm. (**o**) Gastrocnemius total myofibers from mice receiving BAIBA compared to untreated controls (n = 5). (**p**) Gastrocnemius total myofibers by fibertype (I, IIa, IIx, IIb and hybrid IIx/a and I/IIx) from mice receiving BAIBA compared to untreated controls (n = 5; IIa $p = 0.03$). (**q**) Gastrocnemius fibers MinFeret (μm) diameter from mice receiving BAIBA compared to

untreated controls (Control n = 5 BAIBA n = 5); $p = 0.008$; two-tailed t-test). (**r**) Gastrocnemius fiber MinFeret (μm) diameter by fibertype (I, IIa, IIx, IIb and hybrid IIx/a and I/IIx) from mice receiving BAIBA compared to untreated controls (n = 5; I/IIx $p = 0.002$). (**s**) Extensor digitorum longus (EDL) and gastrocnemius citrate synthase activity of mice receiving BAIBA compared to untreated controls (EDL Control n = 9, BAIBA n = 8, $p = 0.02$; Gastroc n = 6, p = 0.027; two-tailed t-test). (**t**) Representative immunoblot and quantitation of soleus, gastrocnemius and EDL mitochondrial respiratory complexes I-V of mice receiving BAIBA compared to untreated controls (Gastroc n = 8, CI $p = 0.0005$, CII-CV $p < 0.0001$; EDL n = 8, CII-CV $p < 0.0001$; Two-way ANOVA) (n = 3 repeats). (**u**) EDL relative specific force over 300 s of mice receiving BAIBA compared to untreated controls (control = 6, BAIBA = 8; 120s $p = 0.04$). Red = Control; Blue = BAIBA. Data in bar charts are mean ± SEM with data points shown. Box and whisker plots show 25th to 75th percentile (box) min to max (whiskers), mean (+) and median (−).* p ≤ 0.05, ** p < 0.01, *** p < 0.001. Source data are provided as a Source Data file.

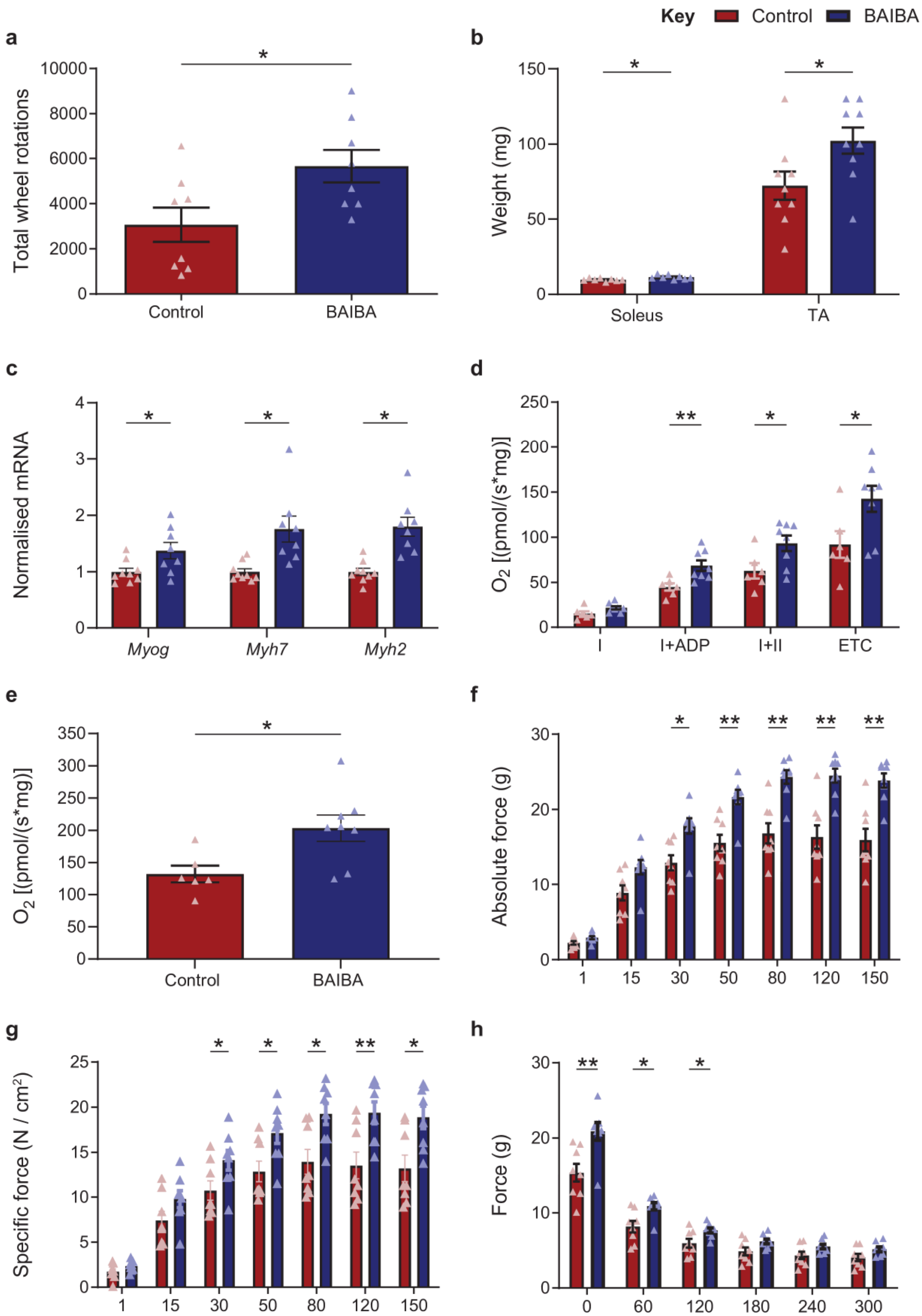

Key
Control
BAIBA
a
Total wheel rotations
10000
8000
6000
4000
2000
0
*
Control
BAIBA
b
Weight (mg)
150
100
50
0
*
*
Soleus
TA
c
Normalised mRNA
4
3
2
1
0
*
*
*
Myog
Myh7
Myh2
d
O2 [(pmol/(s*mg)]
250
200
150
100
50
0
**
*
*
I
I+ADP
I+II
ETC
e
O2 [(pmol/(s*mg)]
350
300
250
200
150
100
50
0
*
Control
BAIBA
f
Absolute force (g)
30
20
10
0
*
**
**
**
**
1
15
30
50
80
120
150
g
Specific force (N / cm2)
25
20
15
10
5
0
*
*
*
**
*
1
15
30
50
80
120
150
h
Force (g)
30
20
10
0
**
*
*
0
60
120
180
240
300

Supplementary Figure 2. BAIBA induces an exercise training-like effect on exercise performance and muscle function in female mice in vivo. (**a**)**.** Total running wheel rotations over a 24hr diurnal cycle in female mice receiving BAIBA compared to untreated controls (n = 8, *p* = 0.027; two-tailed t-test). (**b**) Soleus and tibialis anterior (TA) weight of female mice receiving BAIBA compared to untreated controls (Soleus n = 8, *p* = 0.015; TA n = 9, *p* = 0.03; two-tailed t-test). (**c**) Soleus *Myogenin* (*Myog*), *Myosin heavy chain 7* (*Myh7*) and *Myh2* expression from female mice receiving BAIBA compared to untreated controls (Control n = 9; BAIBA n = 8; *Myog* p = 0.03, *Myh7* p = 0.004, *Myh2* p = 0.0003; two-tailed t-test). (**d**) Soleus high-resolution respirometry analysis from female mice receiving BAIBA compared to untreated controls assessed for complex I (I), complex I-supported oxidative phosphorylation (I + ADP), complex I & II-supported oxidative phosphorylation (I + II) and maximal uncoupled substrate oxidation (ETC), corrected for tissue mass (control = 6, BAIBA = 8; I + ADP *p* = 0.007, I + II *p* = 0.03, ETC *p* = 0.03; two-way ANOVA). (**e**) Soleus high-resolution respirometry complex IV activity from female mice receiving BAIBA compared to untreated controls (control = 6, BAIBA = 8; p = 0.02; two-tailed t-test). (**f**) Soleus absolute ex vivo contractile force from female mice receiving BAIBA compared to untreated controls (n = 8; 30 Hz *p* = 0.029 50 Hz *p* = 0.006; 80 Hz *p* = 0.004; 120 Hz *p* = 0.005, 150 Hz *p* = 0.006; two-way ANOVA). (**g**) Soleus specific ex vivo contractile force (N/cm2) from female mice receiving BAIBA compared to untreated controls (n = 8; 30 Hz *p* = 0.04 50 Hz *p* = 0.02; 80 Hz *p* = 0.011; 120 Hz *p* = 0.009, 150 Hz *p* = 0.01; two-way ANOVA). (**h**) Soleus absolute force over 300 s in female mice receiving BAIBA compared to untreated controls (n = 8; 0s *p* = 0.005, 60s *p* = 0.012; 120s *p* = 0.03). Data in bar charts are mean ± SEM with data points shown. Control = red, BAIBA = blue. * p ≤ 0.05, ** p < 0.01. Source data are provided as a Source Data file.

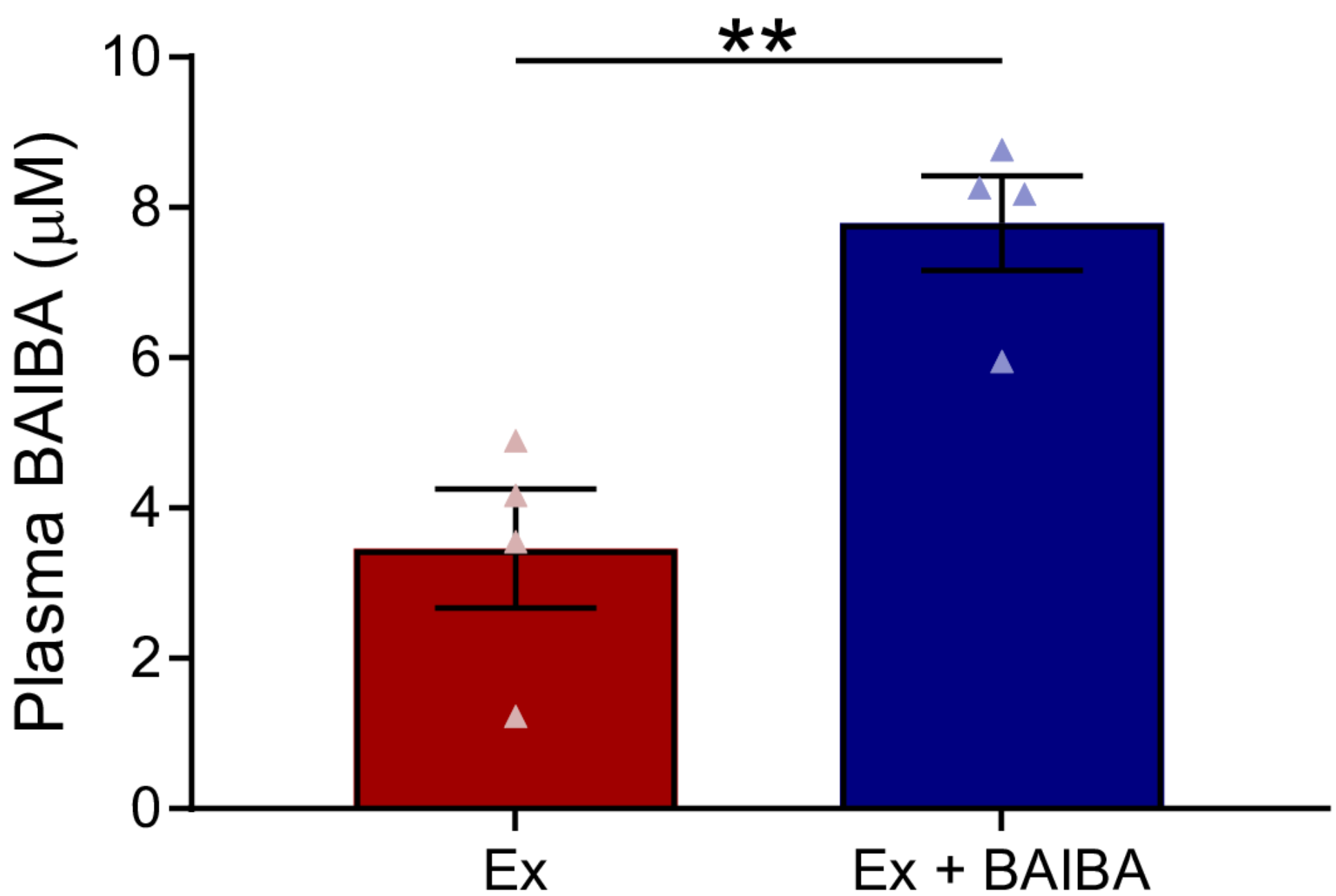

Supplementary Figure 3. BAIBA supplementation is additive to exercise-induced BAIBA plasma concentration in vivo. (**a**) Plasma BAIBA concentration in male mice following 6 weeks of free wheel running exercise (Ex) either with or without 100 mg/Kg/day BAIBA treatment (n = 4; p = 0.005; two-tailed Student's t-test). Data in bar charts are mean ± SEM with data points shown. Control = red, BAIBA = blue. ** $p < 0.01$. Source data are provided as a Source Data file.

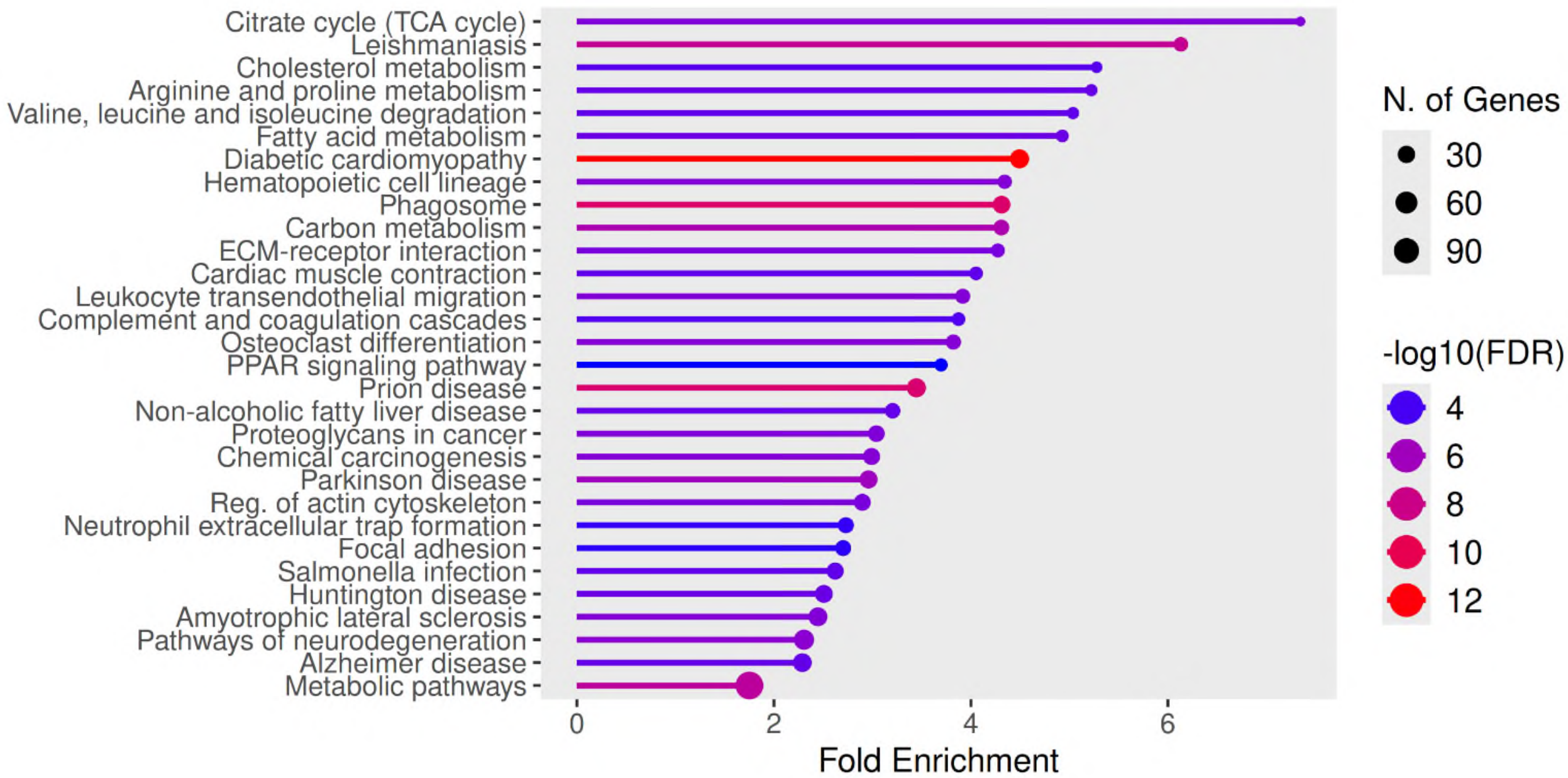

Supplementary Figure 4. Gene Set Enrichment Analysis of the RNAseq data obtained from the soleus of control and BAIBA-treated (100 mg/Kg/day; 6 weeks) mice (n = 3) generated using ShinyGO and the KEGG Database (*p*-value adjusted for false discovery rate). Source data is available at https://www.ebi.ac.uk/arrayexpress/experiments/E-MTAB-14747.

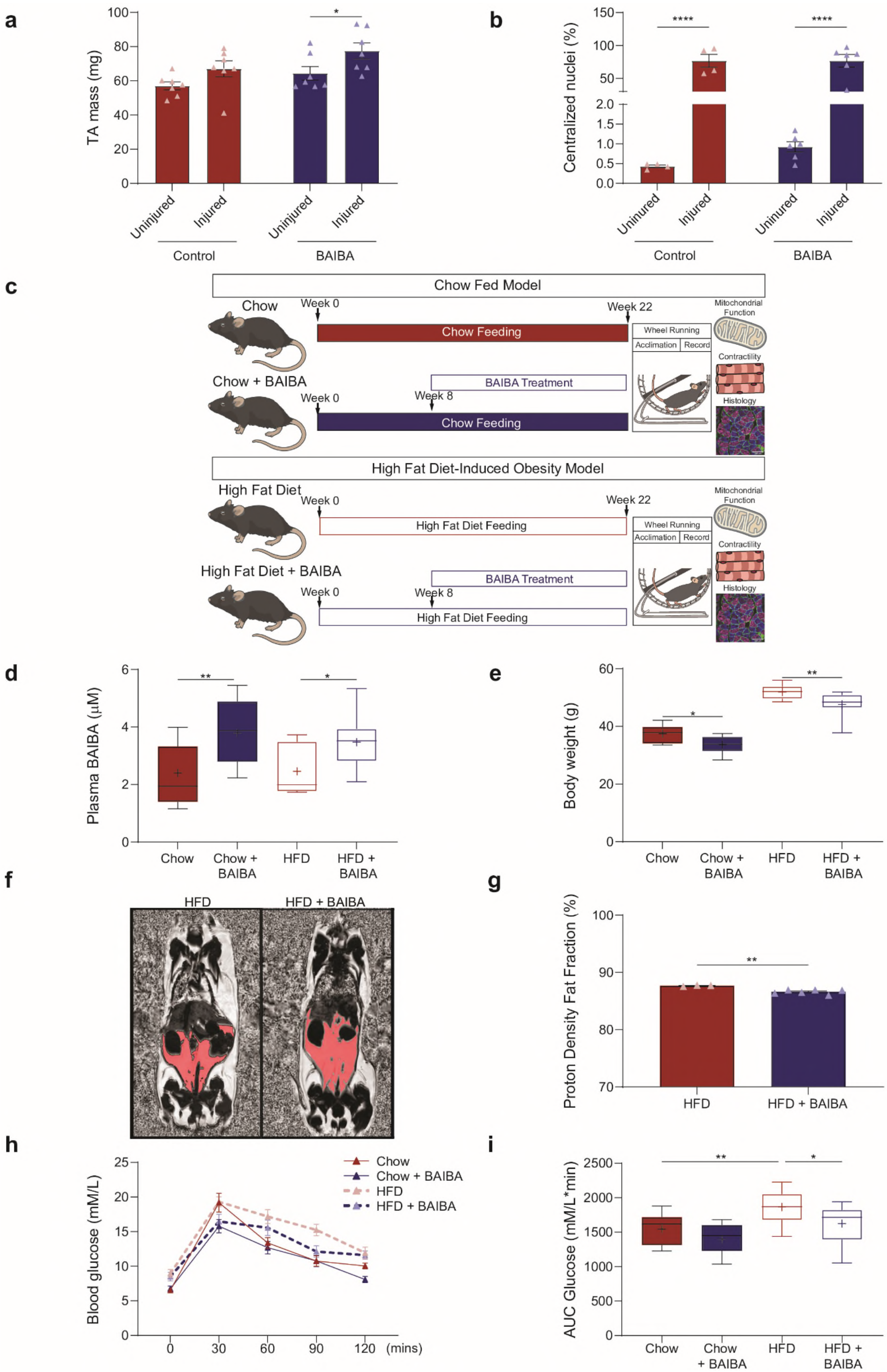
a
TA mass (mg)
Uninjured
Injured
Control
BAIBA
b
Centralized nuclei (%)
Control
BAIBA
c
Chow Fed Model
Chow
Week 0
Week 22
Chow Feeding
Chow + BAIBA
BAIBA Treatment
Week 8
Wheel Running
Acclimation
Record
Mitochondrial Function
Contractility
Histology
High Fat Diet-Induced Obesity Model
High Fat Diet
High Fat Diet Feeding
High Fat Diet + BAIBA
d
Plasma BAIBA (µM)
Chow
Chow + BAIBA
HFD
HFD + BAIBA
e
Body weight (g)
f
HFD
HFD + BAIBA
g
Proton Density Fat Fraction (%)
h
Blood glucose (mM/L)
(mins)
Chow
Chow + BAIBA
HFD
HFD + BAIBA
i
AUC Glucose (mM/L*min)

Supplementary Figure 5. The effect of BAIBA in a muscle injury model and obesity-induced muscle dysfunction in mice. (**a**) Tibialis anterior (TA) weight in control and BAIBA-treated mice undergoing contra-lateral cardiotoxin-induced injury compared to the uninjured leg (n = 7; BAIBA Uninjured vs Injured $p = 0.029$; Injury effect $p = 0.008$; BAIBA effect $p = 0.035$; Two-way ANOVA). (**b**) TA centralized nuclei (%) of control and BAIBA-treated mice undergoing contra-lateral cardiotoxin-induced injury compared to the uninjured leg (Control n = 4, BAIBA n = 6; Control Uninjured vs Injured $p < 0.0001$; BAIBA Uninjured vs Injured $p < 0.0001$). (**c**) Schematic of mice fed either a chow or 60% high fat diet (HFD) for 8 weeks before subcategorization with half the chow and HFD-fed mice given 100 mg/kg/day BAIBA for a further 14 weeks followed by metabolic and wheel running phenotyping and key muscle endpoints including contractility, respirometry and histology. This schematic contains images provided by Servier Medical Art (https://smart.servier.com), licensed under CC BY 4.0 (https://creativecommons.org/licenses/by/4.0/). (**d**) β-aminoisobutyric acid (BAIBA) plasma concentration in mice fed either a chow diet or high fat diet (HFD) with and without BAIBA treatment (Chow n = 10, Chow + BAIBA n = 10, HFD n = 10, HFD + BAIBA n = 10; Chow vs Chow + BAIBA $p = 0.006$, HFD vs HFD + BAIBA $p = 0.039$; One-way ANOVA). (**e**) Final body weights of mice fed a chow diet or 60% HFD with and without BAIBA treatment (n = 10; Chow vs Chow + BAIBA $p = 0.013$, HFD vs HFD + BAIBA $p = 0.0058$; One-way ANOVA). (**f**) Representative fat:water MRI from mice fed a 60% HFD with and without BAIBA treatment. (**g**) Proton density visceral fat fraction from mice fed HFD with and without BAIBA treatment (HFD n = 3, HFD + BAIBA n = 6; $p = 0.0024$; two-tailed t-test). (**h**) Glucose tolerance test in mice fed either a chow diet or HFD with and without BAIBA treatment (Chow n = 9, Chow + BAIBA n = 9, HFD n = 10, HFD + BAIBA n = 10; Chow vs HFD $p < 0.0001$, HFD vs HFD + BAIBA $p = 0.006$; two-way ANOVA). (**i**) Area under the curve of the glucose tolerance test of mice fed a chow diet or HFD with and without BAIBA treatment (Chow n = 9, Chow + BAIBA n = 9, HFD n = 10, HFD + BAIBA n = 10; Chow vs HFD $p = 0.009$, HFD vs HFD + BAIBA p =0.038; One-way ANOVA). Data in bar charts are mean ± SEM with data points shown. Box and whisker plots show 25th to 75th percentile (box) min to max (whiskers), mean (+) and median (−). Chow = red, Chow + BAIBA = blue, HFD = red border; HFD + BAIBA = blue border. * $p \leq 0.05$, ** $p < 0.01$, **** $p < 0.0001$. Source data are provided as a Source Data file.

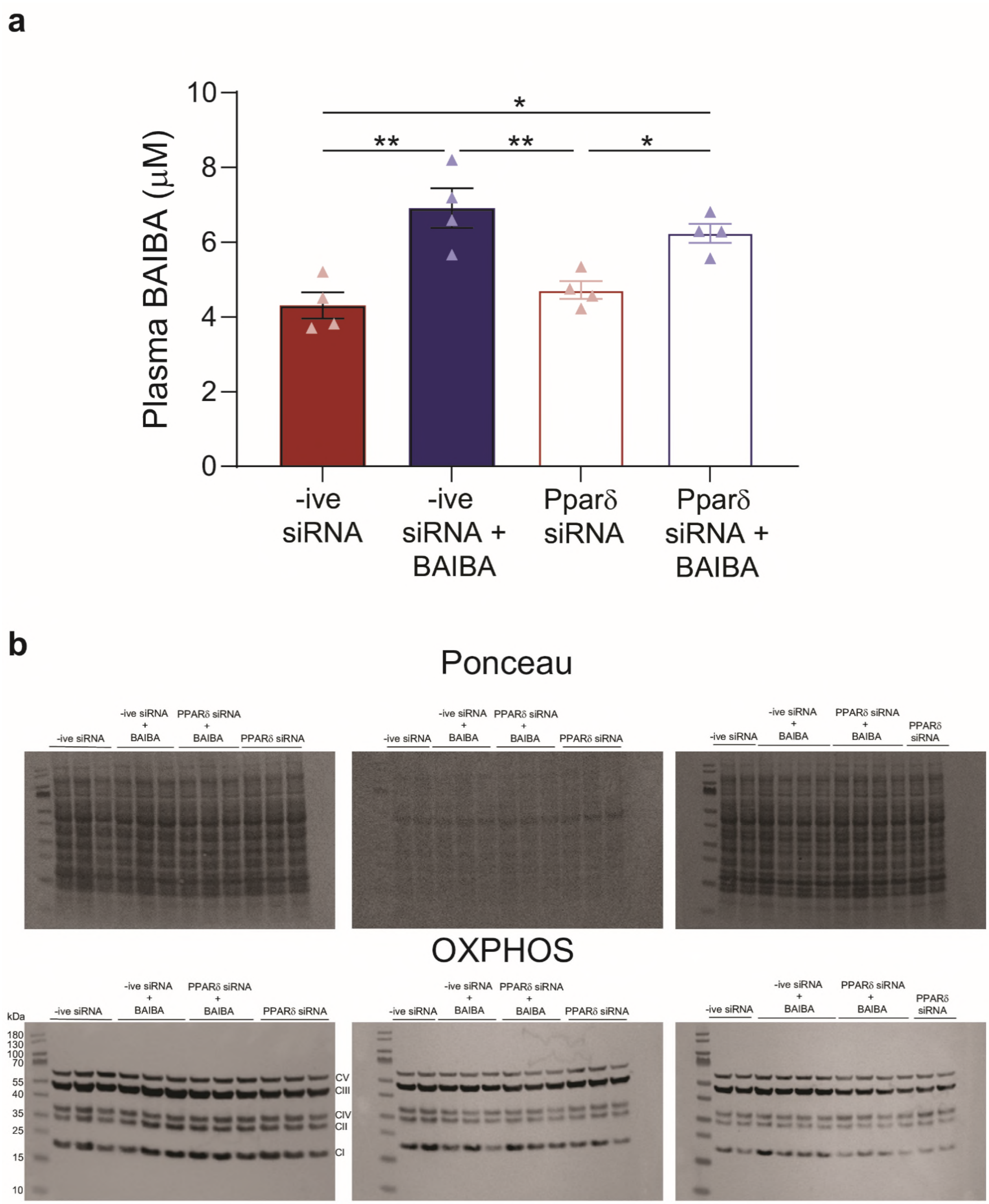

Supplementary Figure 6. (**a**) Plasma BAIBA concentration in mice with or without BAIBA treatment and receiving either scrambled negative control siRNA (-ive siRNA) or siRNA against Pparδ (*Pparδ* siRNA) directly into their hind limbs. (n = 4; ive siRNA vs –ive siRNA +

BAIBA $p = 0.0013$, -ive siRNA + BAIBA vs PPARδ siRNA $p = 0.005$, -ive siRNA vs PPARδ siRNA + BAIBA $p = 0.012$, PPARδ siRNA vs PPARδ siRNA + BAIBA $p = 0.049$; One-way ANOVA). -ive control siRNA solid red, -ive control siRNA + BAIBA solid blue, PPARδ siRNA red outline, PPARδ siRNA + BAIBA blue outline. (**b**) Immunoblots for soleus mitochondrial respiratory chain complexes I-V of mice with or without BAIBA treatment and receiving either -ive siRNA or Pparδ siRNA directly into their hind limbs (Biological, -ive siRNA n = 7, *Pparδ* siRNA n = 10, -ive siRNA + BAIBA n = 8, *Pparδ* siRNA + BAIBA n = 10) (n = 3 technical repeats). Ponceau stain provided for total protein control. Data in bar charts are mean ± SEM with data points shown. * $p \leq 0.05$, ** $p < 0.01$. Source data are provided as a Source Data file.

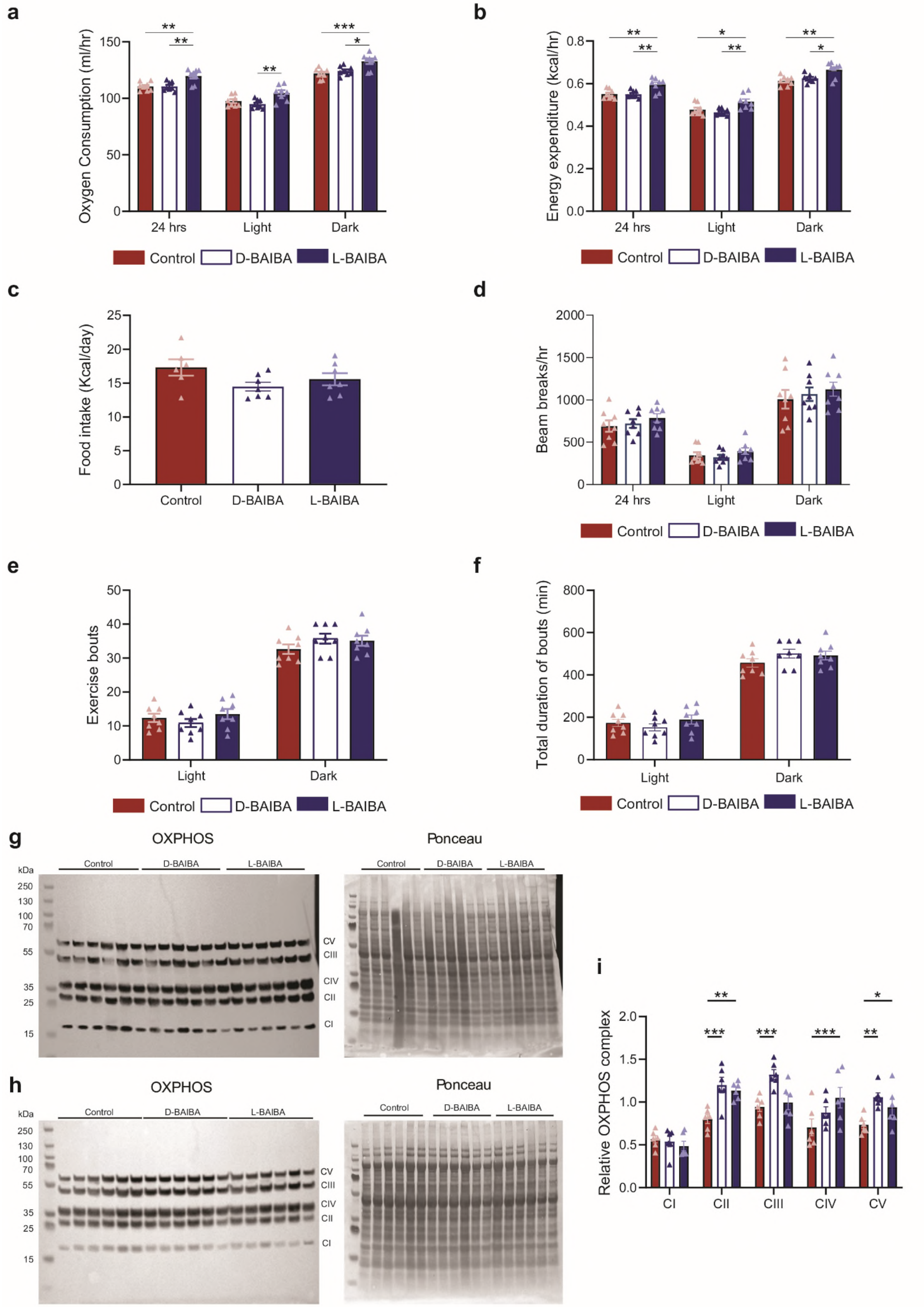
a
Oxygen Consumption (ml/hr)
24 hrs
Light
Dark
Control
D-BAIBA
L-BAIBA
b
Energy expenditure (kcal/hr)
c
Food intake (Kcal/day)
d
Beam breaks/hr
e
Exercise bouts
f
Total duration of bouts (min)
g
OXPHOS
Ponceau
kDa
CV
CIII
CIV
CII
CI
h
i
Relative OXPHOS complex

Supplementary Figure 7. L-BAIBA enhances voluntary exercise performance and muscle function in vivo. (**a**) Oxygen consumption in mice given either D-BAIBA or L-BAIBA compared to control mice (n = 8; 24 hrs, Control vs L-BAIBA $p = 0.005$, D-BAIBA vs L-BAIBA $p = 0.0047$; Light phase, D-BAIBA vs L-BAIBA $p = 0.0025$; Dark phase, Control vs L-BAIBA $p = 0.0006$, D-BAIBA vs L-BAIBA $p = 0.01$; Two-way ANOVA). (**b**) Energy expenditure in mice given either D-BAIBA or L-BAIBA compared to control mice (n = 8; 24 hrs, Control vs L-BAIBA $p = 0.009$, D-BAIBA vs L-BAIBA $p = 0.0085$; Light phase, Control vs L-BAIBA $p = 0.046$, D-BAIBA vs L-BAIBA $p = 0.0027$; Dark phase, Control vs L-BAIBA $p = 0.0017$, D-BAIBA vs L-BAIBA $p = 0.024$; Two-way ANOVA). (**c**) Food intake in mice given D-BAIBA or L-BAIBA compared to control mice (Control n = 6; D-BAIBA, L-BAIBA n = 7). (**d**) Locomotor activity in mice given D-BAIBA or L-BAIBA compared to control mice (n = 8). (**e**) Average number of exercise bouts (24 hr diurnal light and dark phases) in mice given D-BAIBA or L-BAIBA compared to control mice (n = 8). (**f**) Total duration of exercise bouts (24 hr diurnal light and dark phases) in mice given either D-BAIBA or L-BAIBA compared to control mice (n = 8). (**g**) Soleus immunoblots for respiratory chain complexes I-V of mice given D-BAIBA or L-BAIBA compared to control mice (n = 6 biological) (n = 3 technical repeats). Ponceau stain provided for total protein control. (**h**) Extensor digitorum longus immunoblots for respiratory chain complexes I-V from mice given D-BAIBA or L-BAIBA compared to control mice (n = 6 biological) (n = 3 technical repeats). Ponceau stain provided for total protein control. (i) Quantitation of EDL immunoblot for respiratory complex proteins I–V of mice given D-BAIBA or L-BAIBA compared to control mice (n = 6; CII Control vs D-BAIBA $p = 0.0002$, Control vs L-BAIBA $p = 0.0015$; CIII, Control vs D-BAIBA $p = 0.0004$; CIV, Control vs L-BAIBA $p = 0.001$; CV Control vs D-BAIBA $p = 0.002$, Control vs L-BAIBA $p = 0.046$; Two-way ANOVA). Data in bar charts are mean ± SEM with data points shown. Control = red, D-BAIBA = blue outline, L-BAIBA = blue fill. * $p \leq 0.05$, ** $p < 0.01$, *** $p < 0.001$. Source data are provided as a Source Data file.

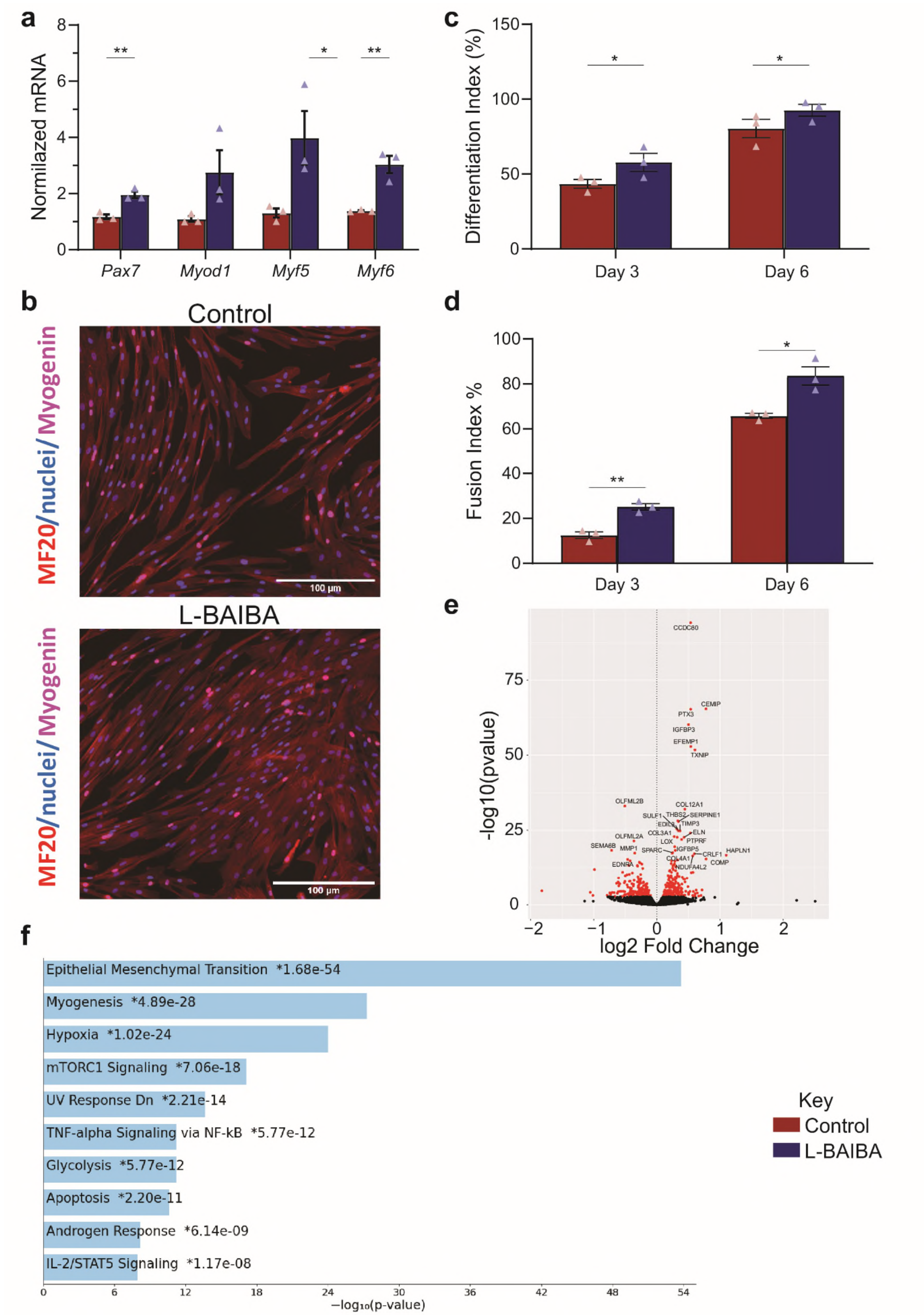

Supplementary Figure 8. The effects of L-BAIBA on human primary skeletal myocyte differentiation. (**a**) *Paired box protein* (*PAX-7*), *Myoblast determination protein 1* (*MYOD1*),

Myogenic factor 5 (*MYF5*), *MYF6* expression in human myotubes treated with L-BAIBA (10μM) for 72h post-seeding (n = 3; *PAX7* $p = 0.005$, *MYF5* $p = 0.05$, *MYF6* $p = 0.006$; two-tailed Student's t-test). (**b**) Representative confocal images of human myotubes treated with L-BAIBA (10 μM) and stained for Myosin heavy chain 2 (MF20; red), nuclei (blue) and myogenin (magenta) (Scale bar = 100μm). (**c**) The differentiation index in L-BAIBA (10μM) treated myoblasts differentiated to myotubes at day 3 and day 6 of differentiation (n = 3; day 3 $p = 0.04$, day 6 $p = 0.05$; paired Student's t-test). (**d**) The fusion index in L-BAIBA (10μM) treated myoblasts differentiated to myotubes at day 3 and day 6 of differentiation (n = 3; day 3 $p = 0.004$, day 6 $p = 0.042$; paired Student's t-test). (**e**) Volcano plot of differentially expressed genes identified from RNAseq data from control and L-BAIBA (10 μM) treated human myoblasts differentiated to myotubes over six days (n = 3; fold-change cut-off = 1.2; adjusted *p*-value cut-off = 0.05; Wald test with Benjamini-Hochberg). (**f**) Gene Set Enrichment Analysis of the RNAseq data from control and L-BAIBA (10 μM) treated human myoblasts differentiated to myotubes over six days (Fisher exact test adjusted for false discovery rate). Data in bar charts are mean ± SEM with data points shown. Control = red, L-BAIBA = blue. * $p \leq 0.05$, ** $p < 0.01$. Source data are provided as a Source Data file.

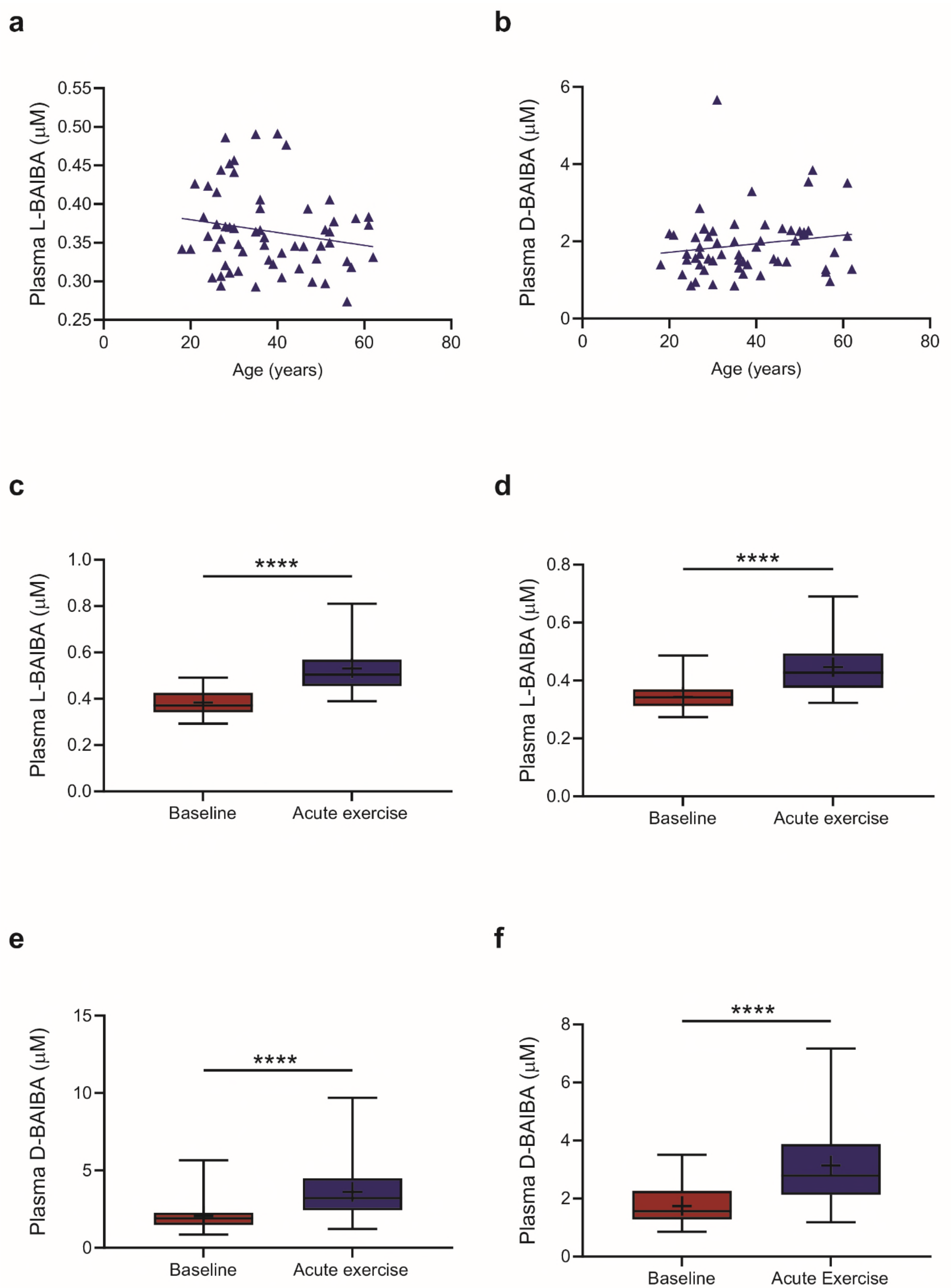

a
Plasma L-BAIBA (μM)
0.55
0.50
0.45
0.40
0.35
0.30
0.25
0
20
40
60
80
Age (years)
b
Plasma D-BAIBA (μM)
6
4
2
0
0
20
40
60
80
Age (years)
c
Plasma L-BAIBA (μM)
1.0
0.8
0.6
0.4
0.2
0.0
****
Baseline
Acute exercise
d
Plasma L-BAIBA (μM)
0.8
0.6
0.4
0.2
0.0
****
Baseline
Acute exercise
e
Plasma D-BAIBA (μM)
15
10
5
0
****
Baseline
Acute exercise
f
Plasma D-BAIBA (μM)
8
6
4
2
0
****
Baseline
Acute Exercise

Supplementary Figure 9. The relationship between plasma D-BAIBA and L-BAIBA and sex and age in humans. (**a**) Serum L-BAIBA did not significantly correlate with age in our study population (n = 60; $r = 0.18$, $p = 0.15$). (**b**) Serum D-BAIBA did not significantly correlate with age in our study population (n = 60; $r = 0.16$, $p = 0.22$). (**c**) Serum L-BAIBA concentration is increased in male participants following an acute exercise intervention. (n = 32, $p < 0.0001$, paired two-tailed t-test). (**d**) Serum L-BAIBA concentration is increased in female participants following an acute exercise intervention. (n = 28, $p < 0.0001$, paired two-tailed t-test). (**e**) Serum D-BAIBA concentration is increased in male participants following an acute exercise intervention. (n = 32, $p < 0.0001$, paired two-tailed t-test). (**f**) Serum D-BAIBA concentration is increased in female participants following an acute exercise intervention. (n = 28, $p < 0.0001$, paired two-tailed t-test). Box and whisker plots show 25th to 75th percentile (box) min to max (whiskers), mean (+) and median (−). Baseline = red; Acute exercise = blue. **** $p < 0.0001$. Source data are provided as a Source Data file.

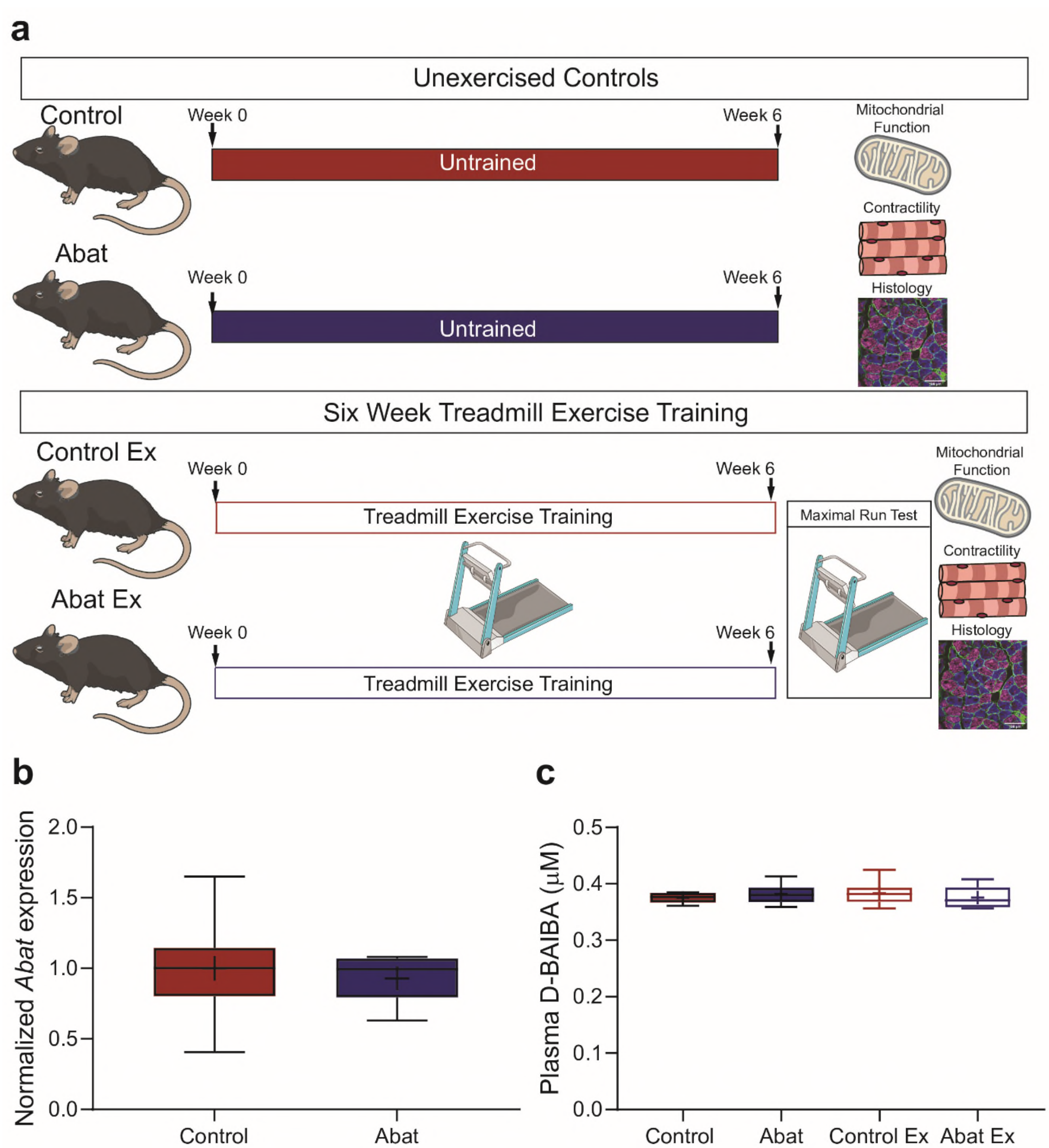

Supplementary Figure 10. (**a**) Schematic showing C57BL6/J mice receiving either scrambled short DNA antisense oligonucleotide (Gapmers) (Control) or short DNA antisense oligonucleotide against 4-Aminobutyrate Aminotransferase (*Abat*), intramuscularly into the gastrocnemius muscles of both hind limbs twice a week to knockdown Abat expression. Abat knockdown and control groups were subdivided into an unexercised group and a group that underwent a 6-week treadmill exercise training programme 4 days a week at 25 cm/s and 10% grade during a 45-minute session per day. This schematic contains images provided by Servier Medical Art (https://smart.servier.com), licensed under CC BY 4.0 (https://creativecommons.org/licenses/by/4.0/). (**b**) Liver *Abat* expression in mice receiving short DNA antisense oligonucleotide against Abat, intramuscularly into gastrocnemius twice a

week. Control n = 10, *Abat* n = 9. (**c**) Plasma D-BAIBA concentration in mice receiving either scrambled negative control GapmeR (Control; red) or GapmeR against Abat (Abat; blue) injections into the soleus twice a week for six weeks with (outline) or without (solid fill) treadmill exercise (Ex) training (n = 10). Box and whisker plots show 25th to 75th percentile (box) min to max (whiskers), mean (+) and median (−). Source data are provided as a Source Data file.

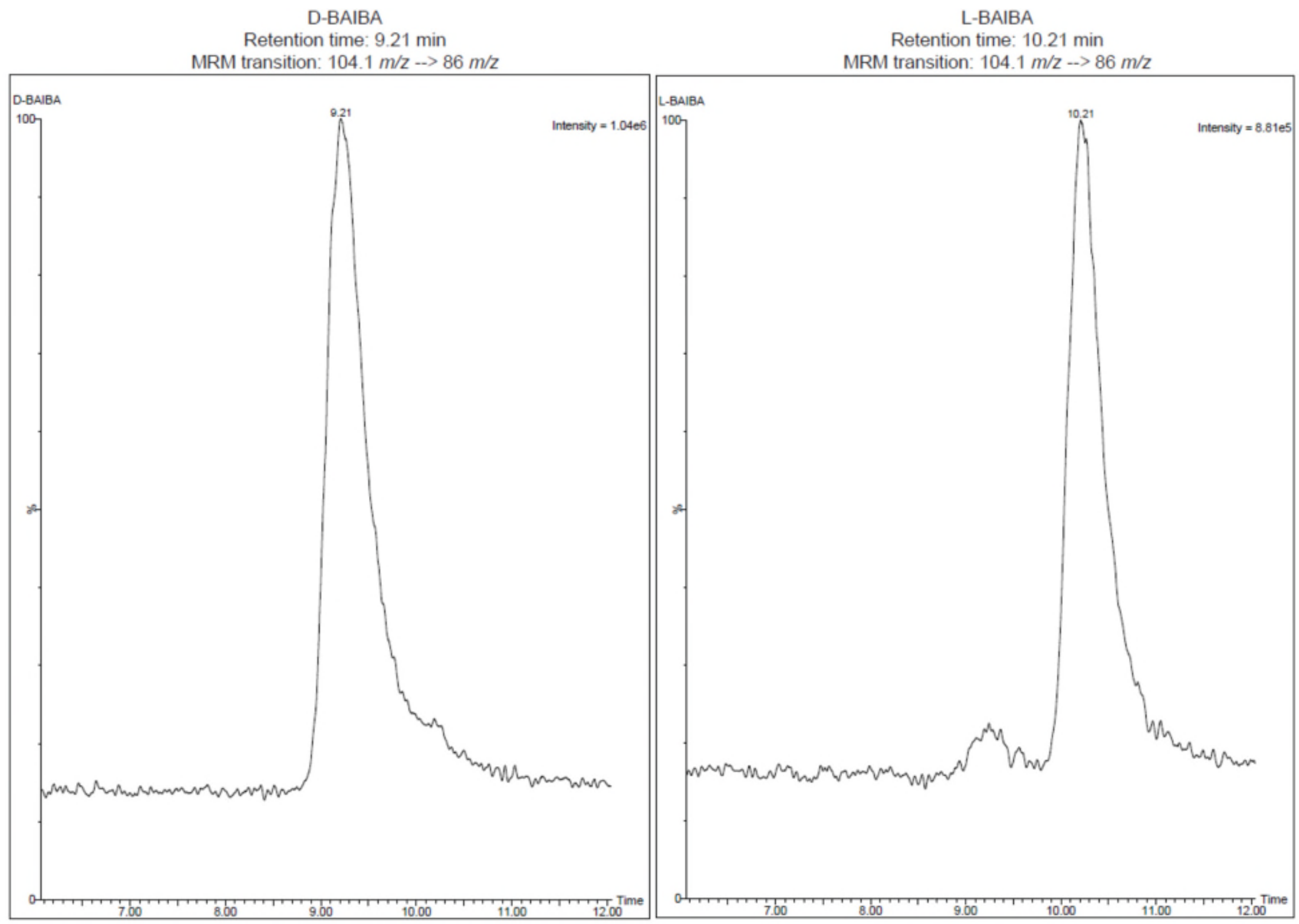

Supplementary Figure 11. Chromatograms showing separation of the D-BAIBA and L-BAIBA enantiomers by Liquid Chromatography-Mass Spectrometry. A Multiple Reaction Monitoring (MRM) transition of 104.1 *m/z* → 86 *m/z* was used for both enantiomers. D-BAIBA retention time = 9.21 min, L-BAIBA retention time = 10.21 min.
