# Supplementary material for: The metabokine β-aminoisobutyric acid mediates exercise performance and skeletal muscle adaptation through a PGC1α-BAIBA-PPARδ axis
Source: Nat Commun. 2026 Aug 18;17:8090. doi: 10.1038/s41467-026-76307-8 (PMC13487176; doi:10.1038/s41467-026-76307-8)
Supplement: Supplementary file 2 [file 41467_2026_76307_MOESM2_ESM.pdf]

nature portfolio

Corresponding author(s): Lee D Roberts

Last updated by author(s): 26/06/26

Reporting Summary

Nature Portfolio wishes to improve the reproducibility of the work that we publish. This form provides structure for consistency and transparency in reporting. For further information on Nature Portfolio policies, see our Editorial Policies and the Editorial Policy Checklist.

Statistics

For all statistical analyses, confirm that the following items are present in the figure legend, table legend, main text, or Methods section.

n/a	Confirmed	
☐	☒	The exact sample size (*n*) for each experimental group/condition, given as a discrete number and unit of measurement
☐	☒	A statement on whether measurements were taken from distinct samples or whether the same sample was measured repeatedly
☐	☒	The statistical test(s) used AND whether they are one- or two-sided<br>*Only common tests should be described solely by name; describe more complex techniques in the Methods section.*
☐	☒	A description of all covariates tested
☐	☒	A description of any assumptions or corrections, such as tests of normality and adjustment for multiple comparisons
☐	☒	A full description of the statistical parameters including central tendency (e.g. means) or other basic estimates (e.g. regression coefficient) AND variation (e.g. standard deviation) or associated estimates of uncertainty (e.g. confidence intervals)
☐	☒	For null hypothesis testing, the test statistic (e.g. *F*, *t*, *r*) with confidence intervals, effect sizes, degrees of freedom and *P* value noted<br>*Give P values as exact values whenever suitable.*
☒	☐	For Bayesian analysis, information on the choice of priors and Markov chain Monte Carlo settings
☒	☐	For hierarchical and complex designs, identification of the appropriate level for tests and full reporting of outcomes
☐	☒	Estimates of effect sizes (e.g. Cohen's *d*, Pearson's *r*), indicating how they were calculated

Our web collection on statistics for biologists contains articles on many of the points above.

Software and code

Policy information about availability of computer code

Data collection

Confocal images were collected and analysed using Columbus software version 2.9.1 (PerkinElmer). Indirect calorimetry and wheel running data was collected using Oxymax (version 5.37.05, Columbus Instruments). Respirometry data was collected using Oxygraph 2k DatLab software (version 6.1, OROBOROS Instruments). Histology images were collected using Zen 2 Pro (version 2.0, ZEISS). A Fluorescence microscope slide scanner (Axioscan Z1, ZEISS) was used to collect flourescent images. Images were recorded using ZEN software (Version 3.40, ZEISS). RT-qPCR data was collected using StepOne Software (version 2.1 Applied Biosystems). Westernblot images were recorded using iBrightTM FL1500 imaging system (Invitrogen). Mass spectrometry data were recorded using Waters MassLynx Version 4.1 (Waters, USA). Seahorse XFe96 Bioanalyzer Data was collected using Wave Controller (version 2.6.1 Agilent). MRI data was collected using Paravision software (Bruker Germany version 6.0.1). Muscle contractile data was collected using Dynamic Muscle Control software (version 5; Aurora Scientific, Aurora, Canada). Valiant 2 CPET was operated with Lode Ergometry Mananager software (version 9; Lode Groningen, the Netherlands). RNAseq data was collected using TapeStation Analysis Software (version 3; Agilent).

Data analysis

Indirect calorimetry and wheel running data was analyzed and p-values were calculated using ANCOVA / Generalized Linear Model with body mass as a covariate using CalR (version 1.3) (https://calrapp.org/). Univariate data analysis was performed using Prism Graphpad (version 10). Confocal images were analysed using Columbus software version 2.9.1 (PerkinElmer). Data analysis of RNA-Seq data was performed using R package edgeR v3.8.6. Mitochondrial respiration profiles were assessed using Seahorse Wave Software (version 2.6.1) (Agilent Technologies, Waldbronn, Germany). Flourescent images were recorded using ZEN software (Version 3.40, ZEISS). RT-qPCR data was analyzed using StepOne Software (version 2.1 Applied Biosystems). MRI data were analysed in MATLAB (2022; MathWorks, Natick, USA). The proton density fat fraction was analysed using Osirix Lite v11.0.2. Centralized nuclei in muscle injury studies were quantified using ImageJ 1.54f software (National Institutes of Health, Bethesda, MD, USA). Westernblot quantitative densitometry was performed using ImageJ 1.54f software National Institutes of Health, Bethesda, MD, USA). Mass spectrometery data were processed and peak integration performed using Waters

TargetLynx Version 4.1 (Waters, USA). RNAseq differentially expressed genes were identified using DESeq2 package (v1.40). Gene ontology enrichment was performed using ClusterProfileR (v4.14.4). Gene Set Enrichment Analysis was performed using Enrichr (2021).

For manuscripts utilizing custom algorithms or software that are central to the research but not yet described in published literature, software must be made available to editors and reviewers. We strongly encourage code deposition in a community repository (e.g. GitHub). See the Nature Portfolio guidelines for submitting code & software for further information.

Data

Policy information about availability of data

All manuscripts must include a data availability statement. This statement should provide the following information, where applicable:

- Accession codes, unique identifiers, or web links for publicly available datasets
- A description of any restrictions on data availability
- For clinical datasets or third party data, please ensure that the statement adheres to our policy

RNA-Seq data associated with this study are available from EBI ArrayExpress repository (https://www.ebi.ac.uk/biostudies/arrayexpress) can be accessed via https://www.ebi.ac.uk/arrayexpress/experiments/E-MTAB-14746 (human skeletal muscle cell datasets) and https://www.ebi.ac.uk/arrayexpress/experiments/E-MTAB-14747 (murine soleus datasets). Source Data are provided with this paper.

Research involving human participants, their data, or biological material

Policy information about studies with human participants or human data. See also policy information about sex, gender (identity/presentation), and sexual orientation and race, ethnicity and racism.

Reporting on sex and gender

For our acute exercise intervention study of humans both male and female participants were recruited. Of the total study population (60 individuals) 28 were female and 32 were male. Data is disaggregated by sex both in our manuscript and in our source data. Sex was determined by self-reporting and confirmed from participants' clinical notes. The effects of exercise on circulating levels of L-BAIBA and D-BAIBA did not differ by sex and did not show sexual dimorphism.

Reporting on race, ethnicity, or other socially relevant groupings

Socially constructed variables were not used in this study.

Population characteristics

For human analyses these are outlined within our manuscript in Supplementary tables 1 and 2.

For our human acute exercise intervention study, participants were aged 18-65 years, non-smokers, and free from chronic disease (except type 1 diabetes). Participants were excluded if they had diabetes-related complications, other chronic conditions, history of smoking, or BP >140/90 mmHg at study visits. Sixty participants were enrolled in total and both male and female participants were included. Participant demographics are shown in Supplementary Table1. Participants provided written informed consent prior to enrolment following approval from the NHS HRA North East Tyne & Wear South Research Ethics and Newcastle University Ethics Committees (16/NE/0192, ISRCTN63739203).

Patient demographics in supplementary table 1 include number of participants (60), number of male participants (32), number of female participants (28), and age (37.9 +/- 1.2 years).

For our aerobic exercise training study healthy volunteers gave full written informed consent to participate in a 10-wk endurance training program. Primary inclusion criteria were nonsmoker, free of injury, and not using any medication or nutritional supplements. Additional exclusion criteria that would preclude successful participation in the training study included (diagnosed) lactose intolerance and/or dairy protein allergy, cardiorespiratory-related illness, and musculoskeletal-related injuries that would impede endurance training sessions. All subjects were physically active, performing sports on a noncompetitive basis between 1 and 4 h/wk. None of the participants had a history of participating in any structured endurance training programs to improve performance within the past 2 y. Our study used 33 subjects for which blood plasma was available from a total of 44 recruited. Participant demographics are shown in Supplementary Table 2. The study was carried out in accordance with the guidelines for human research of the Medical Ethical Committee of Wageningen University. The Medical Ethical Committee of Wageningen University approved all study procedures and complied with the guidelines set by the Declaration of Helsinki of 1975 as revised in 1983.

Patient demographics described in Supplementary Table 2 include number of participants (33) and age (22.35 +/- 1.53 years).

Recruitment

Studies were advertised widely through poster, email and social media recruitment campaigns. Participants were recruited either at the National Institute for Health Research Newcastle Clinical Research Facility (acute exercise study) or Wageningen University (endurance exercise training study). Participants were only excluded if they failed to meet the inclusion criteria or explicitly met the exclusion criteria detailed in the manuscript. Our study has the potential to be impacted by volunteer participation bias since health conscious individuals are more likely to join exercise studies. Our Acute exercise study recruited from both University (University of Newcastle) and clinical (NIHR clinical research facility) reducing the impact of sampling bias and reflected in an older population when compared to the endurance exercise study which recruited from a University environment and had younger participants and therefore maybe susceptible to sampling bias from self-selected environments. The congruity of effects between the two cohorts (for example correlation of plasma L-BAIBA with VO2 max suggest sampling bias is unlikely to have affected our results.

Ethics oversight

Our human acute exercise intervention study received NHS HRA North East Tyne & Wear South Research Ethics and Newcastle University Ethics Committees (16/NE/0192, ISRCTN63739203) approval as outlined in our methods section of the manuscript. For our human endurance exercise training programme study, this received ethical approval from the Medical

Ethical Committee of Wageningen University, in accordance with the Declaration of Helsinki. These details are outlined within our methods section.

Note that full information on the approval of the study protocol must also be provided in the manuscript.

Field-specific reporting

Please select the one below that is the best fit for your research. If you are not sure, read the appropriate sections before making your selection.

☒ Life sciences ☐ Behavioural & social sciences ☐ Ecological, evolutionary & environmental sciences

For a reference copy of the document with all sections, see nature.com/documents/nr-reporting-summary-flat.pdf

Life sciences study design

All studies must disclose on these points even when the disclosure is negative.

Sample size	Sample sizes for experiments were calculated using power calculations. Standard deviations were based on previous studies or on the available literature. Power calculations were made using beta = 0.8 and alpha = 0.05. Power calculations were made using the following formula: k = n2 / n1=1, n1=(σ12+σ22/K)(z1−α/2+z1−β)2/Δ2 n2=K?n1 where, Δ = |μ2-μ1| = absolute difference between two means, σ1, σ2 = variance of mean 1 and 2, n1 = sample size for group 1, n2 = sample size for group 2, α = probability of type I error (0.05), β = probability of type II error (0.2), z = critical Z value for a given α or β, k = ratio of sample size for group 2 to group 1. Power calculations were made according to Rosner B. Fundamentals of Biostatistics. 7th ed. Boston, MA: Brooks/Cole; 2011.
Data exclusions	Data was only excluded where whole experiments failed (based on positive or negative controls, animals or cells failed to meet experiment endpoint or variance of internal standards). These criteria were pre-defined.
Replication	Replicate experiments were successful. All experiments were replicated a minimum of three times with the exception of the human volunteer and animal studies (which were sufficiently powered), which were each performed independently once due to ethical limitations on repeated human and animal studies.
Randomization	All samples were randomly assigned to experimental groups. Animals were weight matched and randomly assigned to experimental groups. Cells in culture wells were randomly assigned to study groups.
Blinding	For animal studies researchers were blinded to experimental grouping by core technicians in the animal facility to avoid bias and aid reproducibility. Experimentalists were blinded to study groups for cell culture endpoint data collection and analysis. Experimentalists were blinded to human patient data and group allocation for human biofluid analysis by clinical and research staff.

Reporting for specific materials, systems and methods

We require information from authors about some types of materials, experimental systems and methods used in many studies. Here, indicate whether each material, system or method listed is relevant to your study. If you are not sure if a list item applies to your research, read the appropriate section before selecting a response.

Materials & experimental systems

n/a	Involved in the study	
☐	☒	Antibodies
☐	☒	Eukaryotic cell lines
☒	☐	Palaeontology and archaeology
☐	☒	Animals and other organisms
☒	☐	Clinical data
☒	☐	Dual use research of concern
☒	☐	Plants

Methods

n/a	Involved in the study	
☒	☐	ChIP-seq
☒	☐	Flow cytometry
☒	☐	MRI-based neuroimaging

Antibodies

Antibodies used	Total OXPHOS Rodent WB Antibody Cocktail https://www.abcam.com/en-us/products/panels/total-oxphos-rodent-wb-antibody-cocktail-ab110413 Abcam, Cat. no. ab110413. Five mouse mAbs, one each against CI subunit NDUFB8 (ab110242; Clone 220E9DH10C12), CII-30kDa (ab14714; Clone 21A11AE7), CIII-Core protein 2 (ab14745; clone 13G12AF12BB11), CIV subunit I (ab14705, clone 1D6E1A8) and CV alpha subunit (ab14748, clone 15H4C4) as an optimized premixed cocktail (1:1000 dilution).<br>m-IgG Fc BP-HRP (recombinant protein; sc-525409, Santa Cruz; 1:1000 dilution) (https://www.scbt.com/p/m-igg-fc-bp-hrp?srsltid=AfmBOoo1KJJ1PcxZMc3MJgT4UCnWZw__ZmIkuM-2vnCN0btYs63haBPg)<br>monoclonal anti-myogenin antibody, clone F5D, Invitrogen 14-5643-82; 1:150 dilution https://www.thermofisher.com/antibody/product/Myogenin-Antibody-clone-F5D-Monoclonal/14-5643-82<br>Monoclonal MF20 Myosin heavy chain, sarcomere (MHC) MF20 (clone MF-20, Developmental Studies Hybridoma Bank catalogue no. MF20 2147781, 1;100 dilution) https://dshb.biology.uiowa.edu/MF-20<br>Polyclonal Goat anti-Mouse IgG1 Alexa Fluor 555 Thermofisher catalogue number A-21127 dilution 1:150 https://

www.thermofisher.com/antibody/product/Goat-anti-Mouse-IgG1-Cross-Adsorbed-Secondary-Antibody-Polyclonal/A-21127
Monoclonal antibodies BA-D5 (anti-MHC-I/ β-MHC/Myh7 specific, IgG2b, clone BA-D5, Developmental Studies Hybridoma Bank catalogue no. BA-D5 1:100 dilution). https://dshb.biology.uiowa.edu/BA-D5
Monoclonal anti-MHC-IIa SC-71 (IgG1, clone number SC-71, Developmental Studies Hybridoma Bank catalogue no. SC-71, 1:100 dilution). https://dshb.biology.uiowa.edu/SC-71
monoclonal anti-MHC-IIb specific, IgM, clone BF-F3, Developmental Studies Hybridoma Bank catalogue no. BF-F3, 1:100 dilution https://dshb.biology.uiowa.edu/BF-F3
monoclonal anti-MHC-IIx specific, IgM, clone 6H1, Developmental Studies Hybridoma Bank catalogue no. 6H1, 1:100 dilution https://dshb.biology.uiowa.edu/6H1
Polyclonal Laminin Anti-Laminin antibody produced in rabbit Sigma-Aldrich catalogue no. L9393, 1:50 dilution https://www.sigmaaldrich.com/catalog/product/sigma/l9393?lang=en&region=GB
Fab Fragment Goat Anti-Mouse IgGFAB, Jackson ImmunoResearch Catalogue no. 115-007-003, 1:100 dilution https://www.jacksonimmuno.com/catalog/products/115-007-003
Polyclonal goat anti-Mouse IgG2b Cross-Adsorbed Secondary Antibody, Alexa Fluor® 647 (Invitrogen catalogue no. A-21242) dilution 1:400. https://www.thermofisher.com/antibody/product/Goat-anti-Mouse-IgG2b-Cross-Adsorbed-Secondary-Antibody-Polyclonal/A-21242
Polyclonal goat anti-mouse IgG1 cross-absorbed secondary antibody Alexa Fluor® 350 (Invitrogen catalogue no. A21120) dilution 1:400. https://www.thermofisher.com/antibody/product/Goat-anti-Mouse-IgG1-Cross-Adsorbed-Secondary-Antibody-Polyclonal/A21120
Polyclonal goat anti-mouse IgG1 cross-adsorbed secondary antibody, Alexa Fluor® 488 (Invitrogen catalogue no. A-21121) dilution 1:400. https://www.thermofisher.com/antibody/product/Goat-anti-Mouse-IgG1-Cross-Adsorbed-Secondary-Antibody-Polyclonal/A-21121
Polyclonal goat anti-mouse IgM conjugated with Alexa Fluor® 555 (Invitrogen catlogue no. A-21426), 1:400 dilution https://www.thermofisher.com/antibody/product/Goat-anti-Mouse-IgM-Heavy-chain-Cross-Adsorbed-Secondary-Antibody-Polyclonal/A-21426
polyclonal goat anti-Rabbit IgG (H+L) Highly cross-adsorbed secondary antibody Alexa Fluor® 488 (Invitrogen catalogue no. A-11034) dilution 1:400. https://www.thermofisher.com/antibody/product/Goat-anti-Rabbit-IgG-H-L-Highly-Cross-Adsorbed-Secondary-Antibody-Polyclonal/A-11034

Validation

Fab Fragment Goat Anti-Mouse IgGFAB, Jackson ImmunoResearch Catalogue no. 115-007-003. Validation: Based on antigen-binding assay and/or ELISA, the antibody reacts with whole molecule mouse IgG. It also reacts with the light chains of other mouse immunoglobulins. No antibody was detected against non-immunoglobulin serum proteins. Details on suppliers website: www.jacksonimmuno.com/catalog/products/115-007-003
Total OXPHOS Rodent WB Antibody Cocktail ab110413; Abcam. Validated for Westernblot. Details on suppliers website: https://www.abcam.com/en-us/products/panels/total-oxphos-rodent-wb-antibody-cocktail-ab110413
m-IgG Fc BP-HRP (recombinant protein; sc-525409, Santa Cruz). Validated as a secondary antibody for mouse IgG in westernblot. Details on suppliers website: https://www.scbt.com/p/m-igg-fc-bp-hrp?srsltid=AfmBOoo1KJJ1PcxZMc3MJgT4UCnWZw__ZmIkuM-2vnCN0btYs63haBPg
monoclonal anti-myogenin antibody, clone F5D, Invitrogen 14-5643-82. Validated for GS, IF, IHC (P), IP, and WB applications. Details on suppliers website: https://www.thermofisher.com/antibody/product/Myogenin-Antibody-clone-F5D-Monoclonal/14-5643-82
Monoclonal MF20 Myosin heavy chain, sarcomere (MHC) Antibody Registry ID: AB_2147781. Developmental Studies Hybridoma Bank catalogue no. MF20 Validated for ELISA, FACS, FFPE, Immunofluorescence, Immunohistochemistry, Immunoprecipitation, Western Blot. Details on suppliers website: https://dshb.biology.uiowa.edu/MF-20
Polyclonal goat anti-Mouse IgG1 Alexa Fluor 555 Thermofisher catalogue number A-21127. Validated as a secondary for immunofluorescence. Details on suppliers website: https://www.thermofisher.com/antibody/product/Goat-anti-Mouse-IgG1-Cross-Adsorbed-Secondary-Antibody-Polyclonal/A-21127
Monoclonal antibodies BA-D5 (anti-MHC-I/ β-MHC/Myh7 specific, IgG2b, clone BA-D5, Developmental Studies Hybridoma Bank catalogue no. BA-D5. Antibody Registry ID: AB_2235587. Validated for Immunofluorescence, Immunohistochemistry, Western Blot. Details on suppliers website: https://dshb.biology.uiowa.edu/BA-D5
monoclonal anti-MHC-IIb specific, IgM, clone BF-F3, Developmental Studies Hybridoma Bank catalogue no. BF-F3, Antibody Registry ID: AB_2266724 Validation: for ELISA, Immunofluorescence, Immunohistochemistry, Western Blot. Details on suppliers website: https://dshb.biology.uiowa.edu/BF-F3
Monoclonal anti-MHC-IIa SC-71 (IgG1, clone number SC-71, Developmental Studies Hybridoma Bank catalogue no. SC-71. alidated for Immunofluorescence, Immunohistochemistry, Western Blot. Details on suppliers website: https://dshb.biology.uiowa.edu/SC-71
monoclonal anti-MHC-IIx specific, IgM, clone 6H1, Developmental Studies Hybridoma Bank catalogue no. 6H1. Antibody Registry ID: AB_2314830. Validation for FFPE, Immunofluorescence, Immunohistochemistry, Western Blot. Details on suppliers website: https://dshb.biology.uiowa.edu/6H1
Polyclonal Laminin Anti-Laminin antibody produced in rabbit Sigma-Aldrich catalogue no. L9393. Validated for ARR, DB, IHC (p). Details on suppliers website: https://www.sigmaaldrich.com/catalog/product/sigma/l9393?lang=en&region=GB
Polyclonal goat anti-Mouse IgG2b Cross-Adsorbed Secondary Antibody, Alexa Fluor® 647 (Invitrogen catalogue no. A-21242). Validated as a secondary antibody for immunofluorescence. Details on suppliers website: https://www.thermofisher.com/antibody/product/Goat-anti-Mouse-IgG2b-Cross-Adsorbed-Secondary-Antibody-Polyclonal/A-21242
Polyclonal goat anti-mouse IgG1 cross-absorbed secondary antibody Alexa Fluor® 350 (Invitrogen catalogue no. A21120). Validated as a secondary antibody for immunofluorescence. Details on suppliers website: https://www.thermofisher.com/antibody/product/Goat-anti-Mouse-IgG1-Cross-Adsorbed-Secondary-Antibody-Polyclonal/A21120
Polyclonal goat anti-mouse IgG1 cross-adsorbed secondary antibody, Alexa Fluor® 488 (Invitrogen catalogue no. A-21121) Validated as a secondary antibody for immunofluorescence. Details on suppliers website: https://www.thermofisher.com/antibody/product/Goat-anti-Mouse-IgG1-Cross-Adsorbed-Secondary-Antibody-Polyclonal/A-21121
Polyclonal goat anti-mouse IgM conjugated with Alexa Fluor® 555 (Invitrogen catlogue no. A-21426). Validated as a secondary antibody for immunofluorescence. Details on suppliers website: https://www.thermofisher.com/antibody/product/Goat-anti-Mouse-IgM-Heavy-chain-Cross-Adsorbed-Secondary-Antibody-Polyclonal/A-21426
polyclonal goat anti-Rabbit IgG (H+L) Highly cross-adsorbed secondary antibody Alexa Fluor® 488 (Invitrogen catalogue no. A-11034) alidated as a secondary antibody for immunofluorescence. Details on suppliers website: https://www.thermofisher.com/antibody/product/Goat-anti-Rabbit-IgG-H-L-Highly-Cross-Adsorbed-Secondary-Antibody-Polyclonal/A-11034

Eukaryotic cell lines

Policy information about cell lines and Sex and Gender in Research

Cell line source(s)	Adult primary human skeletal myoblasts were purchased from Cell Applications Inc (Cat no. 150-05a).
Authentication	Human primary skeletal myoblasts (Cell Application Inc Cat no. 150-05a) are authenticated by the supplier. In addition myoblasts are tested for their ability to attach, spread, proliferate and differentiate in culture conditions. The cells are authenticated for their ability to form multinucleated myotubes using morphological and gene/protein expression markers following differentiation.
Mycoplasma contamination	All cells have been tested for mycoplasma contamination by the providers (negative results).
Commonly misidentified lines (See ICLAC register)	No commonly misidentified cells lines were used in this study.

Animals and other research organisms

Policy information about studies involving animals; ARRIVE guidelines recommended for reporting animal research, and Sex and Gender in Research

Laboratory animals	For BAIBA-treatment studies. Six-week-old male and female C57BL6/J mice (Charles River, UK) were weight-matched and assigned to groups for treatment.<br>For obesity studies six-week-old C57BL6/J mice (Charles River, UK) were weight-matched and assigned to groups for treatment.<br>For Abat knockdown studies: 6 week old C57BL6/J mice (Charles River, UK) were weight-matched and assigned to groups for treatment.<br>For Pparδ knockdown studies: 6 week old C57BL6/J mice (Charles River, UK) were weight-matched and assigned to groups for treatment.<br>For D- and L-BAIBA enantiomer studies: Six-week-old C57BL6/J mice (Charles River, UK) were weight-matched and assigned to groups for treatment.
Wild animals	The study did not involve wild animals.
Reporting on sex	Sex was considered in the study design. Data reported in figure 1 examined the effect of 6 weeks BAIBA treatment in male mice. Data reported in supplementary figure 2 examined the effects of 6 week BAIBA treatment in female mice. The data are therefore disaggregated for sex in the manuscript and the source data. No sexually dimorphic effects of BAIBA were identified and effects in male and female mice phenocopied.
Field-collected samples	The study did not involve animals collected from the field.
Ethics oversight	All studies carried out in the UK were regulated under the Animals (Scientific Procedures) Act 1986 and complied with national and local ethical regulations for animal research. All procedures were carried out in accordance with U.K. Home Office protocols under a U.K. Home Office Project License (PP8169223) by a U.K. Home Office Personal License Holder. Experiments carried out at the University of Sau Paulo were performed under institutional ethical approval (CEUA ICB USP #2144240425).

Note that full information on the approval of the study protocol must also be provided in the manuscript.

Plants

Seed stocks	N/A
Novel plant genotypes	N/A
Authentication	N/A
