# Supplementary material for: The metabokine β-aminoisobutyric acid mediates exercise performance and skeletal muscle adaptation through a PGC1α-BAIBA-PPARδ axis
Source: Nat Commun. 2026 Aug 18;17:8090. doi: 10.1038/s41467-026-76307-8 (PMC13487176; doi:10.1038/s41467-026-76307-8)
Supplement: Supplementary file 3 [file 41467_2026_76307_MOESM3_ESM.pdf]

nature portfolio

Peer Review File

The metabokine β-aminoisobutyric acid mediates exercise performance and skeletal muscle adaptation through a PGC1α-BAIBA-PPARδ axis

Corresponding Author: Professor Lee Roberts

This file contains all reviewer reports in order by version, followed by all author rebuttals in order by version.

Version 0:

Reviewer comments:

Reviewer #1

(Remarks to the Author)
This manuscript shows that BAIBA can enhance the positive effects of exercise and improved metabolic and functional phenotype in a mouse model of obesity. e. The authors have used three mouse models (WT animals plus/minus VWR and BAIBA, a model of muscle injection of an antisense to 4-aminobutyrate aminotransferase, ABAT, and a high fat diet model), ex vivo human myocytes, and a human study (correlation of plasma BAIBA to VO2 peak) to determine various functions of BAIBA, a muscle metabolite.
It is not clear why the authors only used 6 week old males for the voluntary wheel running experiments. These are immature animals perhaps equivalent to teenagers. Mature mice at least 4-5 months when the musculoskeletal system is considered mature should be used. Otherwise the extrapolation to humans is not appropriate. Females also should be used for comparison.
If there is an increase in the number of fibers, this suggests the muscles are immature and still developing. Muscle fibers in the mature muscle are post-mitotic.
It is stated that the muscles in the mice were injected 2 times per week. This would definitely induce muscle damage. It is not clear which muscles are being injected. What is the delivery vehicle for the antisense-virus or electroporation. Were major muscles in both hindlimbs injected in order to observe a decrease in circulating BAIBA? These important details are missing.
Another concern is the increase in activity in the animals injected with BAIBA as shown in Figure 1b and 2b. This would suggest that BAIBA is changing behavior that it is increasing wheel running. This change in behavior would need to be taken into account when interpreting the data.
The human studies are very preliminary. There is no breakdown with regards to age or sex. What is the correlation is simply due to an increased muscle mass? Additional studies should be performed.
The authors have failed to acknowledge that there are two enantiomers of BAIBA with different functions and different tissue sources. This is important because the two enantiomers though signaling through the same receptor can have different effects through distinct signaling pathways. L but not D BAIBA has been shown to be released by contracted murine muscle. As ABAT play a major role in the production of L-BAIBA, the mouse with the reduction of ABAT in muscle is showing an inhibition of L, not D-BAIBA secretion. The commercial source of BAIBA used for all experiments is a mixture of both enantiomers and the LC-MS used for the quantitation of BAIBA only recognizes total BAIBA and not the two enantiomers. The RNAseq data would most likely be different if only L or only D-BAIBA were given to the animals.
The references provided for the safe dosing of BAIBA only used L, not D-BAIBA. There are no references to date for the dosing of D-BAIBA.
In this study, a correlation of total BAIBA circulating levels correlated with aerobic exercise capacity. Previous published studies have provided a correlation of either L or D BAIBA with physical performance PMID: 37821627. In this study the significant correlation was with D-BAIBA and physical performance. A discussion of these results could be included.
It would be important to go into more depth in signaling mechanisms. One easy experiment to perform is to determine if muscle expresses the Mas related G Protein Receptor type D, MRGPRD. There are several publications that have identified signaling pathways for both L and D BAIBA. It would be important to determine what these are in muscle.

Minor concerns:
What is the specificity of the PPARdelta inhibitor used GW3787 also known as GSK3787? It appears to also be a PPARbeta

inhibitor see PMID: 20516370. The outcomes with this inhibitor is replicated with siPPARdelta muscular injections and is therefore supportive.
The figures are too busy and of poor quality. The images of the fibers have to be amplified over 50 times to see details.
The reference formatting needs to be corrected.

Reviewer #2

(Remarks to the Author)
Daou and colleagues investigated the role of the metabokine BAIBA on skeletal muscle morphology and function, including in co-administration of exercise training as well as high fat diet-induced obesity and insulin resistance. The authors provide compelling data on the functional consequence of pharmacological supplementation of BAIBA and depletion by knock-down of the main biosynthetic enzyme. Thereby, intriguing novel insights into the biology and physiological as well as potential therapeutic relevance of this exercise-induced metabolite are presented. The following points might be considered to further improve the manuscript:

1.) It is surprising that for almost all in vivo analyses of skeletal muscle, M. soleus was chosen. The soleus muscle is a postural muscle, with a fiber type distribution heavily skewed towards type IIa and type I fibers, thus not representative of the function and fiber type distribution of many other muscle beds, in particular those that are predominantly engaged in mouse running behavior. Thus, to allow a broader discussion of the findings, at least some of the experiments should be repeated in other muscles with a more mixed fiber type.

2.) Based on the data presented in Figure 1i, muscle fiber hyperplasia leading to a higher number of total myofibers seems to be a major driver of increased muscle mass and hypertrophy. While hypertrophy of the soleus muscle has consistently been seen in endurance training paradigms in mice, this has mostly been attributed to fiber hypertrophy, not hyperplasia. Could the authors put this into perspective of training studies? Second, as mentioned, endurance training in most muscles will not drive hyperplasia, except for the soleus (at least with wheel running). How would the fiber type data (total number, Min Feret diameter,…) look in other muscles in the experiment shown in Figure 1? The fiber diameter data should also be displayed for each fiber type. This might help to interpret the findings.

3.) The gene expression analysis of the HFD experiment was the only dataset in which a BAIBA-dependent change in mitochondrial genes was reported. However, in all contexts, increased mitochondrial number and/or function was observed. How do the authors explain these results in absence of regulation of mitochondrial genes? How would mitochondrial biogenesis (or fission) be brought about in the absence of corresponding markers and pathways? Are mitochondrial proteins seen at higher levels? More light should be shed on this issue.

4.) In the exercise plus BAIBA experiment (Figure 2), ex vivo contractility was performed, yet the results of fatigability not displayed. This would be the main expectation based on the reported fiber type shift. Moreover, endurance training might not be expected to increase peak force, in particular at the highest frequencies. How do the authors reconcile this with the results in Figure 1 and 2?

5.) The lack of difference in running wheel behavior between the light and the dark phase for the control animals in Figure 1a-d is puzzling. Wouldn't a clearer difference be expected based on the nocturnal behavior of mice?

Reviewer #3

(Remarks to the Author)
This manuscript from Daou and colleagues details the interesting findings the the valine metabolite, b-aminoisobutyric acid (BAIBA), exerts several exercise-induced changes in muscle physiology. Included in this work are evidences of metabolic improvements in mice with BAIBI supplementation and human correlations of BAIBA following exercise. Mechanistically, the authors identify the involvement of PPARdelta (PPARd) into these effects. While the data are very interesting and potentially identify a major component of exercise-induced benefits, several concerns exist about the manuscript in its current state:

1) Overall, the manuscript is very difficult to follow. Several key details are missing from the text, and the figures are organized in a grid pattern that depicts the data in inconsistent graph styles and blurry IF images that are too small to discern detail (scale bars are also missing). Together, these issues make it difficult to accurately interpret the data. For example, the experiment pertaining to Figure 1 was not clear as to if this was an exercise study with prolonged running wheel access or an acute running wheel evaluation without appropriate acclimation sessions. Experiment 2, on the other hand is clearly a voluntary exercise study. The manuscript would be much improved if it were reorganized to effectively convey the information- for instance, a brief schematic of experimental design for each figure would clarify the study design.

2) There are several RNAseq datasets included in this study, however, all of these data are presented in supplemental figures with minimal analysis provide. Very important mechanistic information is likely to be found by more sophisticated analyses of these data, so it is recommended to enhance this work with better transcriptomic analyses. Also, are the RT-PCR results presented throughout the manuscript in agreement with the corresponding RNAseq data?

3) For the human myoblast studies, measures of proliferation and fusion are implied to represent exercise-like phenotypes. This is very misleading and not representative of any aspect of endurance exercise in vivo. Alternatively, these measures of myoblast behavior are more appropriately associated with muscle regeneration. In this context, it would be very interesting to

test how BAIBA affects muscle regeneration in vivo.

4) In the ABAT-depletion experiment (Figure 4), the decrease in exercise capacity and adaptability suggests mitochondria decrements are occurring as a result of the knockdown treatment. This may be independent of BAIBA production. Proper interpretation of this experiment requires knowing whether or not mitochondrial content is negatively affected before the start of exercise adaptation testing.

Version 1:

Reviewer comments:

Reviewer #1

(Remarks to the Author)
The authors have performed several new experiments in response to the reviewers' comments. The amount of data and number of new experiments is impressive. The human studies are more comprehensive. Experiments using the Abat knockout mice showing reduced performance, time to exhaustion, running to exhaustion, etc. further support that it is L-, not D-BAIBA that is responsible for the majority of positive effects on muscle. The authors are to be commended.

The authors finding that L, not D BAIBA is responsible for the majority of positive effects of the BAIBA mixture has been included in the abstract, however, nothing is mentioned about the enantiomers in the Introduction. It is suggested that the authors provide such information to the readers.

Line 90 page 5: It should be clarified that “BAIBA” is a mixture of the two enantiomers.
Line 99, page 5: An increase in number of wheel rotations was observed in the 6 mo old males which is in contrast to the studies of Vallejo et al (PMID 4104589) where L-BAIBA did not increase the number of wheel rotations although it improved muscle and bone function and phenotype. This suggests that the positive effects of L-BAIBA diminish with aging.

Reviewer #2

(Remarks to the Author)
The authors have addressed all of my points and suggestions in an adequate manner.

Reviewer #3

(Remarks to the Author)
The authors have put considerable effort towards addressing the concerns of all of the reviewers in this revised manuscript. I have no further concerns and commend the authors in their responsiveness. Well done!

Open Access This Peer Review File is licensed under a Creative Commons Attribution 4.0 International License, which

permits use, sharing, adaptation, distribution and reproduction in any medium or format, as long as you give appropriate credit to the original author(s) and the source, provide a link to the Creative Commons license, and indicate if changes were made.
In cases where reviewers are anonymous, credit should be given to 'Anonymous Referee' and the source.
The images or other third party material in this Peer Review File are included in the article’s Creative Commons license, unless indicated otherwise in a credit line to the material. If material is not included in the article’s Creative Commons license and your intended use is not permitted by statutory regulation or exceeds the permitted use, you will need to obtain permission directly from the copyright holder.
To view a copy of this license, visit https://creativecommons.org/licenses/by/4.0/

Response to Reviewers

Below please find our point-by-point response to the reviewers' comments. References to pages refer to the marked copy of our revised manuscript.

Reviewer 1

This manuscript shows that BAIBA can enhance the positive effects of exercise and improved metabolic and functional phenotype in a mouse model of obesity. The authors have used three mouse models (WT animals plus/minus VWR and BAIBA, a model of muscle injection of an antisense to 4-aminobutyrate aminotransferase, ABAT, and a high fat diet model), ex vivo human myocytes, and a human study (correlation of plasma BAIBA to VO2 peak) to determine various functions of BAIBA, a muscle metabolite.

We thank the reviewer for their constructive comments, which have helped to improve our manuscript.

It is not clear why the authors only used 6 week old males for the voluntary wheel running experiments. These are immature animals perhaps equivalent to teenagers. Mature mice at least 4-5 months when the musculoskeletal system is considered mature should be used. Otherwise the extrapolation to humans is not appropriate.

We agree with the reviewer that it is important to consider the effects of BAIBA on muscle phenotype and function at different stages of the life course, which we have achieved with our study. We not only used mice in the 6-12 week age range but also examined effects between 3.5 and 7 months old as we clarify below.

By 6 weeks of age C57BL6 mice are post-puberty[1, 2, 3] and sexually mature with "first matings" occurring (https://www.informatics.jax.org/silver/tables/table4-1.shtml). Muscle fibres in mice are post mitotic from 3 weeks of age[4, 5] with physiological muscle growth via hypertrophy continuing in young adulthood from 6 weeks, reaching maximum size by about 12-14 weeks[5]. Interestingly, this early adult phase corresponds well with known physical activity behaviours in humans in developed countries. Recent large population studies and surveys in the UK (Scottish Health Survey 2011; National Health Survey Statistics on Statistics on Obesity, Physical Activity and Diet, England, 2020) support the pattern that, on average, younger adults (16–24 years) exhibit higher levels of physical activity than other age groups (https://www.gov.scot/publications/scottish-health-survey-2011-volume-1-adults/pages/46/; https://digital.nhs.uk/data-and-information/publications/statistical/statistics-on-obesity-physical-activity-and-diet/england-2020/part-5-adult-physical-activity-copy.). Although direct matching of human age to murine age is imperfect, our use of 6 – 12 week old mice is interpreted as a similar age range[3].

In addition, we apologise if it was not clear from our original manuscript. The chow fed +/- BAIBA treated mice used as comparators to high fat diet-induced obese mice +/- BAIBA in **Figure 5 (page 11)** began BAIBA treatment at 14 weeks old (3.5 months) for a period of 14 weeks (age at completion 7 months) putting these mice in the age range recommended by the reviewer. We have revised our manuscript to now include a study design schematic to make this clearer (**Supplementary Figure 5c**). As shown in **Figure 5,** these older mice exhibit BAIBA-induced increases in wheel running, muscle mass (soleus, TA etc.)**,** muscle contractile function, muscle mitochondrial function and muscle mitochondrial content, phenocopying these effects observed in the younger mice.

Females also should be used for comparison.

We have added extensive additional data to the manuscript describing the effects of 6-week 100 mg/kg/day BAIBA treatment in female mice on **page 7** and new **Supplementary Figure 2**. We show that the effects of BAIBA in female mice phenocopy the effects observed in male mice, increasing voluntary exercise performance in free wheel running, muscle mass, muscle mitochondrial number and function, and soleus contractile function. The results are described in detail in the manuscript (**page 7**):

"*Next, we examined whether the BAIBA-induced effects exhibit sexual dimorphism. As for male mice, six week old female C57BL6/J mice were treated with 100 mg/kg/day BAIBA in drinking water for 6 weeks. BAIBA treated female mice exhibited significantly greater total free wheel running rotations (Supplementary Fig 2a), soleus and TA muscle mass (Supplementary Fig 2b), and soleus Myog, Myh7 and Myh2 expression (Supplementary Fig 2c). Examination of the mitochondrial phenotype using high-resolution respirometry in soleus from BAIBA-treated female mice indicated increased mitochondrial capacity (Supplementary Fig 2d) and content (complex IV respiratory activity) (Supplementary Fig 2e). Soleus force-frequency was also greater in BAIBA-treated female mice for both absolute and normalized contractile force (Supplementary Fig. 2f & 2g). BAIBA-treatment also increased soleus fatigue resistance in female mice (Supplementary Fig 2h). The effects of BAIBA on female mice phenocopy the effects in male mice and do not display sexual dimorphism*."

We do not observe sexual dimorphism in the effects of BAIBA in this experiment.

If there is an increase in the number of fibers, this suggests the muscles are immature and still developing. Muscle fibers in the mature muscle are post-mitotic.

We thank the reviewer for highlighting this important point. As mentioned above, murine muscle fibres are considered post-mitotic at 3 weeks of age[4, 5]. We conducted our experiments in mice at both 6 – 12 weeks (**Pages 5-7**, **Figure 1**) and 3.5 – 7 months of age (**Pages 11-12, Figure 5**). We examined the effect of BAIBA on chow fed mice used as comparators to high fat diet-induced obese mice and BAIBA-treated mice (Pages 11-12, **Figure 5),** which began BAIBA treatment at 14 weeks old (3.5 months) for a period of 14 weeks (age at completion 7 months). As shown in **Figure 5,** these older mice exhibit BAIBA-induced increases in wheel running, muscle mass (soleus, TA etc.)**,** muscle contractile function, muscle mitochondrial function and muscle mitochondrial content, consistent with these effects observed in the younger mice.

However, unlike in the 6 – 12 week old BAIBA-treated mice, the 3.5 month – 7 month old mice treated with BAIBA did not exhibit increases in soleus myofiber number. As the reviewer suggests this may relate to differential effects across the lifespan of the mice. We thank the reviewer for raising this and helping to identify this novel finding. Following the reviewer's comment we have edited our discussion to note this one non-congruent effect between younger and older mice (**pages 20-21**):

"*Not all effects of BAIBA on skeletal muscle directly phenocopy endurance exercise training. In 6 week old mice treated with BAIBA or L-BAIBA for 6 weeks there is an increase in myofiber number, suggesting hyperplasia. This phenotype was recapitulated in HSkMCs treated with BAIBA and highlighted by enrichment of myogenic terms across our RNAseq datasets. Interestingly, in older, healthy mice, treated with BAIBA from 3.5 months to 7 months old (comparators for our obesity-induced skeletal muscle dysfunction model) we do not observe an increase in fiber number, with BAIBA-induced muscle remodelling*

characterised specifically by the more canonical exercise training-like effects of fiber, mitochondrial and functional remodelling. This likely reflects differential effects of BAIBA across the life course. Murine skeletal muscle is considered post-mitotic at 3 weeks old[63, 64]. However, recent studies have suggested that a small population of satellite cells either remain non-quiescent in the skeletal muscle of young adult mice[65, 66], or can be driven out of quiescence by environmental cues[67] or endurance exercise training[68]. Interestingly, there is also evidence that particular endurance exercise training programmes may induce hyperplasia limited to the soleus[69].”

It is stated that the muscles in the mice were injected 2 times per week. This would definitely induce muscle damage.

As mentioned above, and within our manuscript, injections were made to the gastrocnemius as it lies proximal to the soleus, in which we performed the majority of assessments. When developing this methodology we performed proof-of-principle studies to ensure that the delivery method did not impact soleus function. 6 week old male C57BL6 mice received sham intramuscular injections (phosphate buffered saline) into both hindlimb gastrocnemius skeletal muscles twice a week for six weeks. These sham injected mice were compared with uninjected age-matched male C57BL6 control mice. Isolated soleus from the sham pbs injected and uninjected control mice was attached to a force transducer in an organ bath, stimulated with a supramaximal current and contractile properties were assessed in vitro using our established force-frequency and fatigue protocols employed within our manuscript. The results of these experiments are provided here for the benefit of the reviewer. Soleus force-frequency was not different between sham pbs-injected mice and uninjected controls for either absolute contractile force or soleus fatigue resistance:

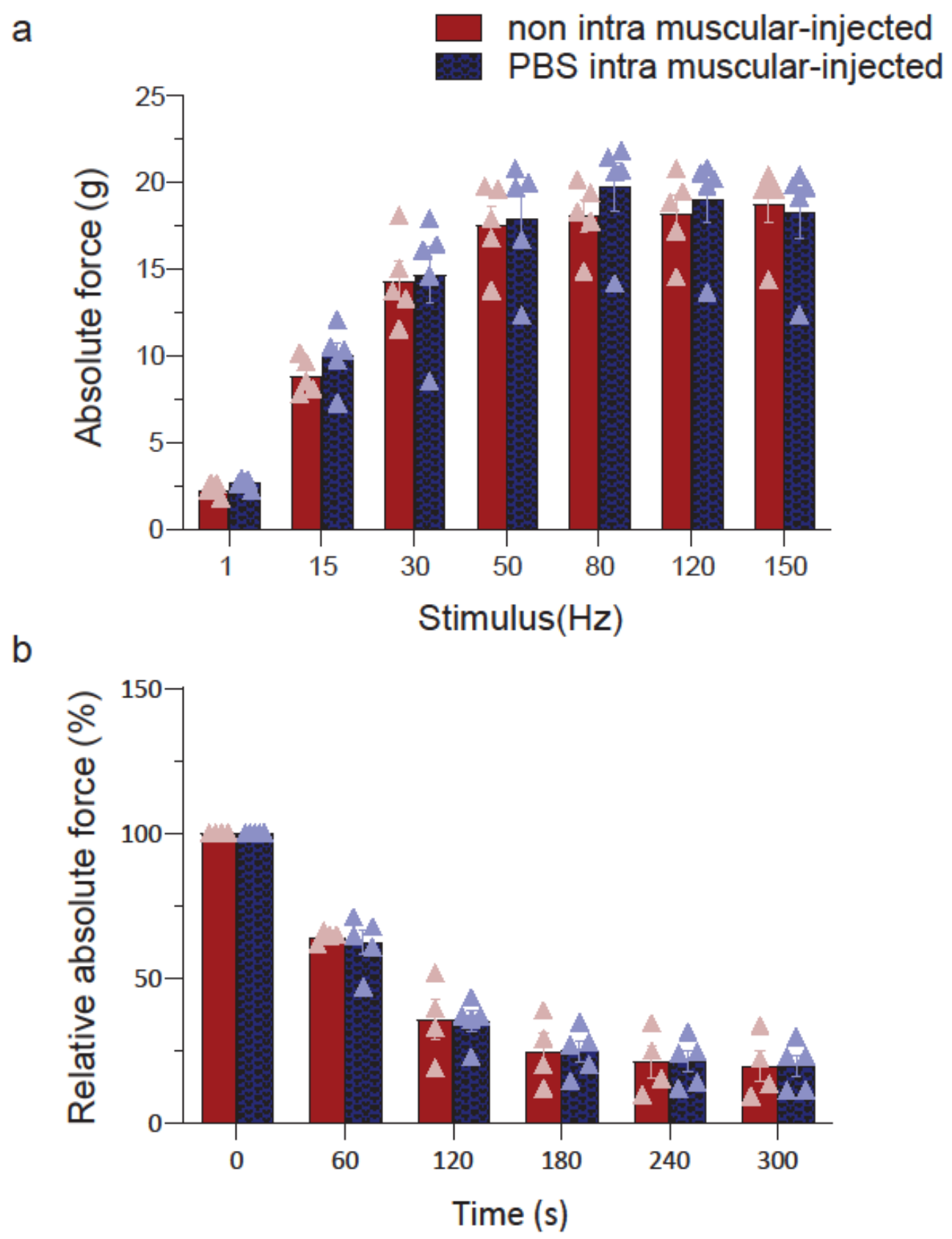

(**a**) Absolute ex vivo contractile force (g) under increasing stimuli (Hz) of the soleus from male C57BL6 mice receiving sham intramuscular injection of phosphate buffered saline into the hind limb twice per week for six weeks (blue) or age-matched non-injected controls (n = 5). (**b**) Relative absolute force as a percentage of the initial contractile force over 300 s to assess fatigue resistance in the soleus from male C57BL6 mice receiving sham intramuscular injection of phosphate buffered saline into the hind limb twice per week for six weeks (blue) or age-matched non-injected controls (n = 5).

Therefore, our injection protocol and methodology did not affect soleus contractile function, which would have been expected if muscle damage had occurred.

It is not clear which muscles are being injected.

In our methods section we state that the injections were made into the gastrocnemius muscle of the hindlimb (**Page 25**):

"*The exercised and unexercised groups were further subdivided into groups receiving intramuscular injections into both hindlimb gastrocnemius skeletal muscles…."*

To further increase clarity we have now edited our results section to describe this approach (**Page 17**):

"*These groups were further divided into groups receiving either scrambled short DNA antisense oligonucleotide (Gapmers) or short DNA antisense oligonucleotide against Abat, intramuscularly into the gastrocnemius muscles of both hind limbs twice a week to knockdown Abat expression*".

The gastrocnemius was targeted because it is the largest muscle of the hindlimb and lies directly above the soleus, allowing efficient local delivery. As we show, hindlimb skeletal muscle Abat expression was significantly reduced in both the soleus and gastrocnemius (**Figure 9a & 9b**).

What is the delivery vehicle for the antisense-virus or electroporation.

We selected GapmeRs and Accell siRNA as they represent a novel, relatively new, technology that can enter cells without the need for transfection reagents and are active in vivo without the need of formulation: **https://geneglobe.qiagen.com/us/product-groups/antisense-lna-gapmers; https://horizondiscovery.com/en/applications/rnai/self-delivering-accell-sirna#:~:text=A%20single%20reagent%20for%20guaranteed,in%20nearly%20any%20cell%20line.** This approach is supported by a number of key citations[6, 7 8, 9]***.***

To clarify this within our manuscript, we have edited our methods section to include (**Page 25**):

"*GapmeRs enter cells without the need for transfection reagents and are active in vivo without the need of formulation*[72,73,74]."

Were major muscles in both hindlimbs injected in order to observe a decrease in circulating BAIBA? These important details are missing.

As mentioned above intramuscular injections were made into both hindlimb gastrocnemius skeletal muscles. We have edited both the methods section and the results section of our manuscript to increase clarity:

(**Page 25**):

"*The exercised and unexercised groups were further subdivided into groups receiving intramuscular injections into both hindlimb gastrocnemius skeletal muscles…."*

To further increase clarity we have now edited our results section to describe this approach (**Page 17**):

"*These groups were further divided into groups receiving either scrambled short DNA antisense oligonucleotide (Gapmers) or short DNA antisense oligonucleotide against Abat, intramuscularly into the gastrocnemius muscles of both hind limbs twice a week to knockdown Abat expression*".

Another concern is the increase in activity in the animals injected with BAIBA as shown in Figure 1b and 2b. This would suggest that BAIBA is changing behavior that it is increasing wheel running. This change in behavior would need to be taken into account when interpreting the data.

We apologise if our data were not presented clearly as to show that BAIBA treatment did not alter behaviour. In the original version of our manuscript, we show that BAIBA treatment increases the total number of wheel rotations mice perform (**Page 5, Fig 1a in R1**), the speed at which the mice run (**Page 5, Fig 1b**), and the number of rotations mice perform per exercise interval (**Page 5, Fig 1c**). We have now also included more granular additional data from our free wheel running phenotyping data to show that BAIBA treatment does not affect the total number of exercise bouts the animals perform in either the Light or the Dark phase (**Page 5**, **Supplementary Figure 1j**) or the total average duration of all exercise bouts (**Page 5, Supplementary Figure 1k**). Therefore, when the animals perform free wheel running, BAIBA is increasing the capability of the mice to run at higher speeds and therefore the total rotations the mice make, without affecting behavioural measures such as the number of exercise bouts or the duration of these bouts. We have added this new data and edited our manuscript for further clarity (**Page 5**):

"However, BAIBA treatment does not affect the total number of exercise bouts the animals perform (Supplementary Fig 1j) or the total average duration of all exercise bouts (Supplementary Fig 1k). BAIBA enhances voluntary exercise performance in mice without affecting behavioural measures indicative of volition, such as the number or duration of exercise bouts."

This data is consistent with our phenotyping of the effects of BAIBA on baseline behaviour and activity. In our previous publication, we show that 100 mg/kg/day BAIBA does not affect mouse basal activity (ambulatory / locomotor)[10]. In this manuscript, we repeat this experiment and confirm the finding that BAIBA does not affect baseline activity with regard to ambulatory and locomotor behaviour (**Page 5**, **Supplementary Figures 1g & 1h**):

"*Basal activity and food intake were unaffected (Supplementary Figure 1g-i)."*

The human studies are very preliminary. There is no breakdown with regards to age or sex. What is the correlation is simply due to an increased muscle mass? Additional studies should be performed.

In response to the reviewer's comment, we have performed extensive additional human studies. We now include an acute aerobic exercise intervention study, which was performed

in our original volunteer population. We use LC-MS to measure the serum levels of the D- and L- isoforms of BAIBA independently, either at baseline rest or after a 45 min 60% VO_{2PEAK} treadmill inclined walking intervention (**Page 16, Figure 8**). We find that serum L-BAIBA at baseline significantly correlates with VO_{2PEAK} (**Page 16, Figure 8b**). Serum D-BAIBA does not significantly correlate with VO_{2PEAK} (**Page 16, Figure 8a**). Neither D nor L-BAIBA significantly correlated with age (**Page 16, Supplementary Fig 9a & 9b**).

Using the new data from our intervention approach, we found that both serum L-BAIBA and serum D-BAIBA were increased by acute aerobic exercise in our study (**Page 16**, **Figures 8c & 8d)**. This increase in serum BAIBA concentrations occurred for both male and female participants (**Page 16, Supplementary Figures 9 c-f**). Our human data is consistent with that of others that have shown that plasma BAIBA correlates with VO_{2PEAK}[11].

In further response to the reviewer's request for additional studies, we have explored the relationship of D-BAIBA and L-BAIBA with endurance exercise training in humans. We now include new analysis of D-BAIBA and L-BAIBA concentrations in the serum of healthy volunteers taken pre and post a 10 week (28 session; 60 min per session) cycle ergometer endurance exercise training programme[12]. The participants in this study exhibited a significant increase in VO_{2PEAK} (ml/kg/min) without increases in lean mass (assessed by DEXA) with exercise training programme completion[12]. In response to endurance exercise training the serum levels of D-BAIBA did not increase (**Page 16, Figure 8e**). However, serum concentrations of L-BAIBA were significantly increased by endurance exercise training (**Page 16-17, Figure 8f**).

Page 16-17:

"*We investigated the relationship between circulating levels of L-BAIBA and D-BAIBA and both acute aerobic endurance exercise and fitness in humans. Maximal aerobic capacity was assessed in sixty human volunteers (Supplementary Table 1) using a peak pulmonary oxygen uptake (VO_{2PEAK}) cardiopulmonary exercise test on a treadmill. At a secondary study visit resting fasted serum was taken from the volunteers prior to undergoing an acute endurance exercise treadmill intervention for 45 minutes at 60% $VO2_{PEAK}$. Immediately at exercise completion a second serum sample was taken to assess changes in response to acute aerobic exercise. L-BAIBA and D-BAIBA serum concentrations both at baseline and immediately after aerobic exercise were measured by LC-MS analysis in the volunteers. Serum L-BAIBA and D-BAIBA did not significantly correlate with age in our study population (Supplementary Fig 9a & 9b). Baseline resting D-BAIBA serum concentration did not correlate with VO_{2PEAK} (Fig. 8a). However, baseline resting serum L-BAIBA was significantly positively correlated with $VO2_{PEAK}$ ($r = 0.26$, p-value = 0.046) (Fig. 8b). These data suggest circulating serum L-BAIBA concentration associates with aerobic fitness in humans.*

Next, we compared the levels of D-BAIBA and L-BAIBA in the serum at baseline and immediately after the acute exercise treadmill intervention for 45 minutes at 60% VO_{2PEAK}. Both D-BAIBA and L-BAIBA were increased in the serum in response to acute aerobic exercise (Fig 8c & 8d). Exercise-induced increases in serum D- and L-BAIBA occurred in both male and female participants (Supplementary Fig 9c – 9f). Acute aerobic exercise induces increases in circulating L-BAIBA and D-BAIBA levels in humans.

Serum L-BAIBA increases in response to endurance exercise training in humans

We then assessed the relationship between plasma D-BAIBA and L-BAIBA and endurance exercise training in 33 human participants (Supplementary Table 2). We analyzed D-BAIBA and L-BAIBA concentrations in the plasma from volunteers taken pre and post a 10-week aerobic exercise cycle ergometer-based training program[47]. Exercise training increased

volunteer VO_{2PEAK}[47]. LC-MS analysis demonstrated that circulating levels of D-BAIBA in blood plasma of volunteers did not increase in response to exercise training (Fig. 8e). Again, L-BAIBA correlated to VO_{2PEAK} at baseline in this independent group of volunteers ($r = 0.49$; $p = 0.004$) (Fig. 8f). L-BAIBA plasma concentration was increased by endurance exercise training (Fig. 8g). Endurance exercise training increased circulating levels of L-BAIBA in humans."

In addition, we have previously published data showing that plasma BAIBA increases in response to a 20 week cycle ergometer-based endurance exercise training programme which increased VO_{2PEAK}, conducted as part of the HERITAGE exercise study and adjusted for age and sex[10, 13].

The authors have failed to acknowledge that there are two enantiomers of BAIBA with different functions and different tissue sources. This is important because the two enantiomers though signaling through the same receptor can have different effects through distinct signaling pathways. L but not D BAIBA has been shown to be released by contracted murine muscle. The commercial source of BAIBA used for all experiments is a mixture of both enantiomers and the LC-MS used for the quantitation of BAIBA only recognizes total BAIBA and not the two enantiomers.

We thank the reviewer for this important comment. In response, we have performed extensive additional experiments to clarify the effects of the D- and L- enantiomers of BAIBA on 1) voluntary exercise responses in mice and 2) mouse muscle phenotype and function. We treated mice for 6 weeks with either D-BAIBA or L-BAIBA independently, finding that the L-BAIBA enantiomer is responsible for the effects of BAIBA on voluntary exercise response, and muscle fiber, metabolic and functional remodelling (**page 14, Figure 7**):

"*Physiologically BAIBA is present as two distinct D- and L- enantiomers with L-BAIBA previously identified as secreted from contracting skeletal muscle[20]. Therefore, we examined the effects of D-BAIBA and L-BAIBA enantiomers independently. C57BL6/J mice were either treated with 100 mg/kg/day L-BAIBA or D-BAIBA in drinking water for 6 weeks. Plasma L-BAIBA (Fig 7a) and D-BAIBA (Fig 7b) concentrations were increased in their respective treatment groups. At 6 weeks of treatment, mice were placed in a Combined Laboratory Animal Monitoring System to determine physiological phenotypes. L-BAIBA increased oxygen consumption and energy expenditure compared with D-BAIBA and control mice (Supplementary Fig 7a & 7b). Food intake and basal activity were not affected by D-BAIBA or L-BAIBA treatment (Supplementary Fig. 7c & 7d). As previously, we used free wheel running analysis, preceded by a 3-day habituation period, to examine voluntary exercise performance in these mice. L-BAIBA treated mice had greater total wheel rotations (Fig. 7c), running speed (Fig. 7d) and number of rotations per exercise interval (Fig. 7e). However, L-BAIBA and D-BAIBA treatment does not affect the total number of exercise bouts (Supplementary Fig 7e) or the total duration of all exercise bouts (Supplementary Fig 7f). L-BAIBA and not D-BAIBA enhances voluntary exercise performance in mice.*

We examined the effects of the distinct BAIBA enantiomers on skeletal muscle phenotype. L-BAIBA, but not D-BAIBA enhanced soleus muscle mass (Fig 7f). Using immunofluorescence we determined the effect of L-BAIBA and D-BAIBA on soleus muscle fiber-type composition (Fig 7g-7k). L-BAIBA but not D-BAIBA significantly increased the total number of fibers in soleus (Fig. 7h). This effect was primarily driven by a significant increase in type IIa fibers in the soleus of L-BAIBA treated mice (Fig. 7i). L-BAIBA specifically increased the diameter of type I fibers (Fig. 7j). Proportionally, D-BAIBA reduced type I fiber content in the soleus with L-BAIBA inducing fiber remodelling towards a higher percentage of type IIa fibers (Fig 7k).

Next, we investigated the effect of the D-BAIBA and L-BAIBA enantiomers on skeletal muscle mitochondrial phenotype. Using high-resolution respirometry, we determined that L-BAIBA, and not D-BAIBA, increased soleus mitochondrial electron transport chain (ETC) coupled complex I-mediated oxidative phosphorylation (Fig. 7l). Soleus complex I and II succinate-stimulated respiration was higher in L-BAIBA-treated, but not significantly different in D-BAIBA-treated, mice (Fig. 7l). Maximal uncoupled ETC respiration did not differ from control in D-BAIBA treated mice, but was increased in the soleus of L-BAIBA-treated mice (Fig. 7l). We then used complex IV respiratory activity [26] to suggest that only L-BAIBA increased soleus mitochondrial content (Fig.7m). We employed immunoblotting to identify that L-BAIBA treatment increased the protein levels of mitochondrial electron transport respiratory complexes (complex II, II, V) in soleus (Fig 7n; Supplementary Fig 7g) as well as EDL (complexes II, IV and V) (Supplementary Fig 7h & 7i). Interestingly, D-BAIBA also exhibited the capacity to increase expression of complexes II and V in soleus and II, III and V in EDL. L-BAIBA increases mitochondrial content and respiratory capacity in skeletal muscle.

We then examined whether D- and L-BAIBA-treated mice exhibited improved soleus muscle contractile function using our force-frequency and fatigue protocols[27]. L-BAIBA induced greater soleus force-frequency in mice for both absolute and normalized contractile force (Fig. 7o & 7p). Although to a lesser extent than L-BAIBA, D-BAIBA also increased absolute and normalized contractile force (Fig 7o & 7p). However, only L-BAIBA-treatment, and not D-BAIBA, increased soleus fatigue resistance, consistent with the effects of L-BAIBA on mitochondrial function and number (Fig. 7q).”

As mentioned above, we now also include two new human interventional exercise studies. The first explores the effect of acute aerobic exercise and the second chronic aerobic exercise training on circulating D- and L-BAIBA in humans (**Page 16-17, Figure 8**):

“*We investigated the relationship between circulating levels of L-BAIBA and D-BAIBA and both acute aerobic endurance exercise and fitness in humans. Maximal aerobic capacity was assessed in sixty human volunteers (Supplementary Table 1) using a peak pulmonary oxygen uptake (VO_{2PEAK}) cardiopulmonary exercise test on a treadmill. At a secondary study visit resting fasted serum was taken from the volunteers prior to undergoing an acute endurance exercise treadmill intervention for 45 minutes at 60% VO_{2PEAK}. Immediately at exercise completion a second serum sample was taken to assess changes in response to acute aerobic exercise. L-BAIBA and D-BAIBA serum concentrations both at baseline and immediately after aerobic exercise were measured by LC-MS analysis in the volunteers. Serum L-BAIBA and D-BAIBA did not significantly correlate with age in our study population (Supplementary Fig 9a & 9b). Baseline resting D-BAIBA serum concentration did not correlate with VO_{2PEAK} (Fig. 8a). However, baseline resting serum L-BAIBA was significantly positively correlated with VO_{2PEAK} ($r = 0.26$, p-value = 0.046) (Fig. 8b). These data suggest circulating serum L-BAIBA concentration associates with aerobic fitness in humans.*

Next, we compared the levels of D-BAIBA and L-BAIBA in the serum at baseline and immediately after the acute exercise treadmill intervention for 45 minutes at 60% VO_{2PEAK}. Both D-BAIBA and L-BAIBA were increased in the serum in response to acute aerobic exercise (Fig 8c & 8d). Exercise-induced increases in serum D- and L-BAIBA occurred in both male and female participants (Supplementary Fig 9c – 9f). Acute aerobic exercise induces increases in circulating L-BAIBA and D-BAIBA levels in humans.

Serum L-BAIBA increases in response to endurance exercise training in humans

We then assessed the relationship between plasma D-BAIBA and L-BAIBA and endurance exercise training in 33 human participants (Supplementary Table 2). We analyzed D-BAIBA and L-BAIBA concentrations in the plasma from volunteers taken pre and post a 10-week

aerobic exercise cycle ergometer-based training program[47]. Exercise training increased volunteer VO2PEAK 47. LC-MS analysis demonstrated that circulating levels of D-BAIBA in blood plasma of volunteers did not increase in response to exercise training (Fig. 8e). Again, L-BAIBA correlated to VO2PEAK at baseline in this independent group of volunteers (r = 0.49; p = 0.004) (Fig. 8f). L-BAIBA plasma concentration was increased by endurance exercise training (Fig. 8g). Endurance exercise training increased circulating levels of L-BAIBA in humans."

We have also repeated key human primary skeletal myocyte experiments including RNAseq analysis specifically using L-BAIBA treatment, finding that L-BAIBA specifically induces the markers of differentiation in human primary myocytes (**page 15-16, Supplementary Figure 8**):

"*We observed that the L-BAIBA, rather than the D-BAIBA, enantiomer was primarily responsible for improved muscle phenotype and function in our mouse models. Therefore, we examined the effect of L-BAIBA on markers of HSkMC differentiation in vitro. HSkMCs were seeded and immediately treated with 10µM L-BAIBA. L-BAIBA increased expression of key regulatory genes for skeletal muscle differentiation 72h post-seeding including paired box 7 (PAX7), MYF5 and MYF6 (Supplementary Fig. 8a). Then, we treated myoblasts with 10µM L-BAIBA during a six day in vitro differentiation period. For a direct assessment of differentiation and myoblast fusion we used immunohistochemistry to stain the L-BAIBA-treated and differentiated myotubes for muscle myosin (MF20), nuclei (Hoechst) and the marker of differentiation, myogenin (Supplementary Fig. 8b). L-BAIBA increased the differentiation index (Supplementary Fig. 8c), and fusion index (Supplementary Fig. 8d) of the HSkMCs. We investigated the molecular phenotype induced by L-BAIBA in HSkMCs with RNAseq in 10µM L-BAIBA-treated HSKMCs compared with controls (https://www.ebi.ac.uk/arrayexpress/experiments/E-MTAB-14746). Our analysis identified 460 unique and significantly DEGs between L-BAIBA-treated and control myocytes, with 247 DEGs up- and 213 DEGs down-regulated (Supplementary Fig 8e). Using GSEA28 of the significant DEGs searching against the MSigDB Molecular Signatures Database29 (Supplementary Fig 8f), we found that the top 2 most enriched terms with L-BAIBA treatment, mesenchymal remodelling (1.68e-54) and myogenesis (4.89e-28) were consistent with our in vivo results for BAIBA induced transcriptional processes in muscle of both our HFD-fed mice and our 6 week BAIBA treatment model and our previous study of human primary myotubes."*.

In addition, we have incorporated a published LC-MS method for the measurement of both the D- and L- enantiomers of BAIBA (**Page 34**), which we have applied in our human exercise studies, our studies of the separate enantiomers of BAIBA in mice and in our ABAT knockdown studies in mice.

As ABAT play a major role in the production of L-BAIBA, the mouse with the reduction of ABAT in muscle is showing an inhibition of L, not D-BAIBA secretion.

We agree with the reviewer and have included new data in which both L-BAIBA and D-BAIBA concentrations were measured in the plasma of our ABAT knockdown mouse model. ABAT knockdown reduced circulating L-BAIBA concentration without affecting D-BAIBA (**page 17, Figure 9c, Supplementary Fig 10c**):

"LC-MS analysis of plasma from the mice indicated Abat knockdown impaired the exercise-induced increase in circulating L-BAIBA concentration (Fig 9c) without affecting D-BAIBA concentration (Supplementary Figure 10c)."

The RNAseq data would most likely be different if only L or only D-BAIBA were given to the animals.

As mentioned above we now include new data examining the effects of L-BAIBA and D-BAIBA given independently to mice for 6 weeks. The effects of L-BAIBA phenocopy the effects observed previously using the enantiomeric mix whereas D-BAIBA had a very limited effect on any of the physiological or metabolic endpoints examined systemically or in the muscle of the animals.

Interestingly, in new data provided in our revision, we performed RNAseq analysis on human primary myotubes treated with L-BAIBA (**Page 15-16, Supplementary Figure 8**). Using Enrichr to perform Gene Set Enrichment Analysis (GSEA)[17] of the significantly differentially expressed genes in our RNAseq data sets of both L-BAIBA (**Page 15-16, Supplementary Figure 8)** and enantiomeric BAIBA (**Page 9, Figure 4**) gave very consistent results with terms related to mesenchymal transition and myogenesis.

Results of BAIBA treatment of human primary skeletal myotubes (**page 9, Figure 4j**):

"*Performing Gene Set Enrichment Analysis (GSEA)[28] of the significantly differentially expressed genes in BAIBA treated human skeletal myotubes, again using the MSigDB Molecular Signatures Database[29], identifies that BAIBA induces transcriptional processes consistent with our observations in vivo and indicative of skeletal muscle structural remodelling including mesenchymal remodelling (adjusted p-value 1.63e-53), and myogenesis (adjusted p-value 3.87e-13) (Fig 4j).*"

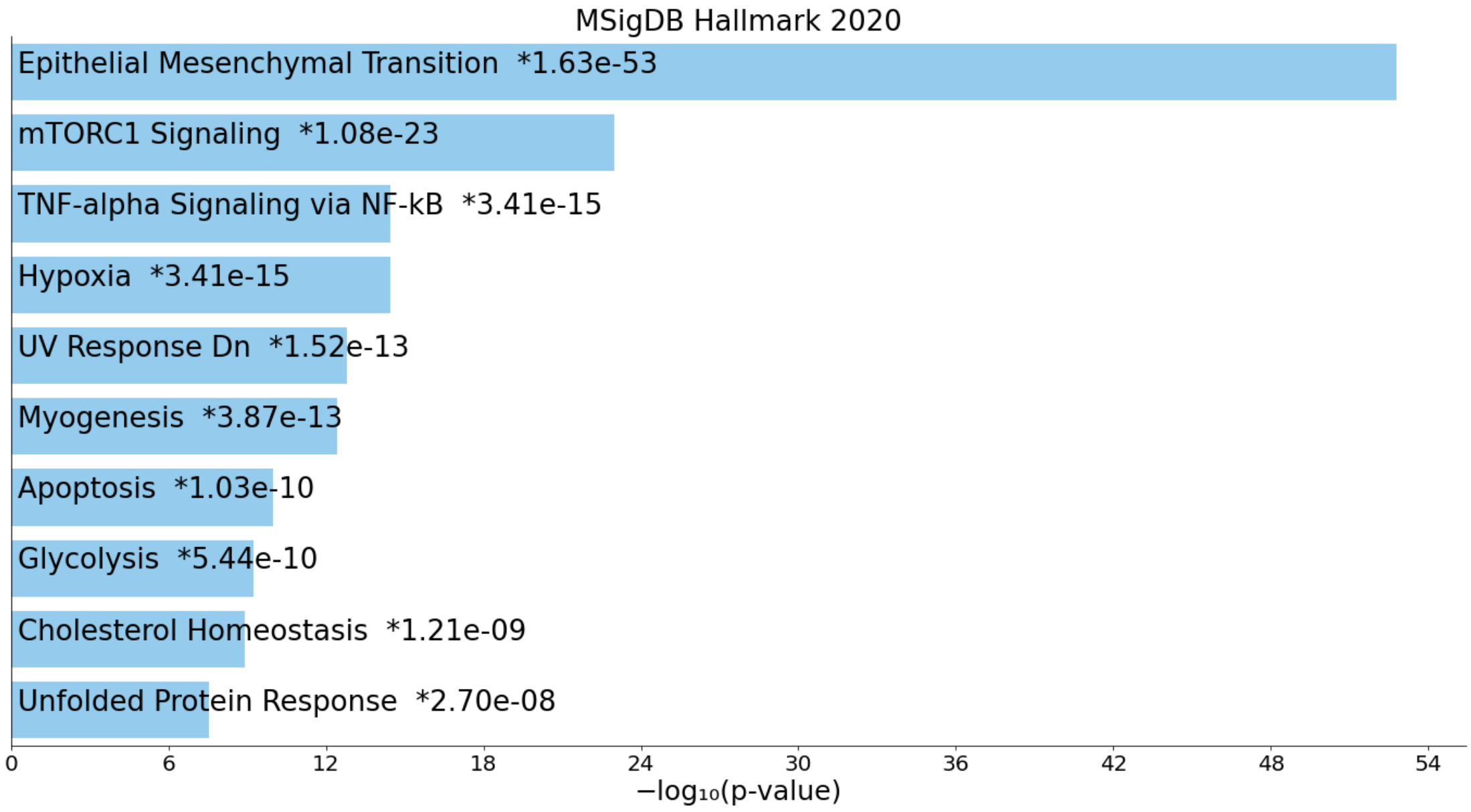

Results of L-BAIBA treatment of human primary skeletal myotubes (**page 15-16, Supplementary Fig 8f**):

"*Using GSEA[28] of the significant DEGs searching against the MSigDB Molecular Signatures Database[29] (Supplementary Fig 8f), we found that the top 2 most enriched terms with L-BAIBA treatment, mesenchymal remodelling (1.68e^{-54}) and myogenesis (4.89e^{-28}) were consistent with our in vivo results for BAIBA induced transcriptional processes in muscle of both our HFD-fed mice and our 6 week BAIBA treatment model and our previous study of human primary myotubes*."

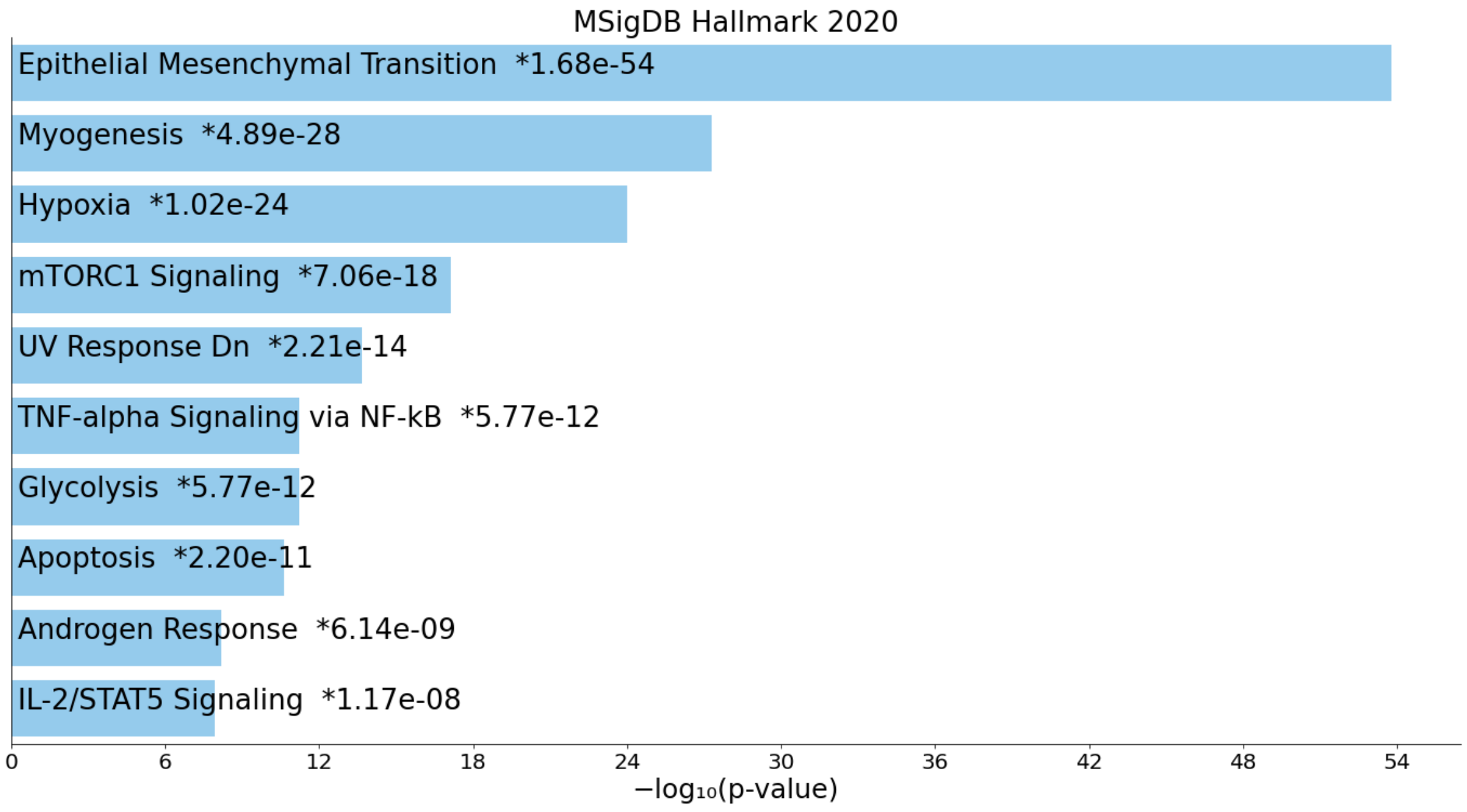

The references provided for the safe dosing of BAIBA only used L, not D-BAIBA. There are no references to date for the dosing of D-BAIBA.

We thank the reviewer for making this important point, which we have clarified in the text (**page 19**):

"*The L-enantiomer of BAIBA is safe, doseable and orally available in rodent models and in humans* [52, 53]."

In this study, a correlation of total BAIBA circulating levels correlated with aerobic exercise capacity. Previous published studies have provided a correlation of either L or D BAIBA with physical performance PMID: 37821627. In this study the significant correlation was with D-BAIBA and physical performance. A discussion of these results could be included.

As mentioned above, we now include extensive additional human exercise studies of our own investigating the response of circulating concentrations of D-BAIBA and L-BAIBA to both acute aerobic exercise intervention and a chronic exercise training programme.

As we outline above, we now include an acute aerobic exercise intervention study, which was performed in our original volunteer population. We use LC-MS to measure the serum levels of the D- and L- isoforms of BAIBA independently, either at baseline rest or after a 45 min 60% VO_{2PEAK} treadmill inclined walking intervention (**Page 16** We find that serum L-BAIBA at baseline significantly correlates with VO_{2PEAK} (**Page 16, Figure 8b**). Serum D-BAIBA does not significantly correlate with VO_{2PEAK} (**Page 16, Figure 8a**). Neither D nor L-BAIBA significantly correlated with age (**Page 16, Supplementary Fig 9a & 9b**).

Using the intervention approach, we found that both serum L-BAIBA and serum D-BAIBA were increased by acute aerobic exercise in our study (**Page 16, Fig 8c & 8d**). This increase in serum BAIBA concentrations occurred for both male and female participants

(**Supplementary Fig 9 c-f).** Our human data is consistent with that of others that have shown that plasma BAIBA correlates with VO_{2PEAK}[11].

We have also now explored the relationship of D-BAIBA and L-BAIBA with endurance exercise training in humans. We now include new analysis of D-BAIBA and L-BAIBA concentrations in the serum of healthy volunteers taken pre and post a 10 week (28 session; 60 min per session) cycle ergometer endurance exercise training programme[12]. The participants in this study exhibited a significant increase in VO_2max (ml/kg/min) without increases in lean mass (assessed by DEXA) with exercise training programme completion[12]. In response to endurance exercise training the serum levels of D-BAIBA did not increase (**Page 16, Fig 8e**). However, serum concentrations of L-BAIBA were significantly increased by endurance exercise training (**Page 17, Fig 8g**).

It would be important to go into more depth in signaling mechanisms. One easy experiment to perform is to determine if muscle expresses the Mas related G Protein Receptor type D, MRGPRD. There are several publications that have identified signaling pathways for both L and D BAIBA. It would be important to determine what these are in muscle.

We thank the reviewer for their helpful suggestion. In our original manuscript we presented extensive in vivo and in vitro data highlighting the mechanistic requirement for the nuclear receptor *PPARδ* in transducing BAIBA's effects in skeletal muscle and on systemic effects of exercise response (**Page 13**, **Figure 6**). In response to the reviewer's comment , we have now performed additional experiments using our established siRNA-mediated gene silencing approaches[19, 20] to target MRGPRD in human primary skeletal myocytes co-treated with L-BAIBA. Using this approach we have produced new data and determined that L-BAIBA requires expression of MRGPRD to induce the expression of markers of myocyte differentiation and oxidative fiber types in human primary myocytes (**Page 18, Figure 10**) . We also identify that D-BAIBA does not induce expression of the differentiation and oxidative fiber type markers in human primary myocytes (**Page 18, Figures 10b-e**):

"*We further explored the mechanisms for BAIBA-induced regulation of skeletal muscle gene expression. We previously determined that BAIBA increased expression of markers of terminal differentiation towards oxidative muscle fibers, MYH7 and MYH2, and regulatory genes for skeletal muscle differentiation MYOD1, and MYOG in human primary myocytes. In osteocytes, BAIBA activity requires the cell surface G-protein coupled receptor, Mas-related G protein-coupled receptor D (MRGPRD)[20]. To determine whether MRGPRD is required to transmit BAIBA signalling in human myocytes we used siRNA-mediated silencing to knockdown MRGPRD expression in human primary myocytes by 89% (Fig 10a) with and without L-BAIBA (10μM) treatment during a six day in vitro differentiation period. Knockdown of MRGPRD impaired L-BAIBA induced expression of MYOD1 (Fig 10b), MYOG (Fig 10c), MYH7 (Fig 10d) and MYH2 (Fig 10e). Cells were also treated with D-BAIBA (10μM), and consistent with our in vivo study, D-BAIBA had no effect on myocyte gene expression (Fig 10b-e). BAIBA-induced expression of markers of myocyte differentiation and oxidative fibers requires the cell surface G-protein coupled receptor MRGPRD in human primary myocytes.*"

Minor concerns:

What is the specificity of the PPARdelta inhibitor used GW3787 also known as GSK3787? It appears to also be a PPARbeta inhibitor see PMID: 20516370. The

outcomes with this inhibitor is replicated with siPPARdelta muscular injections and is therefore supportive.

GW3787 is an irreversible antagonist of PPARδ[21]. It exhibits high selectivity in comparison to the other PPAR isoforms PPAR-gamma (PPARγ) and PPARalpha (PPARα)[21]. Due to historical anachronism in the literature PPAR-delta (PPARδ) is also known as PPAR-beta (PPARβ)**.** However, they refer to the same peroxisome proliferator-activated receptor isoform, which is encoded by the *PPARD* gene. Therefore, the citation referenced by the reviewer (PMID: 20516370) detailing GW3787's PPAR-beta inhibitory action is consistent with our use as a PPAR-delta inhibitory tool in this study. In addition, as the reviewer states, we have replicated this result using siRNA against PPARdelta in both the primary human muscle cells and in mice in vivo.

The figures are too busy and of poor quality.

Due to the need to compress the Figures to perform the original upload to the journal the quality of the figures (and especially the histological images) was adversely affected. We have revised all main figures to improve quality. We have followed journal-specific requirements with graph selection using bar graphs with individual data points plotted when sample size in less than 10 and box plots showing data population distribution when samples size is 10 or greater. We have revised figure style to ensure consistency.

The images of the fibers have to be amplified over 50 times to see details.

We have enlarged our fiber images and improved the quality of these that were unfortunately impacted by the compression process.

The reference formatting needs to be corrected.
We have checked the referencing style. These are now presented in Nature Communications format as outlined on the Nature Communications website.

Reviewer #2 (Remarks to the Author):

Daou and colleagues investigated the role of the metabokine BAIBA on skeletal muscle morphology and function, including in co-administration of exercise training as well as high fat diet-induced obesity and insulin resistance. The authors provide compelling data on the functional consequence of pharmacological supplementation of BAIBA and depletion by knock-down of the main biosynthetic enzyme. Thereby, intriguing novel insights into the biology and physiological as well as potential therapeutic relevance of this exercise-induced metabolite are presented. The following points might be considered to further improve the manuscript:

We thank the reviewer for their support and insightful comments that by addressing we feel have improved our manuscript.

1.) It is surprising that for almost all in vivo analyses of skeletal muscle, M. soleus was chosen. The soleus muscle is a postural muscle, with a fiber type distribution heavily skewed towards type IIa and type I fibers, thus not representative of the function and fiber type distribution of many other muscle beds, in particular those that are predominantly engaged in mouse running behavior. Thus, to allow a broader discussion of the findings, at least some of the experiments should be repeated in other muscles with a more mixed fiber type.

We appreciate the reviewer's point regarding the value of determining effects of BAIBA across other skeletal muscles with differing fiber type composition. Our focus on soleus was due to its high degree of type 1 fibers (~20% in mice, similar to human mixed muscles) and as a key example of an endurance specific muscle. Moreover, soleus is highly sensitive to pathological conditions such as disuse atrophy and importantly obesity/diabetes-induced muscle dysfunction (diabetic myopathy), which we also explore in the manuscript. In addition, technically we are limited to examining either soleus or EDL for contractile functional analysis methodologies due to their tendon-tendon isolated preparations.

As recommended by the reviewer we have generated new data examining some aspects of the phenotype observed in soleus muscle in other muscles with either a more type II predominant fiber compostion (EDL) and tibialis anterior (TA) or mixed fiber type composition (gastrocnemius).

By generating new data, we now show that, as well as in soleus, BAIBA treatment of mice for 6 weeks also increases markers of mitochondrial number and function in EDL and gastrocnemius including:

i) increasing EDL and gastrocnemius citrate synthase activity (**Page 6, Supplementary Fig 1s**):

"*Citrate synthase activity, a marker of mitochondrial density and TCA cycle flux, was higher in the soleus (Fig. 1m), EDL and gastrocnemius (Supplementary Fig 1s) of BAIBA-treated mice compared with controls."*

ii) and increasing EDL and gastrocnemius mitochondrial electron transport chain respiratory complexes I-V protein expression (**page 6, Supplementary Fig 1t**):

"*Using immunoblotting we identify that BAIBA treatment increased the protein levels of all mitochondrial electron transport respiratory complexes (complex I – V) in soleus (Fig 1q; Supplementary Fig 1t) as well as EDL and gastrocnemius muscles (Supplementary Fig 1t)."*

Functionally, with the inclusion of new data, we now also show that BAIBA treatment improves the contractile fatigue resistance of EDL in mice treated with BAIBA for 6 weeks (**Page 7, Supplementary Fig 1u**):

"*As BAIBA increased both mitochondrial respiratory complex expression and citrate synthase activity in EDL, we investigated whether the contractile effects on soleus translated to EDL given its greater proportion of type II fibers. EDL was examined with the contractile fatigue protocols. Consistently BAIBA also increased EDL fatigue resistance (Supplementary Figure 1u).*"

We also now include data examining the effect of BAIBA on remodelling of other muscles of mixed fiber type. We have added data showing that 6 weeks of BAIBA treatment in mice increases TA and gastrocnemius mass (**page 5, Supplementary Fig 1l**):

"*BAIBA enhanced soleus (Fig 1d), tibialis anterior (TA) and gastrocnemius (Supplementary Fig. 1l) muscle mass.*"

In newly included data, we use our immunofluorescence approaches to evaluate the effect of BAIBA on muscle fiber type remodelling in gastrocnemius of mice treated with BAIBA for 6 weeks (**page 5-6, Supplementary Figures 1n-r**). Interestingly, BAIBA induced fiber-remodelling in the gastrocnemius increasing the number of type IIa fibers and increasing the myofiber diameter and specifically the hybrid type I/IIx and type IIx fibers.

"*We then applied our immunofluorescence approach to determine the effect of BAIBA on the mixed fiber composition of the gastrocnemius muscle (Supplementary Fig 1n). BAIBA did not significantly increase the total myofiber number in gastrocnemius (Supplementary Fig 1o). However, BAIBA induced fiber remodelling, significantly increasing the number of fatigue resistant type IIa fibers in gastrocnemius (Supplementary Fig 1p). BAIBA also significantly increased myofiber diameter (Supplementary Fig 1q) with a specific increase in the diameter of type I/IIx hybrid fibers (Supplementary Fig 1r).*

2.) Based on the data presented in Figure 1i, muscle fiber hyperplasia leading to a higher number of total myofibers seems to be a major driver of increased muscle mass and hypertrophy. While hypertrophy of the soleus muscle has consistently been seen in endurance training paradigms in mice, this has mostly been attributed to fiber hypertrophy, not hyperplasia. Could the authors put this into perspective of training studies?

This is an important point, which we thank the reviewer for highlighting. We agree that muscle hyperplasia would not be considered a canonical response to endurance exercise training. Muscle adaptation to exercise training is a complex physiological response regulated by multiple physical stimuli and molecular responses. We would not necessarily expect BAIBA treatment alone to exactly phenocopy exercise training.

The hyperplasia response commented upon by the reviewer is particularly worthy of note. Murine muscle fibres are considered post-mitotic at 3 weeks of age[4, 5]. We conducted our experiments in mice at both 6 – 12 weeks and 3.5 – 7 months of age. Alongside the 6 – 12 week old mice, we also examined the effect of BAIBA on chow fed mice used as comparators to high fat diet-induced obese mice and BAIBA-treated mice in **Page 11-12 Figure 5,** which began BAIBA treatment at 14 weeks old (3.5 months) for a period of 14 weeks (age at completion 7 months). As shown in **Figure 5,** these older mice exhibit BAIBA-induced increases in wheel running, muscle mass (soleus, TA etc.)**,** muscle contractile function, muscle mitochondrial function and muscle mitochondrial content, consistent with

both the effects of endurance exercise training and these same effects observed in the younger (6 – 12 week) mice (**Page 5-7, Figure 1**).

However, unlike in the 6 – 12 week old (**Page 5-7, Figure 1**) BAIBA-treated mice, the 3.5 month – 7 month old mice treated with BAIBA (**Page 11-12 Figure 5)** did not exhibit increases in soleus myofiber number (**Page 11, Figure 5d**). We therefore suggest this may relate to differential effects across the lifespan of the mice. Following the reviewers comment we have edited our discussion to note this one non-congruent effect between both younger and older mice and between BAIBA-treatment and canonical effects of endurance exercise training (**page 20-21**):

"*Not all effects of BAIBA on skeletal muscle directly phenocopy endurance exercise training. In 6 week old mice treated with BAIBA or L-BAIBA for 6 weeks there is an increase in myofiber number, suggesting hyperplasia. This phenotype was recapitulated in HSkMCs treated with BAIBA and highlighted by enrichment of myogenic terms across our RNAseq datasets. Interestingly, in older, healthy mice, treated with BAIBA from 3.5 months to 7 months old (comparators for our obesity-induced skeletal muscle dysfunction model) we do not observe an increase in fiber number, with BAIBA-induced muscle remodelling characterised specifically by the more canonical exercise training-like effects of fiber, mitochondrial and functional remodelling. This likely reflects differential effects of BAIBA across the life course. Murine skeletal muscle is considered post-mitotic at 3 weeks old[63, 64]. However, a number of recent studies have suggested that a small population of satellite cells either remain non-quiescent in the skeletal muscle of young adult mice[65, 66], or can be driven out of quiescence by environmental cues[67] or endurance exercise training[68]. Interestingly, there is also evidence that particular endurance exercise training programmes may induce hyperplasia limited to the soleus[69]."*

Second, as mentioned, endurance training in most muscles will not drive hyperplasia, except for the soleus (at least with wheel running). How would the fiber type data (total number, Min Feret diameter,…) look in other muscles in the experiment shown in Figure 1? The fiber diameter data should also be displayed for each fiber type. This might help to interpret the findings.

As mentioned above, and in response to the reviewer's comment, in newly included data, we use our immunofluorescence approaches to evaluate the effect of BAIBA on muscle fiber type remodelling in gastrocnemius of mice treated with BAIBA for 6 weeks (**page 5-6, Supplementary Figures 1n – r)**. As the reviewer recommends, we also explore the fiber diameter data by fiber type in gastrocnemius. Interestingly, BAIBA induced fiber-remodelling in the gastrocnemius included increasing the number of type IIa fibers, increasing the myofiber diameter, and specifically increasing the myofiber diameter of the hybrid type I/IIx and type IIx fibers.

"*We then applied our immunofluorescence approach to determine the effect of BAIBA on the mixed fiber composition of the gastrocnemius muscle (Supplementary Fig 1n). BAIBA did not significantly increase the total myofiber number in gastrocnemius (Supplementary Fig 1o). However, BAIBA induced fiber remodelling, significantly increasing the number of fatigue resistant type IIa fibers in gastrocnemius (Supplementary Fig 1p). BAIBA also significantly increased myofiber diameter (Supplementary Fig 1q) with a specific increase in the diameter of type I/IIx hybrid fibers (Supplementary Fig 1r).*"

Following the reviewer's recommendation, we have now also added data showing the fiber area by fibertype in the soleus of the 6-week BAIBA-treated mice in new **Figure 1k** (**page 5**). These data demonstrate that BAIBA specifically increased the area of type I and hybrid type I/IIa and I/IIx fibers in soleus (**page 5**):

"*BAIBA specifically increased the area of type I and hybrid type I/IIa and I/IIx fibers (Fig. 1k).*"

3.) The gene expression analysis of the HFD experiment was the only dataset in which a BAIBA-dependent change in mitochondrial genes was reported. However, in all contexts, increased mitochondrial number and/or function was observed. How do the authors explain these results in absence of regulation of mitochondrial genes? How would mitochondrial biogenesis (or fission) be brought about in the absence of corresponding markers and pathways? Are mitochondrial proteins seen at higher levels? More light should be shed on this issue.

In response to the reviewer's comment, we have performed additional analyses of our RNAseq datasets. Using Enrichr to perform Gene Set Enrichment Analysis (GSEA)[17] of the significantly differentially expressed genes in our RNAseq data sets of the soleus of mice i) treated with BAIBA for 6 weeks (**Page 8, new Figure 3c**) and ii) our BAIBA-treated mouse model of high fat diet obesity-induced muscle dysfunction (**Page 12**, **new Figure 5l**), searching against the MSigDB Molecular Signatures Database[18].

This analysis is consistent with our functional and molecular phenotyping and both datasets confirm enrichment of genes in the oxidative phosphorylation term related to the mitochondrial phenotype.

In the i) soleus from 6 week BAIBA-treated mice key GSEA terms are significantly enriched including myogenesis, oxidative phosphorylation, and fatty acid metabolism (**Page 8, new Figure 3c**):

"*Performing Gene Set Enrichment Analysis (GSEA)28 of the significantly differentially expressed genes searching against the MSigDB Molecular Signatures Database29 identifies that BAIBA induces transcriptional processes consistent with our observations and indicative of skeletal muscle structural and metabolic remodelling including mesenchymal remodelling (adjusted p-value 8.45e-29), myogenesis (adjusted p-value 8.45e-29), oxidative phosphorylation (adjusted p-value 9.42e-15) and fatty acid metabolism (adjusted p-value 1.79e-9) (Fig. 3c).*"(**Figure 3c**):

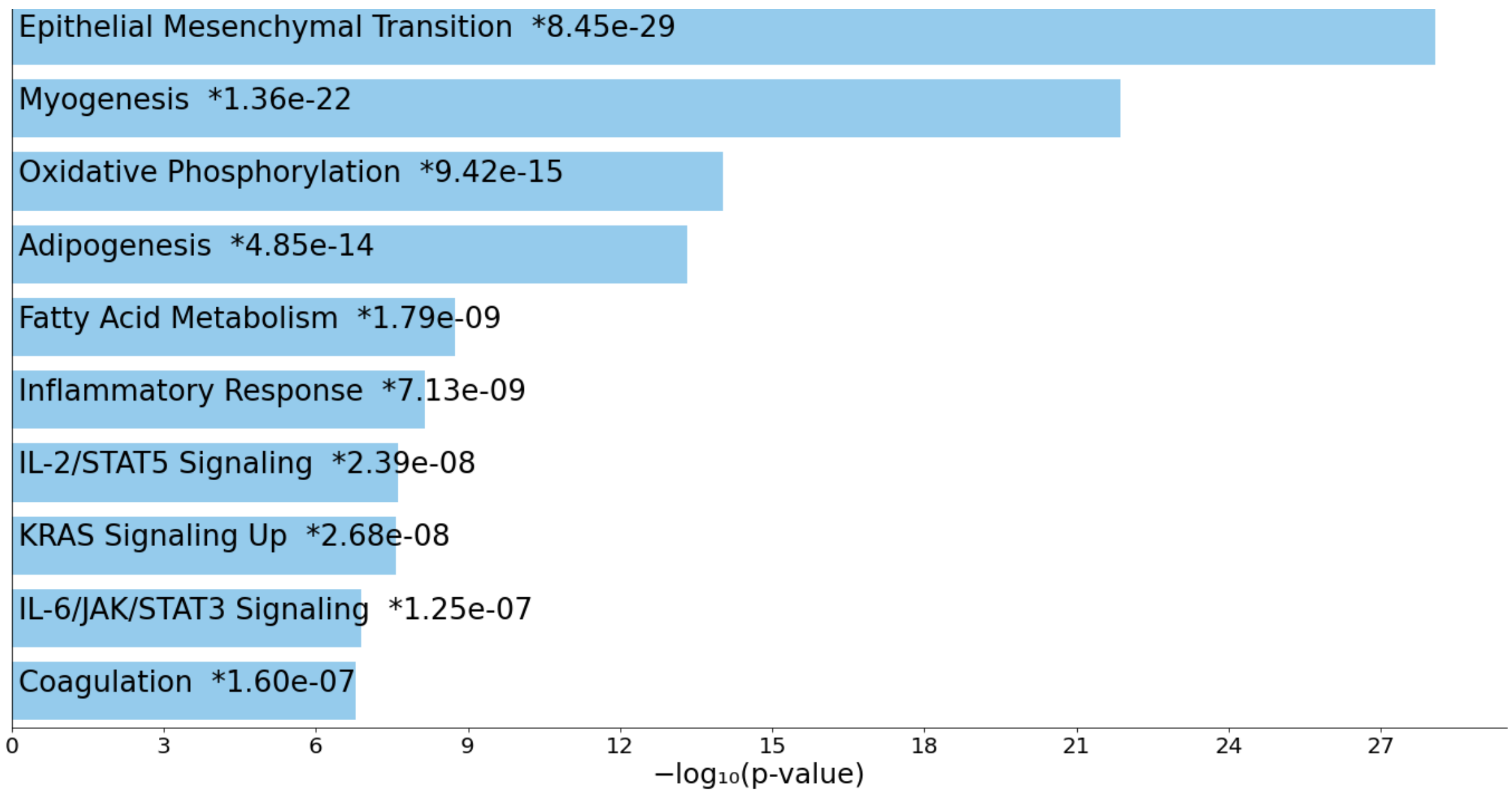

In the ii) soleus from the BAIBA-treated mouse model of high fat diet obesity-induced muscle dysfunction, key GSEA terms overlapping with the i) 6 week BAIBA-treated mouse experiment are significantly enriched including oxidative phosphorylation, mesenchymal remodelling and myogenesis (**Page 12**, **new Figure 5l**):

"Again, we performed GSEA[28] of the significant DEGs searching against the MSigDB Molecular Signatures Database[29]. This analysis identified that BAIBA induces transcriptional processes in the HFD-fed mice consistent with the earlier observations made in our 6 week treatment model. BAIBA induced transcriptional remodelling indicative of skeletal muscle structural and metabolic remodelling including oxidative phosphorylation (adjusted p-value 3.65e-20), mesenchymal remodelling (adjusted p-value 4.76e-15), and myogenesis (adjusted p-value 1.19e-8), (Fig. 5l)." **Figure 5l**:

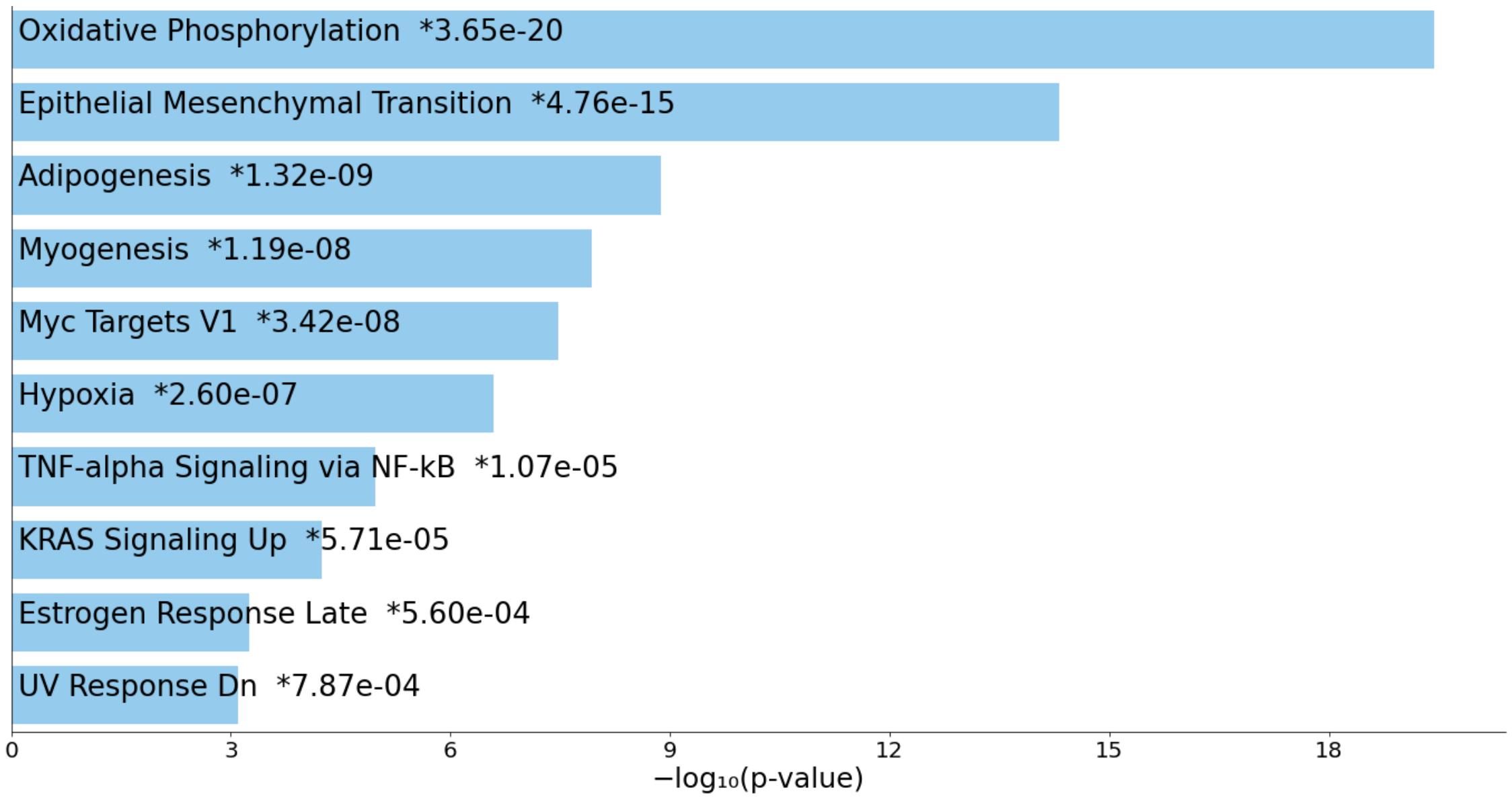

The oxidative phosphorylation term relating to mitochondrial function is enriched in both of these sets of data.

In addition, we have included new data describing immunoblotting of the mitochondrial electron transport chain respiratory protein complexes (Complex I – V) from the soleus, EDL and gastrocnemius of our 6 week BAIBA treated mice (**Page 6, Figure 1g, Supplementary Fig 1t**). We found that BAIBA significantly increased expression of Complexes I-V in all three muscle tissues (**Page 6, Figure 1g, Supplementary Fig 1t**):

"*Using immunoblotting we identify that BAIBA treatment increased the protein levels of all mitochondrial electron transport respiratory complexes (complex I – V) in soleus (Fig 1q; Supplementary Fig 1t) as well as EDL and gastrocnemius muscles (Supplementary Fig 1t).*"

4.) In the exercise plus BAIBA experiment (Figure 2), ex vivo contractility was performed, yet the results of fatigability not displayed. This would be the main expectation based on the reported fiber type shift. Moreover, endurance training might not be expected to increase peak force, in particular at the highest frequencies. How do the authors reconcile this with the results in Figure 1 and 2?

We agree with the reviewer regarding the importance of showing the fatigue data for the soleus of exercise training with BAIBA supplementation (**Page 7, Figure 2**) and thank them for pointing out our oversight. We have now added the soleus fatigue data for this experiment as **Figure 2e, page 7**:

"*The soleus fatigue resistance in BAIBA-treated and exercised mice was also greater than in the exercise only group (Fig 2e)*."

We agree with the reviewer that adaptation to exercise is a complex physiological process regulated by multiple molecular signals. We would not expect BAIBA alone to replicate all the aspects of endurance training exactly. Where BAIBA does appear to phenocopy endurance exercise is in the effects on skeletal muscle mitochondrial number, mitochondrial function, remodelling towards oxidative fibres and contractile fatigue resistance. However, BAIBA also increases soleus, TA and gastrocnemius muscle mass (**page 5, Figure 1, Supplementary Fig. 1l**) through effects on hyperplasia (**soleus page 5, Figure 1h**) but also effects on hypertrophy with increased fiber diameters (**soleus page 5, Figure 1i and Figure 1k**) (**gastrocnemius, page 5-6, Supplementary Figure 1q-r**). As we show in our newly provided data introduced above, BAIBA increases fiber diameter of type I and hybrid type I/IIa and I/IIx fibers in soleus and type I/IIx hybrid fibres in gastrocnemius. This hypertrophy may contribute to increased absolute peak force.

5.) The lack of difference in running wheel behavior between the light and the dark phase for the control animals in Figure 1a-d is puzzling. Wouldn't a clearer difference be expected based on the nocturnal behavior of mice?

We would like to reassure the reviewer that we do see the expected difference in wheel running behaviour between the light and dark phase for control animals. For total wheel rotations plotted in **Figure 1a** the statistical difference is masked by the scale of effect induced by BAIBA. For the benefit of the reviewer, we have replotted a bar graph of the total wheel rotations in the light phase and dark phase plotting the data for the control animals only. Total wheel rotations in the dark phase are almost twice that of the light phase in control animals:

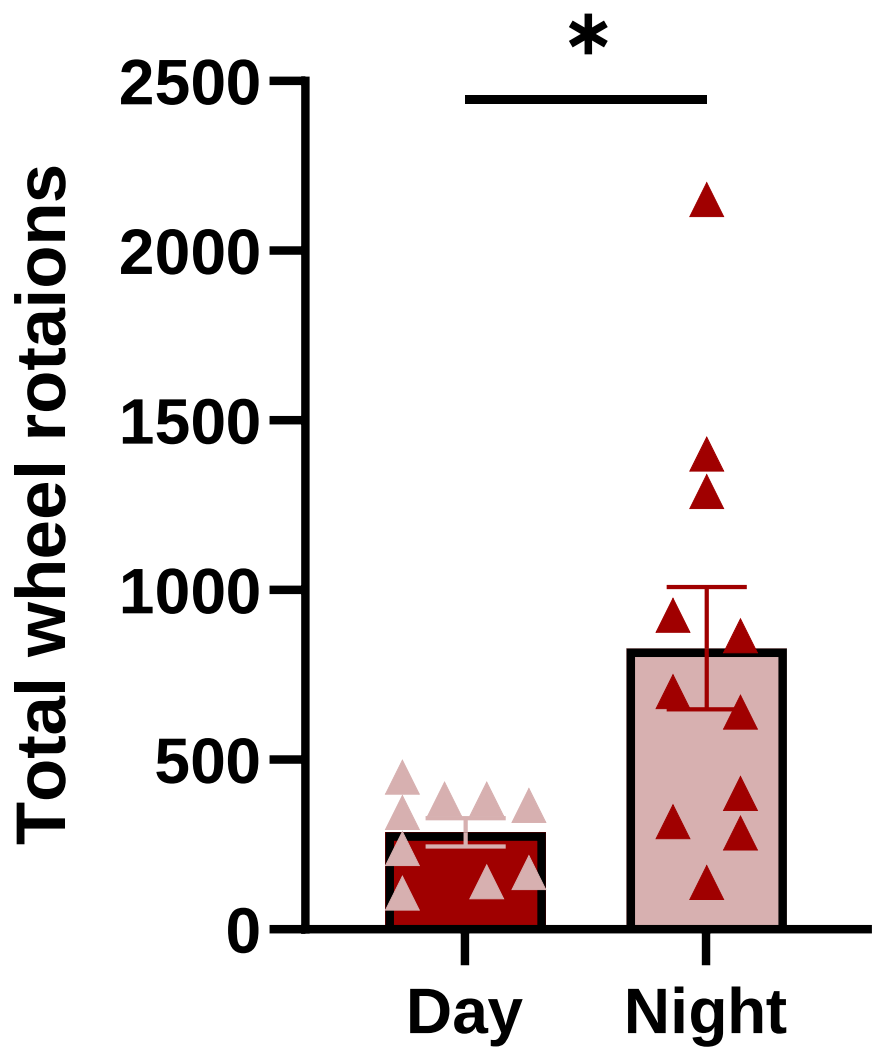

We have now included more granular additional data from our free wheel running phenotyping data to show the total number of exercise bouts the animals perform in either the Light or the Dark phase (**Page 5**, **Supplementary Figure 1j**) and the total average duration of all exercise bouts (**Page 5, Supplementary Figure 1k**):

"*However, BAIBA treatment does not affect the total number of exercise bouts the animals perform (Supplementary Fig 1j) or the total average duration of all exercise bouts (Supplementary Fig 1k).*"

Together, these additional data confirm that control animals do behave as expected in the dark phase with increased wheel rotations driven by increased markers of behavioural activity but not markers of performance (speed, rotations per bout).

Reviewer #3 (Remarks to the Author):

This manuscript from Daou and colleagues details the interesting findings the valine metabolite, b-aminoisobutyric acid (BAIBA), exerts several exercise-induced changes in muscle physiology. Included in this work are evidences of metabolic improvements in mice with BAIBI supplementation and human correlations of BAIBA following exercise. Mechanistically, the authors identify the involvement of PPARdelta (PPARd) into these effects. While the data are very interesting and potentially identify a major component of exercise-induced benefits, several concerns exist about the manuscript in its current state:

We thank the reviewer for their support and interest in our study. We also thank them for their insightful comments, which by addressing directly we feel have greatly improved the manuscript.

1) Overall, the manuscript is very difficult to follow. Several key details are missing from the text, and the figures are organized in a grid pattern that depicts the data in inconsistent graph styles and blurry IF images that are too small to discern detail (scale bars are also missing). Together, these issues make it difficult to accurately interpret the data. For example, the experiment pertaining to Figure 1 was not clear as to if this was an exercise study with prolonged running wheel access or an acute running wheel evaluation without appropriate acclimation sessions. Experiment 2, on the other hand is clearly a voluntary exercise study. The manuscript would be much improved if it were reorganized to effectively convey the information- for instance, a brief schematic of experimental design for each figure would clarify the study design.

In response to the reviewer's comments, we now incorporate key information from our methods section into the text of our results section to clarify the approaches used. We have edited our figures, removing the grid array and ensuring a consistent graphing style (except where this is mandated by the journal style guide, for example, summary data graphs such as box and whisker where n is greater than 10). Unfortunately, our IF images in the previous version suffered a loss of resolution due to the compression required to upload the figures. We have now enlarged these for clarity, maintaining resolution.

In addition, we have directly incorporated the reviewer's suggestion to include study design schematics for several of our key murine studies including:

1) Our 6-week BAIBA treatment study (**Page 5, Supplementary Figure 1a**)-

"*Six week old male C57BL6/J mice were treated with 100 mg/kg/day BAIBA in drinking water for 6 weeks using our protocol*[19] *(Supplementary Fig 1a).* "

2) Our high fat diet-obesity induced muscle dysfunction BAIBA-treatment model (**Page 11, Supplementary Figure 5c**)-

   "*We investigated whether BAIBA counters obesity and T2D-induced skeletal muscle dysfunction and exercise intolerance. C57BL6/J mice were either fed a chow or 60% high fat diet (HFD) for 8 weeks. Mice were then subcategorized with half the chow and HFD-fed mice given 100 mg/kg/day BAIBA for a further 14 weeks. The remaining mice continued on either the chow or HFD regimen alone. This study design modelled a therapeutic intervention (Supplementary Figure 5c).*"

3) Our muscle Abat knockdown and exercise training model (**Page 17, Supplementary Figure 10a**)-

"*We investigated the importance of skeletal muscle L-BAIBA synthesis for exercise performance in response to training and muscle adaptation to exercise. Age-matched C57BL6/J mice were divided into an unexercised group and a group that underwent a 6 week treadmill exercise training programme 4 days a week at 25 cm/s and 10% grade during a 45-minute session per day. These groups were further divided into groups receiving either scrambled short DNA antisense oligonucleotide (Gapmers) or short DNA antisense oligonucleotide against Abat, intramuscularly into the gastrocnemius muscles of both hind limbs twice a week to knockdown Abat expression (Supplementary Figure 10a).*"

2) There are several RNAseq datasets included in this study, however, all of these data are presented in supplemental figures with minimal analysis provide. Very important mechanistic information is likely to be found by more sophisticated analyses of these data, so it is recommended to enhance this work with better transcriptomic analyses. Also, are the RT-PCR results presented throughout the manuscript in agreement with the corresponding RNAseq data?

In response to the reviewer's comment, we have moved the majority of the RNAseq data to main figures. We have also performed new analyses using our RNAseq data. We now use Enrichr to perform Gene Set Enrichment Analysis (GSEA)[17] of the significantly differentially expressed genes in our four RNAseq data sets in the soleus of mice i) treated with BAIBA for 6 weeks (**Page 8, Fig 3c)**, ii) BAIBA-treated human skeletal muscle cells (**Page 10**, **Fig 4j**), iii) BAIBA-treated mouse model of obesity-induced muscle dysfunction (**Page 12, Fig 5l**) and iv) L-BAIBA-treated human skeletal muscle cells (**Page 15, Supplementary Fig 8f**), searching against the MSigDB Molecular Signatures Database[18]. These analyses were consistent with our functional, qPCR and molecular phenotyping.

In the i) soleus from 6-week BAIBA-treated mice key GSEA terms are significantly enriched including myogenesis, oxidative phosphorylation, and fatty acid metabolism (**Page 8, Fig 3c**):

"*Performing Gene Set Enrichment Analysis (GSEA)[28] of the significantly differentially expressed genes searching against the MSigDB Molecular Signatures Database[29] identifies that BAIBA induces transcriptional processes consistent with our observations and indicative of skeletal muscle structural and metabolic remodelling including mesenchymal remodelling (adjusted p-value 8.45e-29), myogenesis (adjusted p-value 8.45e-29), oxidative phosphorylation (adjusted p-value 9.42e-15) and fatty acid metabolism (adjusted p-value 1.79e-9) (Fig. 3c).*"

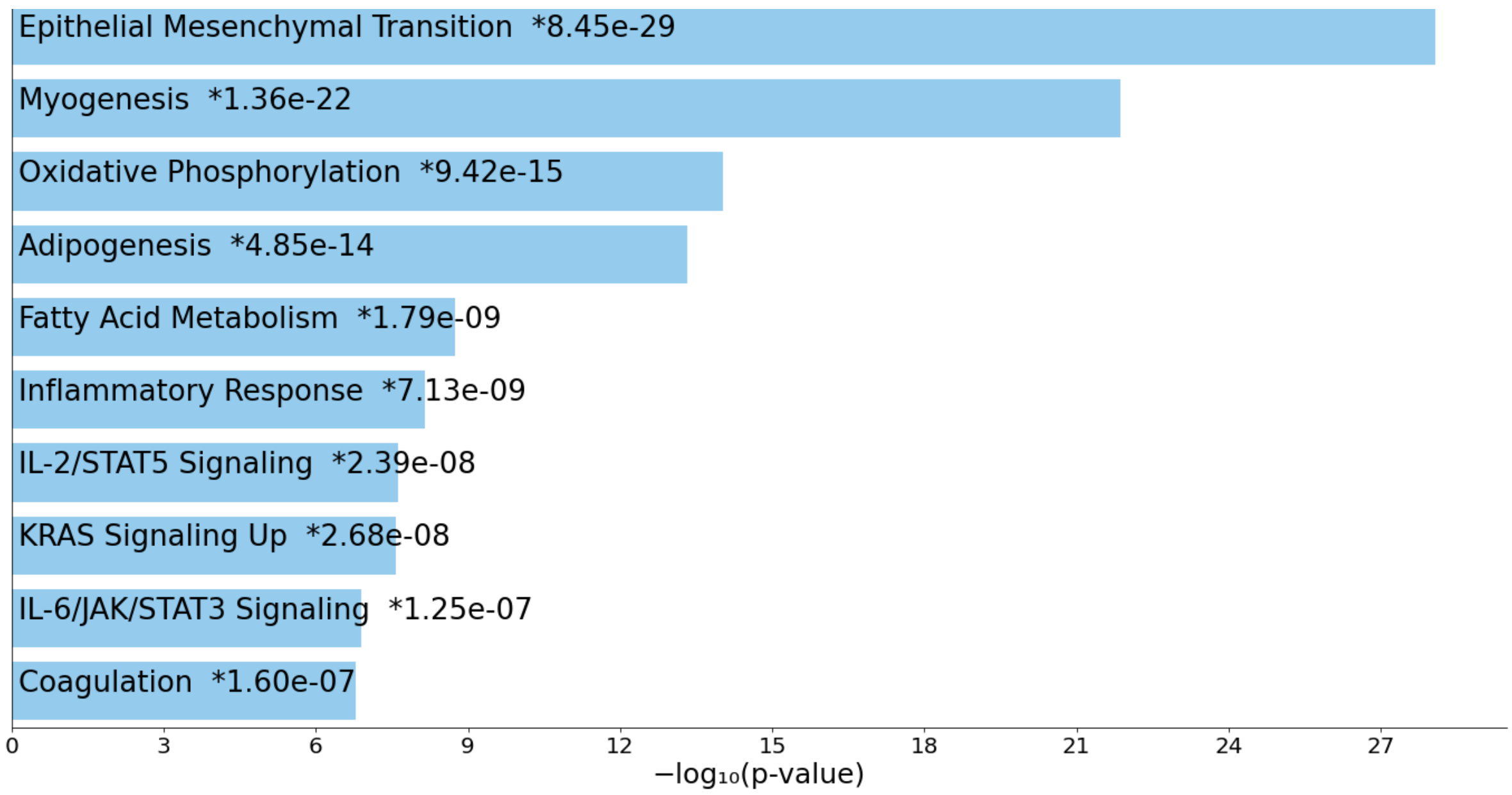

In the iii) soleus from the BAIBA-treated mouse model of high fat diet obesity-induced muscle dysfunction key GSEA terms overlapping with the i) 6-week BAIBA-treated mouse experiment are significantly enriched including oxidative phosphorylation, mesenchymal remodelling and myogenesis (**Page 12, Fig 5l**):

"*Again, we performed GSEA[28] of the significant DEGs searching against the MSigDB Molecular Signatures Database[29]. This analysis identified that BAIBA induces transcriptional processes in the HFD-fed mice consistent with the earlier observations made in our 6 week treatment model. BAIBA induced transcriptional remodelling indicative of skeletal muscle structural and metabolic remodelling including oxidative phosphorylation (adjusted p-value 3.65e-20), mesenchymal remodelling (adjusted p-value 4.76e-15), and myogenesis (adjusted p-value 1.19e-8), (Fig. 5l).*"

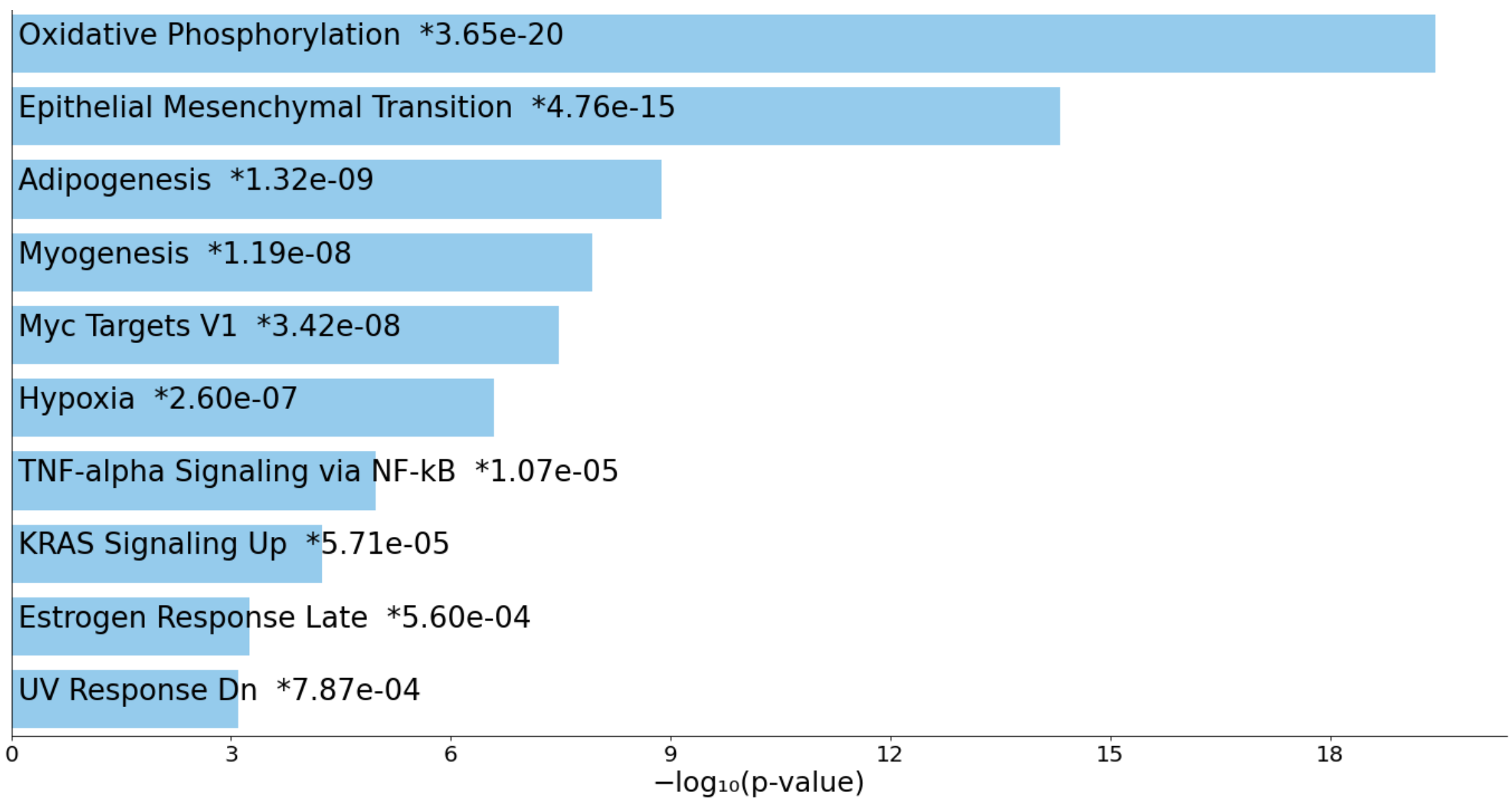

Focussing on our RNAseq analysis on human primary myotubes treated with L-BAIBA (**Page 15-16, Supplementary Figure 8**) using Enrichr to perform GSEA[17] of the significantly differentially expressed genes of both L-BAIBA (**Page 15-16, Supplementary Figure 8)** and

enantiomeric BAIBA (**Page 9, Figure 4**) gave very consistent results with terms related to mesenchymal transition and myogenesis.

Results of BAIBA treatment of human primary skeletal myotubes (**page 9, Figure 4j**):

"*Performing Gene Set Enrichment Analysis (GSEA)[28] of the significantly differentially expressed genes in BAIBA treated human skeletal myotubes, again using the MSigDB Molecular Signatures Database[29], identifies that BAIBA induces transcriptional processes consistent with our observations in vivo and indicative of skeletal muscle structural remodelling including mesenchymal remodelling (adjusted p-value 1.63e-53), and myogenesis (adjusted p-value 3.87e-13) (Fig 4j).*"

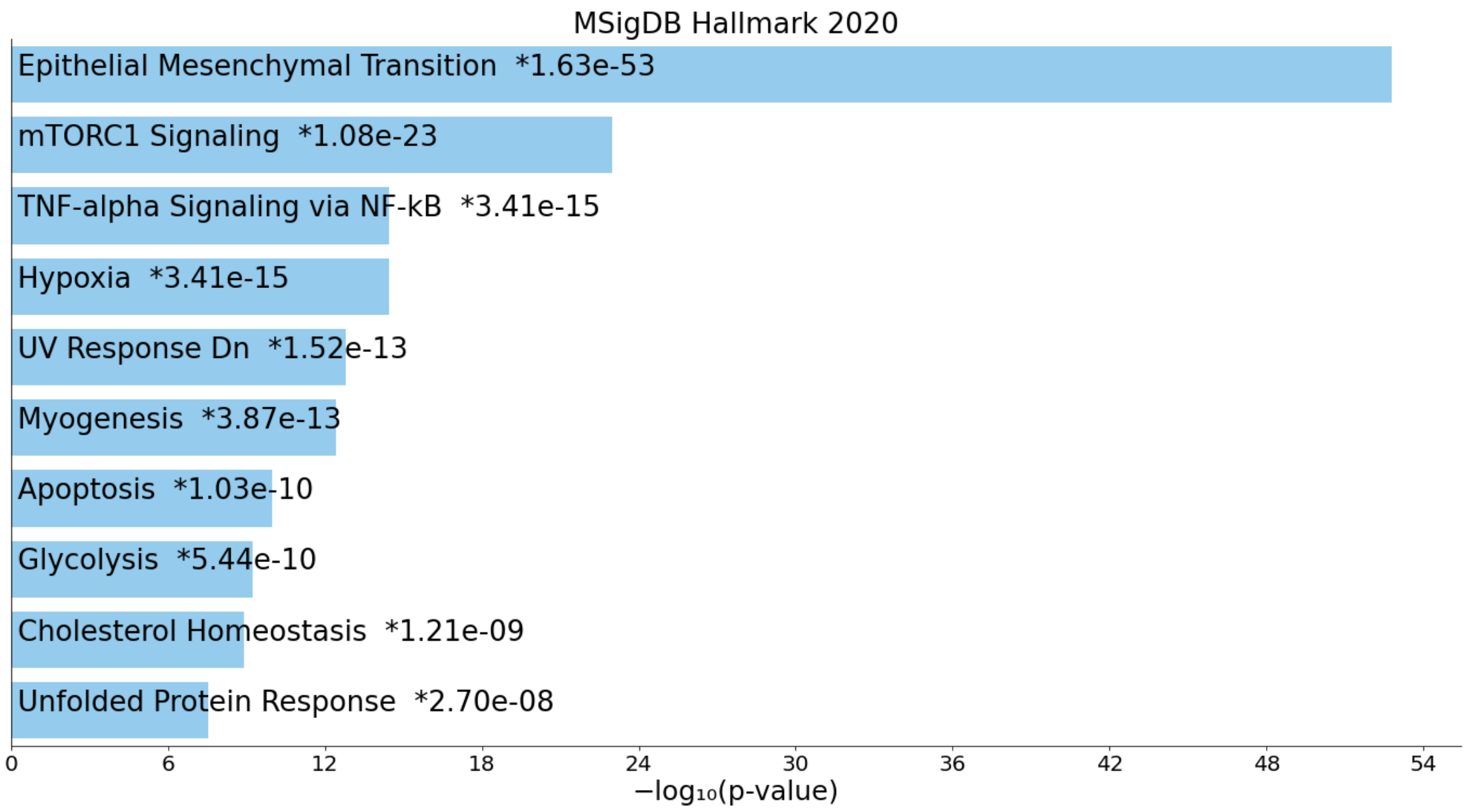

Results of L-BAIBA treatment of human primary skeletal myotubes (**page 15-16, Supplementary Fig 8f**):

"*Using GSEA[28] of the significant DEGs searching against the MSigDB Molecular Signatures Database[29] (Supplementary Fig 8f), we found that the top 2 most enriched terms with L-BAIBA treatment, mesenchymal remodelling ($1.68e^{-54}$) and myogenesis ($4.89e^{-28}$) were consistent with our in vivo results for BAIBA induced transcriptional processes in muscle of both our HFD-fed mice and our 6 week BAIBA treatment model and our previous study of human primary myotubes*."

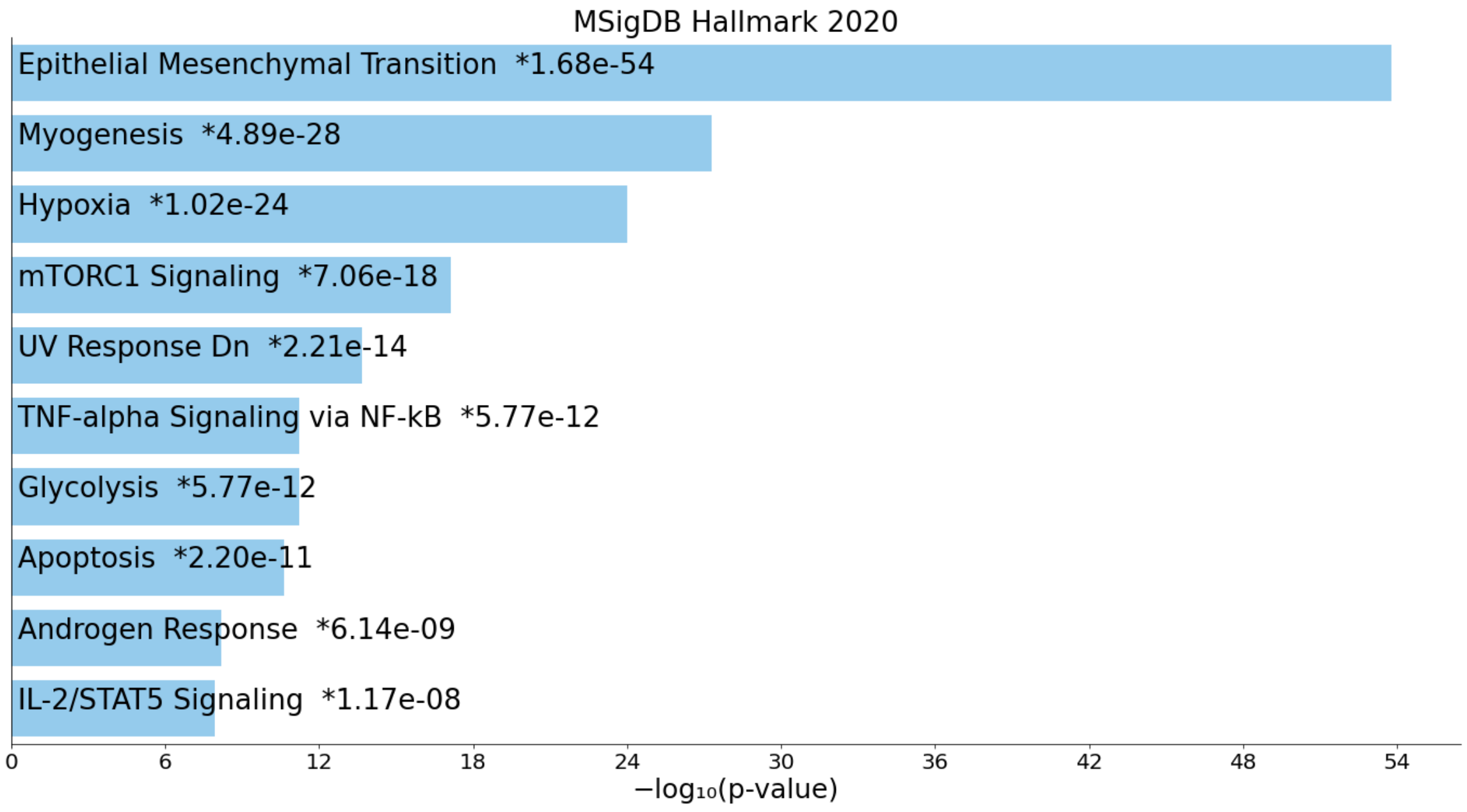

Consistently, mesenchymal and extracellular matrix-related muscle remodelling is a hallmark of skeletal muscle remodelling to endurance exercise[22, 23].

3) For the human myoblast studies, measures of proliferation and fusion are implied to represent exercise-like phenotypes. This is very misleading and not representative of any aspect of endurance exercise in vivo.

We apologise for our lack of clarity. It was not our intention to present the increased proliferation and differentiation response of the human myoblasts as an exercise-like phenotype, but rather the metabolic effect of BAIBA on mitochondrial function and efficiency and the enhanced expression of markers of oxidative fiber type canonically seen in fiber remodelling. We have addressed this in the text by separating out these distinct responses into two independent sections, one addressing the proliferation and differentiation phenotype (**Page 9**) and another examining the exercise training-like metabolic response initiated by BAIBA (**Page 10**).

Page 10:

BAIBA induces exercise-like metabolic adaptation in human primary skeletal myocytes

"Endurance exercise training increases muscle mitochondrial function[31, 32] and remodels fibers towards a more oxidative phenotype[33]. Therefore we examined the mitochondrial respiration of differentiated myotubes treated with BAIBA (10 µM) using a Seahorse bioanalyzer. BAIBA increased basal oxygen consumption and ATP production, and decreased spare respiratory capacity in human myotubes (Fig. 4k). BAIBA also increased myotube coupling efficiency (Fig. 4l). BAIBA increased the expression of markers of terminal differentiation towards oxidative muscle fibers in the human myotubes including MYH7, carnitine palmitoyl transferase 1b (CPT1b) and MYH2 (Fig. 4m). BAIBA increases oxidative metabolism and mitochondrial function in human myotubes in vitro."

Alternatively, these measures of myoblast behavior are more appropriately associated with muscle regeneration. In this context, it would be very interesting to test how BAIBA affects muscle regeneration in vivo.

We thank the reviewer for the interesting suggestion. Although not the primary focus of our study, we have now added a new murine experiment exploring the effect of BAIBA on hallmarks of skeletal muscle regeneration in vivo (**Page 10, Supplementary Fig 5a & 5b**). We investigated the effect of BAIBA on muscle regeneration in a mouse model of muscle injury. C57BL6J mice were either treated with 100 mg/kg/day BAIBA in drinking water for 1 week, or remained untreated. The mice were injected contra-laterally with cardiotoxin (50 µL, 10 µM CTX) into TA muscle of the left leg leaving the right leg uninjured. BAIBA-treated mice then continued with the 100 mg/kg/day BAIBA treatment regimen for a further 2 weeks[10]. Although TA mass was higher in injured limbs compared with uninjured limbs of BAIBA-treated mice (**Supplementary Figure 5a**), the percentage of centralized nuclei in the TA of the injured limb did not differ between BAIBA-treated and control mice (**Supplementary Figure 5b**). **Page 10:**

"***The effect of BAIBA on markers of muscle regeneration in a mouse skeletal muscle injury model***

BAIBA initiated a myogenic programme in skeletal muscle in vivo and induced proliferation and differentiation in human myoblasts. These may be hallmarks of muscle regeneration34. Therefore, we investigated the effect of BAIBA on muscle regeneration in a mouse model of muscle injury. C57BL6 mice were either treated with 100 mg/kg/day BAIBA in drinking water for 1 week, or remained untreated. The mice were injected contra-laterally with cardiotoxin (50 µL, 10 µM CTX) into TA muscle of the left leg leaving the right leg uninjured. BAIBA-treated mice then continued with the 100 mg/kg/day BAIBA treatment regimen for a further 2 weeks19. TA mass was increased to a greater extent in injured limbs compared with uninjured limbs of BAIBA-treated mice (Supplementary Figure 5a). However, the percentage of centralized nuclei in the TA of the injured limb did not differ between BAIBA-treated and control mice (Supplementary Figure 5b)."

4) In the ABAT-depletion experiment (Figure 4), the decrease in exercise capacity and adaptability suggests mitochondria decrements are occurring as a result of the knockdown treatment. This may be independent of BAIBA production. Proper interpretation of this experiment requires knowing whether or not mitochondrial content is negatively affected before the start of exercise adaptation testing.

For the reviewer's benefit, the ABAT-depletion experiment is now **Page 17**, **Figure 9** in the revised manuscript.

In response to the reviewer's comment regarding whether Abat knockdown negatively affects mitochondrial content independent of exercise we would like to highlight to the reviewer our experiments in **Fig 9i (Page 18)** showing mitochondrial functional analysis using high-resolution respirometry and **Fig 9j** (**Page 18**) mitochondrial content analysis using citrate synthase analysis of the soleus muscle from Abat-depleted and control mice with and without exercise training.

The control and Abat groups represent animals that received either scrambled control Antisense LNA GapmeR (control group) or Antisense LNA GapmeR against *Abat* (Abat group) into their hindlimb gastrocnemius muscle over a 6 week period. Their corresponding exercise-training groups (i.e. groups that received the equivalent injections but underwent 6 weeks of treadmill exercise training) are represented by the Control Ex and Abat Ex groups. These data demonstrate Abat knockdown in muscle (independent of any exercise training) does not significantly reduce soleus mitochondrial function (**Fig 9i**) or mitochondrial content

(**Fig 9j**) in the non-exercise trained Abat group. Therefore, we demonstrate that mitochondrial content (or function) is not negatively affected by Abat knockdown alone.

Contrastingly, exercise training increased mitochondrial content in exercise-trained controls (**Fig 9j**), whereas this increase was impaired in exercise-trained mice with Abat knockdown (**Fig. 9j**) alongside impaired levels of circulating BAIBA. These results demonstrate that the reduction in exercise-induced mitochondrial adaptation in Abat knockdown mice is not due to pre-existing mitochondrial deficits, but rather reflects impaired adaptive responses.

1. Hou H*, et al.* Gene expression profiling of puberty-associated genes reveals abundant tissue and sex-specific changes across postnatal development. *Hum Mol Genet* **26**, 3585-3599 (2017).

2. Pann P, de Angelis MH, Prehn C, Adamski J. Mouse Age Matters: How Age Affects the Murine Plasma Metabolome. *Metabolites* **10**, (2020).

3. Brust V, Schindler PM, Lewejohann L. Lifetime development of behavioural phenotype in the house mouse (Mus musculus). *Front Zool* **12 Suppl 1**, S17 (2015).

4. White RB, Bierinx AS, Gnocchi VF, Zammit PS. Dynamics of muscle fibre growth during postnatal mouse development. *BMC Dev Biol* **10**, 21 (2010).

5. Duddy W*, et al.* Muscular dystrophy in the mdx mouse is a severe myopathy compounded by hypotrophy, hypertrophy and hyperplasia. *Skelet Muscle* **5**, 16 (2015).

6. Xing Z*, et al.* lncRNA directs cooperative epigenetic regulation downstream of chemokine signals. *Cell* **159**, 1110-1125 (2014).

7. Viereck J*, et al.* Long noncoding RNA Chast promotes cardiac remodeling. *Sci Transl Med* **8**, 326ra322 (2016).

8. Adriaens C*, et al.* p53 induces formation of NEAT1 lncRNA-containing paraspeckles that modulate replication stress response and chemosensitivity. *Nat Med* **22**, 861-868 (2016).

9. Taniguchi T*, et al.* Novel use of a chemically modified siRNA for robust and sustainable in vivo gene silencing in the retina. *Sci Rep* **10**, 22343 (2020).

10. Roberts LD*, et al.* beta-Aminoisobutyric Acid Induces Browning of White Fat and Hepatic beta-Oxidation and Is Inversely Correlated with Cardiometabolic Risk Factors. *Cell Metab* **19**, 96-108 (2014).

11. Stautemas J*, et al.* Acute Aerobic Exercise Leads to Increased Plasma Levels of R- and S-beta-Aminoisobutyric Acid in Humans. *Front Physiol* **10**, 1240 (2019).

12. Knuiman P, van Loon LJC, Wouters J, Hopman M, Mensink M. Protein supplementation elicits greater gains in maximal oxygen uptake capacity and stimulates lean mass accretion during prolonged endurance training: a double-blind randomized controlled trial. *Am J Clin Nutr* **110**, 508-518 (2019).

13. Sarzynski MA*, et al.* The HERITAGE Family Study: A Review of the Effects of Exercise Training on Cardiometabolic Health, with Insights into Molecular Transducers. *Med Sci Sports Exerc* **54**, S1-S43 (2022).

14. Kitase Y*, et al.* beta-aminoisobutyric Acid, l-BAIBA, Is a Muscle-Derived Osteocyte Survival Factor. *Cell Rep* **22**, 1531-1544 (2018).

15. Picard M*, et al.* Mitochondrial structure and function are disrupted by standard isolation methods. *PLoS One* **6**, e18317 (2011).

16. Bowen TS*, et al.* Effects of Endurance Training on Detrimental Structural, Cellular, and Functional Alterations in Skeletal Muscles of Heart Failure With Preserved Ejection Fraction. *J Card Fail* **24**, 603-613 (2018).

17. Xie Z*, et al.* Gene Set Knowledge Discovery with Enrichr. *Curr Protoc* **1**, e90 (2021).

18. Castanza AS, Recla JM, Eby D, Thorvaldsdottir H, Bult CJ, Mesirov JP. Extending support for mouse data in the Molecular Signatures Database (MSigDB). *Nat Methods* **20**, 1619-1620 (2023).

19. McNally BD*, et al.* Long-chain ceramides are cell non-autonomous signals linking lipotoxicity to endoplasmic reticulum stress in skeletal muscle. *Nat Commun* **13**, 1748 (2022).

20. Whitehead A*, et al.* Brown and beige adipose tissue regulate systemic metabolism through a metabolite interorgan signaling axis. *Nat Commun* **12**, 1905 (2021).

21. Palkar PS*, et al.* Cellular and pharmacological selectivity of the peroxisome proliferator-activated receptor-beta/delta antagonist GSK3787. *Mol Pharmacol* **78**, 419-430 (2010).

22. Timmons JA*, et al.* Modulation of extracellular matrix genes reflects the magnitude of physiological adaptation to aerobic exercise training in humans. *BMC Biol* **3**, 19 (2005).

23. Makhnovskii PA, Bokov RO, Kolpakov FA, Popov DV. Transcriptomic Signatures and Upstream Regulation in Human Skeletal Muscle Adapted to Disuse and Aerobic Exercise. *Int J Mol Sci* **22**, (2021).

Response to Reviewers' Comments

Reviewer #1 (Remarks to the Author):

The authors have performed several new experiments in response to the reviewers' comments. The amount of data and number of new experiments is impressive. The human studies are more comprehensive. Experiments using the Abat knockout mice showing reduced performance, time to exhaustion, running to exhaustion, etc. further support that it is L-, not D-BAIBA that is responsible for the majority of positive effects on muscle. The authors are to be commended.

We thank the reviewer for their supportive comments and appreciation of the additional data provided.

The authors finding that L, not D BAIBA is responsible for the majority of positive effects of the BAIBA mixture has been included in the abstract, however, nothing is mentioned about the enantiomers in the Introduction. It is suggested that the authors provide such information to the readers.

We thank the reviewer for their suggestion. We have edited our introduction on page 4 to mention our findings related to the L-enantiomer of BAIBA:

"*Here, we demonstrate that the L-enantiomer of BAIBA has autocrine and paracrine signalling effects in skeletal muscle which are required for exercise-mediated adaptive responses, including fiber-type remodelling, increased mitochondrial number and function, muscle hypertrophy and myotube differentiation*"

Line 90 page 5: It should be clarified that "BAIBA" is a mixture of the two enantiomers.

In response to the reviewer's comment, we have edited our manuscript on Page 5 to clarify BAIBA is an enantiomeric mix:

"*Enantiomeric D/L-BAIBA enhances voluntary exercise performance and skeletal muscle function in vivo***"**

Line 99, page 5: An increase in number of wheel rotations was observed in the 6 mo old males which is in contrast to the studies of Vallejo et al (PMID 4104589) where L-BAIBA did not increase the number of wheel rotations although it improved muscle and bone function and phenotype. This suggests that the positive effects of L-BAIBA diminish with aging.

We thank the reviewer for pointing out this interesting observation from the literature. Our data on page 5 describes the effects of BAIBA on 6 – 12 week old mice rather than 6 month old mice (Page 5, Figure 1a – c):

"*We used free wheel running analysis, preceded by a 3-day habituation period, to examine voluntary exercise performance in these mice. BAIBA treated mice exhibited significantly greater total wheel rotations (Fig. 1a), greater running speed (Fig. 1b) and greater number of rotations per exercise interval (Fig. 1c).*"

However, our data show that in older mice the effects of BAIBA on increased wheel rotations are maintained in mice between 3.5 and 7 months of age (page 11, Figure 5a):

"*To investigate the effect of BAIBA on voluntary exercise performance, we examined the wheel running response of these mice following a 3-day acclimation period, using CLAMS. In the chow fed condition the prolonged BAIBA-treated mice exhibited enhanced wheel running capacity (Fig. 5a).*"

Reviewer #2 (Remarks to the Author):

The authors have addressed all of my points and suggestions in an adequate manner.

We thank the reviewer for their constructive comments which have improved our manuscript.

Reviewer #3 (Remarks to the Author):

The authors have put considerable effort towards addressing the concerns of all of the reviewers in this revised manuscript. I have no further concerns and commend the authors in their responsiveness. Well done!

We thank the reviewer for their constructive and supportive comments and appreciation of the additional data provided which we believe has improved our manuscript.
